# A clinical practice guideline for the screening and assessment of enthesitis in patients with spondyloarthritis

**DOI:** 10.3389/fimmu.2022.978504

**Published:** 2022-09-12

**Authors:** Xinyu Wu, Dong Liu, Yanfei Li, Ya Xie, Liudan Tu, Yanli Zhang, Xi Zhang, Linkai Fang, Xiqing Luo, Zhiming Lin, Zetao Liao, Limin Rong, Jie Ren, Yuqi Zhou, Niansheng Yang, Jian Xu, Hua Zhang, Baijie Xu, Zhenbiao Wu, Feng Zhan, Zhenbin Li, Weiguo Xiao, Shengyun Liu, Yi Zhou, Shanhui Ye, Qing Lv, Lijun Zhang, Dongbao Zhao, Shanzhi He, Like Zhao, Lijun Wu, He Lin, Yunxiao Zhu, Donggeng Guo, Zehong Yang, Budian Liu, Kehu Yang, Jieruo Gu

**Affiliations:** ^1^ Department of Rheumatology, The Third Affiliated Hospital of Sun Yat-sen University, Guangzhou, China; ^2^ Evidence-Based Medicine Center, School of Basic Medical Sciences, Lanzhou University, Lanzhou, China; ^3^ Department of Spine Surgery, The Third Affiliated Hospital of Sun Yat-sen University, Guangzhou, China; ^4^ Department of Medical Ultrasonics, The Third Affiliated Hospital of Sun Yat-sen University, Guangzhou, China; ^5^ Department of Pulmonary and Critical Care Medicine, The Third Affiliated Hospital of Sun Yat-sen University, Guangzhou>, China; ^6^ Department of Rheumatology, The First Affiliated Hospital of Sun Yat-sen University, Guangzhou, China; ^7^ Department of Internal Medicine, First Affiliated Hospital of Kunming Medical University, Kunming, China; ^8^ Department of Rheumatology, The Fifth Affiliated Hospital of Sun Yat-sen University, Zhuhai, China; ^9^ Department of Rheumatology, Jieyang People’s Hospital, Jieyang, China; ^10^ Department of Rheumatology, Tangdu Hospital of Air Force Military Medical University, Xian, China; ^11^ Department of Rheumatology, Hainan General Hospital, Hainan Affiliated Hospital of Hainan Medical University, Haikou, China; ^12^ Department of Rheumatology, Bethune International Peace Hospital, People’s Liberation Army, Shijiazhuang, China; ^13^ Department of Rheumatology, The First Hospital of China Medical University, Shenyang, China; ^14^ Department of Rheumatology, The First Affiliated Hospital of Zhengzhou University, Zhengzhou, China; ^15^ Department of Rheumatology and Immunology, First Affiliated Hospital of Jinan University, Guangzhou, China; ^16^ Department of Rheumatology and Immunology, First Affiliated Hospital of Guangzhou Medical University, Guangzhou, China; ^17^ Department of Rheumatology, The Seventh Affiliated Hospital of Sun Yat-sen University, Shenzhen, China; ^18^ Department of Rheumatology, Shenzhen Hospital, The University of Hong Kong, Shenzhen, China; ^19^ Department of Rheumatology and Immunology, Changhai Hospital, Naval Medical University, Shanghai, China; ^20^ Department of Rheumatology, Zhongshan People’s Hospital, Zhongshan, China; ^21^ Department of Rheumatology and Immunology, Beijing Hospital, National Center of Gerontology, Institute of Geriatric Medicine, Chinese Academy of Medical Sciences, Beijing, China; ^22^ Department of Rheumatology and Immunology, The People’s Hospital of the Xinjiang Uygur Autonomous Region, Urumqi, China; ^23^ Department of Rheumatology, Fujian Provincial Hospital, Fuzhou, China; ^24^ Department of Medical Ultrasonics, The Seventh Affiliated Hospital of Sun Yat-sen University, Shenzhen, China; ^25^ Department of Rheumatology and Immunology, Ningxia Clinical Institute of Bone and Joint Research, The Affiliated People’s Hospital of Ningxia Hui Autonomous Region, Ningxia Medical University, Yinchuan, China; ^26^ Department of Radiology, Sun Yat-Sen Memorial Hospital, Sun Yat-Sen University, Guangzhou, China

**Keywords:** spondyloarthritis, enthesitis, screening, ultrasound, magnetic resonance imaging

## Abstract

**Objective:**

The aim of this review is to provide guidance on the selection of approaches to the screening and assessment of enthesitis in patients with spondyloarthritis (SpA).

**Methods:**

Twenty-four questions regarding the approaches to the screening and assessment of enthesitis and the implementation details were devised, followed by a systemic literature review. The Grading of Recommendations Assessment, Development, and Evaluation methodology was employed in the development of this guideline, with modifications to evaluate non-interventional approaches under comprehensive consideration of costs, accessibility, and evidence strength. A consensus from the voting panel was required for the inclusion of the final recommendations and the strength of each recommendation.

**Results:**

Seventeen recommendations (including five strong recommendations) were included in this guideline. The voting panel expressed unequivocal support for the necessity of screening and assessment of enthesitis in patients with SpA. It was agreed unanimously that symptom evaluation and physical examination should serve as the initial steps to the recognition of enthesitis, whereas Maastricht Ankylosing Spondylitis Enthesitis Score is a reliable tool in both clinical trials and daily medical practice. Ultrasound examination is another reliable tool, with power Doppler ultrasound as an informative addition. Notwithstanding its high resolution, MRI is limited by the costs and relatively low accessibility, whereas radiographs had low sensitivity and therefore should be rendered obsolete in the assessment of enthesitis. PET/CT was strongly opposed in the detection of enthesitis.

**Conclusion:**

This guideline provides clinicians with information regarding the screening and assessment of enthesitis in patients with SpA. However, this guideline does not intend on dictating choices, and the ultimate decisions should be made in light of the actual circumstances of the facilities.

## Introduction

Enthesis refers to the anatomic interface between tendons, ligaments, capsules, fascia, and bones, whereas enthesitis refers to the inflammation at such insertion sites (1, 2). Entheses could be classified as fibrous entheses and fibrocartilaginous entheses (3). Enthesitis is considered the hallmark and characteristic feature of spondyloarthritis (SpA) (4). Persistent enthesitis often leads to regional structural damage such as tendon injuries and bone erosions, and the subsequent repair process could give rise to the formation of enthesophytes and ultimately functional impairment of related anatomic structures (5). Although the importance of enthesitis in the etiology of SpA has been widely acknowledged, it is often overlooked in the management of patients with SpA in clinical practice (6). This guideline is dedicated to the screening and diagnosis of enthesitis in the specific population of patients with SpA, aiming to improve the understanding and awareness of enthesitis detection in rheumatologists.

## Methods

This guideline was developed using the Grading of Recommendations Assessment, Development, and Evaluation methodology to assess the quality of evidence and the levels of recommendations (7–9). The Core Team, Expert Panel, and Voting Panel generated 17 questions regarding the screening and diagnosis of enthesitis in patients with SpA and the corresponding approaches. The following methods to detect enthesitis were listed as potential approaches: history taking, clinical examination, ultrasound (US), MRI, X-ray, and PET-CT. A patient panel of five patients with SpA reviewed the evidence reports provided with an interpretation from a moderator and gave their personal perspectives. Systemic literature reviews (SLRs) and meta-analyses were conducted to address the questions. Search strategies and study inclusion processes could be found in **Supplementary Appendixes 3** and **4**.

We devised a framework dedicated to the evaluation of non-interventional approaches to the screening and diagnosis of SpA-related enthesitis. On the basis of the costs and accessibility, all the approaches were categorized as 1) inexpensive and easily accessible, 2) moderately costly and relatively accessible; and 3) expensive and difficult to gain access to. The strengths of each recommendation were classified as strong or conditional. A strong recommendation was given upon the consideration that the approach of screening and examining enthesitis could provide critical information that could educate and modify disease management options with relatively low costs and high accessibility. A conditional recommendation was given when moderate information could be gained with corresponding costs and accessibility. For approaches that are inexpensive and easily accessible, a strong recommendation was also given even in case of low certainty of evidence. For approaches that were moderately costly and relatively accessible, moderate certainty of evidence warranting its necessity was deemed sufficient to support a strong recommendation. For approaches that were expensive and rarely available, high certainty of evidence warrants a strong recommendation. Details of this framework could be seen in **Supplementary Appendix 1**.

Each question was rewritten into recommendation statement, which were sent to the Voting Panel along with the evidence reports. An online meeting was held, during which the Voting Panel reviewed the evidence reports and the recommendation statements and then voted for or against these recommendations. At least a consensus of 70% of the Voting Panel was required to determine the inclusion of the recommendations in this guideline.

This guideline only applies to patients with SpA, with or without symptomatic enthesitis. Enthesopathy resulting from aging, sports, or mechanical injuries was not addressed in this guideline.

## Results/recommendations

A summary of the recommendations along with the certainty of evidence is presented in **Table 1**.

**Table 1:** Recommendations for approaches pertaining to the screening and evaluation of enthesitis in patients with SpA.

No.	Recommendation	Certainty ofEvidence	Approval Rate	Level of Agreement, mean
1	Screening for enthesitis is STRONGLY RECOMMENDED for patients with SpA, with or without symptomatic enthesitis.	High	100%	9.39
*Symptom assessment*				
2	Inquiry about painful entheses is STRONGLY RECOMMENDED in history taking toward patients with SpA.	Low	100%	9.39
*Clinical examination*				
3	Clinical examination was STRONGLY RECOMMENDED in the assessment of enthesitis in patients with SpA.	Moderate	100%	9.30
4	Maastricht Ankylosing Spondylitis Enthesitis Score (MASES) was CONDITIONALLY RECOMMENDED in the clinical examination of enthesitis in patients with SpA.	Moderate	95.65%	8.70
5	Maastricht Ankylosing Spondylitis Enthesitis Score (MASES) was CONDITIONALLY RECOMMENDED in the assessment of therapeutic responses of enthesitis in patients with SpA.	Moderate	95.65%	8.61
*Ultrasound*				
6	Ultrasound examination is STRONGLY RECOMMENDED in the assessment of enthesitis in patients with SpA.	High	95.65%	9.22
7	Both gray scale ultrasound (GSUS) and power Doppler ultrasound (PDUS) are STRONGLY RECOMMENDED in the US examination of enthesitis in patients with SpA.	High	100%	8.87
8	The following entheses are CONDITIONALLY RECOMMENDED to be included in the US examination of enthesitis in patients with SpA: proximal plantar fascia, distal Achilles tendon, distal and proximal patellar ligament, distal quadriceps, brachial triceps tendons, common extensor tendons, and greater trochanter.	Moderate	100%	9.04
9	Madrid sonography enthesitis index (MASEI) is CONDITIONALLY RECOMMENDED in the US examination of enthesitis in patients with SpA.	Moderate	100%	8.35
10	US is CONDITIONALLY RECOMMENDED to monitor therapeutic responses of enthesitis in patients with SpA.	Moderate	86.96%	8.35
*MRI*				
11	MRI is CONDITIONALLY RECOMMENDED in the assessment of enthesitis in patients with SpA.	Low	100%	8.43
12	Whole-body MRI is CONDITONALLY RECOMMENDED AGAINST as the screening method for enthesitis in patients with SpA.	Low	91.30%	8.61
13	Ultrashort echo time (UTE) sequence is CONDITIONALLY RECOMMENDED in the MRI examination of enthesitis in patients with SpA.	Very Low	91.30%	8.35
14	Contrast-enhanced MRI is STRONGLY RECOMMENDED AGAINST in the MRI examination of enthesitis in patients with SpA.	Very Low	91.30%	9.04
15	The OMERACT Heel Enthesitis MRI Scoring System (HEMRIS) is CONDITIONALLY RECOMMENDED in the evaluation of heel enthesitis in patients with SpA.	Very Low	100%	8.43
*X-ray*				
16	Radiograph is CONDITIONALLY RECOMMENDED AGAINST in the assessment of enthesitis in patients with SpA.	Moderate	95.65%	8.96
PET/CT				
17	PET/CT is STRONGLY RECOMMENDED AGAINST in the assessment of enthesitis in patients with SpA.	Very Low	91.30%	9.04

### Screening for enthesitis is strongly recommended for patients with SpA, with or without symptomatic

On the basis of questions 1–3 in the evidence report (**Supplementary Appendix 6**), there was sufficient evidence to support a strong recommendation for the screening for enthesitis in patients with SpA. This recommendation was formulated upon the following three aspects of consideration: 1) Evidence of enthesitis could facilitate the diagnosis of SpA. Enthesitis was listed as a SpA feature in the Assessment in SpondyloArthritis international Society (ASAS) classification criteria for SpA, and its capacity of facilitating an early diagnosis of SpA was confirmed in a prospective study by D’Agostino et al. using power Doppler US (PDUS) to detect enthesitis, even when stratifying patients with or without peripheral symptoms (10). For patients suspected with SpA, findings of enthesitis during the screening process could improve the certainty of diagnosis for clinicians. 2) Clinical enthesitis composed a proportion of the disease burden for patients with SpA. Presence of symptomatic enthesitis is associated with pain, worse quality of life, impaired ability of daily activities, and work capacity (11–14). Screening for enthesitis in this subset of patients could ascertain the presence of enthesitis, differentiating enthesitis from other pathological conditions in adjacent structures (15). 3) Presence of enthesitis is potentially related to severity of disease. Formation of peripheral enthesophytes was associated with the presence and number of axial syndesmophytes, according to the DEvenir des Spondyloarthrites Indifferenciees Recentes (DESIR) cohort and a case-control study by Aydin et al. (16, 17). This finding indicated that peripheral enthesitis could be a marker of disease severity of SpA, even in patients without symptomatic enthesitis.

### Inquiry about painful entheses is strongly recommended in history taking toward patients with SpA.

Symptomatic enthesitis often presents pain at the entheses. Research indicated that there was a delayed diagnosis of approximately 8 years between the first onset of enthesitis symptoms and recognition of enthesitis (18). In clinical practice, asking patients about painful entheses during history taking could serve as the first steps toward recognition of enthesitis. A study showed that, in patients with SpA with long-term pain at the entheses, by using US as the reference standard, the sensitivity of history taking was 72%, whereas specificity was 63%. However, a large number of patients with SpA present asymptomatic enthesitis, whereas fibromyalgia was recognized in 45% of the patients with axial SpA, therefore complicating results of history taking (18). Painful entheses could not serve as confirming evidence of enthesitis, and further examination methods should be chosen on the basis of history taking so as to confirm the presence of enthesitis.

### Clinical examination was strongly recommended in the assessment of enthesitis in patients with SpA.

Clinical examination is the most common method in the assessment of enthesitis in patients with SpA, with the advantages of being simple and convenient to conduct and no need of examination equipment (19–21). However, clinical examination has a relatively low sensitivity compared with other imaging examinations, approximately 20% (**Supplementary Appendix 6**). Enthesitis could be identified in a large number of patients with SpA using imaging methods such as US or MRI, but tenderness could not be elicited during clinical examination (22, 23). On the other hand, tenderness at the entheses does not confirm the presence of enthesitis, because pain could be attributed to synovitis, arthritis, or other pathological conditions in adjacent structures (3). Therefore, clinical examination could only serve as a clue to the discovery of enthesitis, not confirming evidence of enthesitis. Considering that clinical examination is easy to operate in clinical practice, it is advised to apply clinical examination as an initial approach to the identification of enthesitis. After completion of clinical examination, US or MRI is advised as the subsequent examination approach.

### Maastricht Ankylosing Spondylitis Enthesitis Score was conditionally recommended in the clinical examination of enthesitis in patients with SpA

Common scoring methods of enthesitis clinical examination include Mander Enthesitis Index (MEI) (21), Maastricht Ankylosing Spondylitis Enthesitis Score (MASES) (19), Leeds Enthesitis Index (LEI) (24), Spondyloarthritis Research Consortium of Canada (SPARCC) Enthesitis Index (20), Berlin Index (25), and Gladman Index (24). Among these scoring methods, MASES is considered the most convenient and therefore the most widely applied in clinical practice and clinical trials (26–29). MASES scores the following 13 entheses: first costochondral joint, seventh costochondral joint, posterior superior iliac spine, anterior superior iliac spine, iliac crest, fifth lumbar spinous process, and proximal insertion of Achilles tendon, with each entheses rated as 0 (no tenderness) or 1 (tenderness). It should be noted that, by limiting the number of entheses evaluations, tenderness at certain entheses might be overlooked, because studies showed that 21% of patients with MEI > 0 were rated as 0 with MASES (19). Of all the scoring methods, MASES is considered a relatively convenient and less time-consuming one (6). An overview of the scoring indices of enthesitis with physical examination is presented in **Table 2**. This guideline endorsed MASES in the screening and assessment of enthesitis in patients with SpA on the account of feasibility and conveniences.

**Table 2:** Overview of physical examination systems used to assess enthesitis in patients with SpA (Ref. McGonagle et al., *Semin Arthritis Rheum*., 2021 Jul 9; 51(6): 1147–1161).

Index	Site Assessed	Scoring	Pros	Cons	Reference
Mander Enthesitis Index/Newcastle index	66 in total: nuchal crests, manubriosternal joint, costochondral joints, greater tuberosity and medial and lateral epicondyles of the humerus, iliac crests, anterior superior iliac spines, greater trochanter of the femur, medial and lateral condyles of the femur, insertion of the Achilles tendons and plantar fascia to the calcaneus, cervical, thoracic, and lumbar spinous processes, ischial tuberosities, and posterior superior iliac spines	Each site rated from 0 to 3 (where 0 = no pain, 1 = mild tenderness, 2 = moderate tenderness, and 3 = wince or withdraw). Some of the sites are scored individually whereas others are scored as a group; max total score = 90	Λ Comprehensive Λ Captures wide range of axial and peripheral sites Λ Validated in ankylosing spondylitis	Λ Time consuming Λ Potential overlap with fibromyalgia tender points Λ 0–3 scoring system could contribute to greater inter- and intra-rater inconsistency	Mander et al., *Ann Rheum Dis*, 1987; 46: 197–202
Maastricht Ankylosing Spondylitis Enthesitis Score	13 in total: first costochondral joint, seventh costochondral joint, posterior superior iliac spine, anterior superior iliac spine, iliac crest, fifth lumbar spinous process, and proximal insertion of Achilles tendon	Presence or absence of tenderness; max score = 13	Λ Recommended by ASAS Λ Fast Λ Simple Λ Widely used in clinical trials	Λ Omits commonly affected yet accessible axial sites Λ Omits commonly affected peripheral sites, except the Achilles tendon	Heuft-Dorenbosch et al., *Ann Rheum Dis*, 2003; 62: 127–132
SPARCC Enthesitis Index	16 in total: the greater trochanter, quadriceps tendon insertion into the patella, patellar ligament insertion into the patella and tibial tuberosity, Achilles tendon insertion, plantar fascia insertion, medial and lateral epicondyles, and the supraspinatus insertion	Presence or absence of tenderness; max score = 16	Λ Fast Λ Simple Λ Widely used in clinical trials	Λ Includes peripheral sites only	Maksymowych et al., *Ann Rheum Dis*, 2009; 68: 948–53
Leeds Enthesitis Index	6 in total: bilateral lateral epicondyles, medial femoral condyles, and Achilles tendon insertions	Presence or absence of tenderness; max score = 6	Λ Fast Λ Simple Λ Widely used in clinical trials	Λ Includes peripheral sites only	Healy and Helliwell, *Arthritis Rheum*, 2008; 59: 686–691
Gladman Index	6 in total: bilateral tibial tuberosity, plantar fascia and Achilles tendon insertion)	Presence or absence of tenderness; max score = 6	Λ Fast Λ Simple	Λ Seldom used Λ Omits commonly affected yet accessible axial sites	Healy and Helliwell, *Arthritis Rheum*, 2008; 59: 686–691
Berlin/Major Index	12 in total: iliac crest, proximal Achilles, greater trochanter, medial condyle femur, lateral condyle femur, and insertion plantar fascia	Presence or absence of tenderness; max score = 12	Λ Fast Λ Simple	Λ Seldom used Λ Omits commonly affected yet accessible axial sites	Polachek et al. *Arthritis Care Res*, 2017; 69: 1685–1691
University of California San Francisco Enthesitis Index	17 in total: vertebral processes of Cl-C2, C7-T1, T12-L1, L5-S1, symphysis pubis, both greater trochanters, pelvic abductor origin, anterior superior border of the iliac crests, ischial tuberosities, insertions of Achilles tendons, and plantar fascia	Each site rated from 0 to 3 (where 0 = no pain, 1 = mild tenderness, 2 = moderate tenderness, and 3 = wince or withdraw). Some of the sites are scored individually whereas others are scored as a group; max total score = 51	Λ Includes spinous processes	Λ Seldom used Λ 0–3 scoring system could contribute to greater inter- and intra-rater inconsistency Λ Omits key peripheral sites	Clegg et al., *Arthritis Rheum*, 1996; 39: 2004–2012

### Maastricht Ankylosing Spondylitis Enthesitis Score was conditionally recommended in the assessment of therapeutic responses of enthesitis in patients with SpA

Multiple clinical trials employed indices such as MASES and MEI as the study endpoints. Results of the clinical trials showed that, after treatment of csDMARDs or bDMARDs, there was a significant decrease of the MEI score as many as four points, whereas MASES decreased by one to two points (24, 26–32). BE AGILE and BE ACTIVE trials employing MASES as one of the endpoints showed that bimekizumab could lower the MASES index up to three units within 6 months (33, 34). FUTURE 2 and 3 studies also used MASES as one of the endpoints, demonstrating that secukinumab could provide sustained resolution of enthesitis (35). MASES, MEI, SPARCC, and other indices listed above could serve as reliable tools in the assessment of enthesitis therapeutic responses, yet MASES is by far the most widely employed. Upon considerations that MASES is simpler and less time-consuming in the context of clinical practice, this guideline endorsed MASES as the assessment tool of therapeutic responses in patients with SpA. This recommendation does not dictate choices of the assessment tool, and other indices could also be considered.

### Ultrasound examination is strongly recommended in the assessment of enthesitis in patients with SpA

Because US examination is relatively inexpensive and fairly accessible without exposure of radiation, US has become the first-line approach to assess enthesitis in recent years (1, 2). Compared with clinical examination and X-ray, US possessed a higher sensitivity in the detection of enthesitis, capable of detecting inflammatory lesions and structural lesions at the same time (22, 23, 36–38). Meta-analyses indicated that, compared with clinical examination, a significantly higher number of enthesitis lesions could be found using US (OR = 3.22, 95% CI [2.33, 4.45]) (**Supplementary Appendix 6**), suggesting that subclinical enthesitis is prevalent in patients with SpA. Systematic literature review showed that the sensitivity of US examination ranged from 50% to 90%, whereas specificity ranged from 60% to 90% (**Supplementary Appendix 6**). PDUS has a high specificity despite relatively low sensitivity (39, 40). It has been brought to attention that the diagnostic accuracy of US might depend on the individual experiences of the examiners (41, 42), but SLR showed that the inter-observer reliability of US was good with an ICC of 0.7–0.98 (**Supplementary Appendix 6**). Still, it is still advised to assign training sessions or workshops to the examiners in order to increase the diagnostic accuracy and consistency (41).

According to the OMERACT definitions, elementary lesions of enthesitis in US examinations include hypoechogenicity, increased thickness at enthesis, erosions, calcifications/enthesophytes, and Doppler signal at insertion (**Table 3**) (43).

**Table 3:** OMERACT definitions of elementary lesions of enthesitis upon ultrasound examinations.

Elementary Lesion	Definition
Hypoechogenicity	Lack of the homogeneous fibrillar pattern in the enthesis (<2 mm from the cortical bone) with loss of the tightly packed echogenic lines after correcting for anisotropy.
Increased thickness at enthesis	Increased thickness of the tendon insertion into the bone (<2 mm from the cortical bone) as compared with the body of tendon, with or without blurring of the tendon margins.
Erosions	Cortical break with a step-down contour defect, seen in two perpendicular planes, at the insertion of the enthesis.
Calcifications	Hyperechoic foci, with or without acoustic shadow, detected at the enthesis (<2 mm from the cortical bone).
Enthesophytes	Enthesophyte was defined as a step-up of bony prominence, seen in two perpendicular planes at the end of the bone contour of the enthesis.
Doppler signal at insertion	Doppler signal seen at bone insertion (<2 mm from the cortical bone), different from reflecting surface artefact or nutrition vessel signal, with or without cortical irregularities, erosions, or enthesophytes.

Meta-analyses concluded that the occurrence rates of each elementary lesions were 15% for calcifications, 18% for erosions, 12% for enthesophytes, 10% for edema, 16% for thickening, 12% for bursitis, and 8% for PD signal (**Supplementary Appendix 6**).

### Both gray scale ultrasound and power Doppler ultrasound are strongly recommended in the US examination of enthesitis in patients with SpA

Compared with color Doppler US (CDUS), PDUS has a much higher sensitivity because PDUS could detect small blood vessels or vessels with very slow blood flow (44). It should be noted that the vascularization of the entheses is mostly caused by small blood vessels, much to the advantage of PDUS (42). Therefore, PDUS is preferred rather than CDUS in the US examination of enthesitis.

According to an observational study in patients with SpA and healthy volunteers by D’agostino et al., enthesis vascularization was almost exclusive to patients with SpA, whereas PD signal was rarely observed at the entheses of healthy volunteers, suggesting that enthesis vascularization is an important sign in differential diagnosis (39). However, Feydy et al. and Kwiatkowska et al. held the opinion that the occurrence rates of enthesis vascularization were too low to be able to differentiate between healthy individuals and patients with SpA (45, 46). Meta-analyses showed that there was a significantly higher risk of enthesis vascularization in patients with SpA compared with controls (OR = 6.45, 95% CI [1.89, 22.04]) (**Supplementary Appendix 6**). Therefore, enthesis vascularization should be regarded as a specific sign of enthesitis in patients with SpA.

### The following entheses are conditionally recommended to be included in the US examination of enthesitis in patients with SpA: Proximal plantar fascia, distal Achilles tendon, distal and proximal patellar ligament, distal quadriceps, brachial triceps tendons, common extensor tendons, and greater trochanter

It has been reported that the occurrence rates of enthesitis at the lower limbs were higher than the upper limbs (10, 30, 39). According to the meta-analyses, occurrence rates of enthesitis at different entheses were as follows:lateral epicondyle of the elbow, 30% (95% CI [0.19, 0.43]); medial epicondyle of the elbow, 7% (95% CI [0.02, 0.21]); greater trochanter, 30% (95% CI [0.16, 0.48]); quadriceps tendons, 38% (95% CI [0.27, 0.50]); patellar ligament, 42% (95% CI [0.25, 0.62]); Achilles tendon, 39% (95% CI [0.24, 0.58]); and plantar fascia, 21% (95% CI [0.08, 0.45]) (**Supplementary Appendix 6**). Among them, proximal plantar fascia, distal Achilles tendon, distal and proximal patellar ligament, distal quadriceps, brachial triceps tendons, common extensor tendons, and greater trochanter were selected as the common sites of enthesitis, which should be included in the US examination of enthesitis in patients with SpA. It should be noted that it could be difficult to assess a clear Doppler signal at deeper enthesis such as the greater trochanter, with a certain risk of observing enthesopathic alterations.

The following positions should be taken during the US assessment:

Elbow entheses: Elbow flexed at 30°–45°.

Knee entheses: Patient lying in the supine position with the knee flexed at 30°.

Heel entheses: Patient lying prone with the feet hanging over the edge of the bed in a neutral position.

### Madrid sonography enthesitis index is conditionally recommended in the US examination of enthesitis in patients with SpA

A number of scoring or grading systems have been developed aiming at the assessment of enthesitis. Common scoring or grading systems of enthesitis include Glasgow Ultrasound Enthesitis Scoring System (GUESS) (36), Sonographic Entheseal Index (SEI) (47), Madrid sonography enthesitis index (MASEI) (41), and D’Agostino Scoring System (39) (**Table 4**). Among them, GUESS evaluates five pairs of entheses at the lower limbs with gray scale ultrasound (GSUS) (36). SEI classified signs of acute injury and chronic lesion, respectively, at five pairs of entheses at the lower limbs with GSUS (47). MASEI scores enthesitis at proximal plantar fascia, distal Achilles tendon, distal and proximal patellar ligament, distal quadriceps, and brachial triceps tendons with GSUS and PDUS (41). D’Agostino Scoring System evaluates enthesitis based on presence or absence of enthesis vascularization, acute injury, and chronic lesions (39).

**Table 4:** Overview of common scoring or grading systems of enthesitis with ultrasound examinations in patients with SpA.

System	Sites Assessed	Scoring/Grading	Pros	Cons	Reference
Glasgow Ultrasound Enthesitis Scoring System (GUESS)	Superior pole of the patella (quadriceps tendon enthesis), Inferior pole of the patella (proximal patellar ligament enthesis), Tibial tuberosity (distal patellar ligament enthesis), Superior pole of the calcaneus (Achilles tendon enthesis), and Inferior pole of the calcaneus (plantar aponeurosis enthesis)	Thickness, bursitis, erosion, and enthesophyte. Each item scores one point. Total possible score on both lower limbs is 36.	Λ Fast Λ Simple	Λ Omits entheses of the upper limbs Λ Does not evaluate vascularization	Balint et al., *Ann Rheum Dis*, 2002. 61(10): 905–910
Sonographic Entheseal Index (SEI)	Superior pole of the patella (Quadriceps tendon enthesis), Inferior pole of the patella (Proximal insertion of the patellar tendon), Anterior tibial tuberosity (Distal insertion of the patellar tendon), Superior pole of the calcaneous (Achilles tendon enthesis), and Plantar pole of the calcaneous (Plantar aponeurosis enthesis)	Signs of acute injury: Thickening of tendon/aponeurosis, Hypoechogenicity of tendon/aponeurosis, Peritendinous/periaponeurotic oedema, Bursitis. Signs of chronic lesion: Tendon tear, Loss of thickness, Tendon calcification, Bone erosion. Each variable is scored as 0 (absence) or 1 (presence) and the maximum SEI scoring is 76 points	Λ Fast Λ Simple	Λ Omits entheses of the upper limbs Λ Does not evaluate vascularization	Alcalde et al. Ann Rheum Dis, 2007. 66(8): 1015-19
Madrid sonography enthesitis index (MASEI)	Inferior pole of the calcaneus (plantar aponeurosis enthesis), Superior pole of the calcaneus (Achilles tendon enthesis), Tibial tuberosity (distal patellar ligament enthesis), Inferior pole of the patella (proximal patellar ligament enthesis), Superior pole of the patella (quadriceps tendon enthesis), and Olecranon tuberosity (triceps tendon enthesis)	Each item scores one point, except for calcification (0, 1, 2, or 3) and erosion and Doppler signal (0 or 3). The total possible score on both sides (12 entheses) is 136.	Λ Fast Λ Simple Λ Includes PDUS evaluation Λ Includes upper extremity enthesitis evaluation Λ Widely used	Λ 0–3 scoring system could contribute to greater inter- and intra-rater inconsistency	de Miguel et al., *Ann Rheum Dis*, 2009. 68(2): 169–174
D’Agostino Scoring System		Stage 1: Vascularization at the cortical junction without abnormal findings in B mode. Stage 2a: Vascularization associated with swelling and/or decreased echogenicity at the cortical junction in B mode. Stage 3a: Same as stage 2a, plus erosions of cortical bone and/or calcification of enthesis, and optional surrounding bursitis. Stage 2b: Abnormal findings in B mode as in stage 2a, but without vascularization. Stage 3b: Abnormal findings in B mode as in stage 3a, but without vascularization.	Λ Could be applied to any enthesis Λ Includes PDUS evaluation	Λ Lack of quantification Λ Vascularization could be scarce	D’Agostinon et al., *Arthritis Rheum*, 2003. 48(2): 523–533

One study comparing different scoring or grading systems of enthesitis exhibited that there was no significant difference in the sensitivity and specificity between different systems (48). However, it has been perceived that MASEI could be superior given its the advantage of PDUS evaluation as well as the inclusion of entheses at the upper limbs (41, 48). This guideline conditionally recommended MASEI in the US assessment of enthesitis in patients with SpA, but other scoring systems are also viable options (**Table 5**).

**Table 5:** Madrid Sonographic Enthesis Index (MASEI) (Ref. de Miguel et al., *Ann Rheum Dis*, 2009., 68(2): 169–74).

Data	Value
*Inferior pole of the calcaneus: plantar aponeurosis enthesis*	
Plantar aponeurosis structure	(0 or 1)
Plantar aponeurosis thickness > 4.4 mm	(0 or 1)
Inferior pole of calcaneus erosion	(0 or 3)
Inferior pole of calcaneus enthesis calcification	(0, 1, 2, or 3)
Plantar aponeurosis enthesis power Doppler	(0 or 3)
*Superior pole of the calcaneus: Achilles tendon enthesis*	
Achilles tendon structure	(0 or 1)
Achilles tendon thickness > 5.29 mm	(0 or 1)
Retrocalcaneal bursitis	(0 or 1)
Posterior pole of calcaneus erosion	(0 or 3)
Posterior pole of calcaneus enthesis calcification	(0, 1, 2, or 3)
Posterior pole of calcaneus power Doppler	(0 or 3)
*Tibial tuberosity: distal patellar ligament enthesis*	
Patellar ligament structure	(0 or 1)
Patellar ligament thickness > 4 mm	(0 or 1)
Infrapatellar bursitis	(0 or 1)
Tibial tuberosity erosion	(0 or 3)
Tibial tuberosity enthesis calcification	(0, 1, 2, or 3)
Tibial tuberosity enthesis power Doppler	(0 or 3)
*Inferior pole of the patella: proximal patellar ligament enthesis*	
Patellar ligament structure	(0 or 1)
Patellar ligament thickness > 4 mm	(0 or 1)
Inferior pole of patella erosion	(0 or 3)
Inferior pole of patella enthesis calcification	(0, 1, 2, or 3)
Inferior pole of patella enthesis power Doppler	(0 or 3)
*Superior pole of the patella: quadriceps tendon enthesis*	
Quadriceps tendon structure	(0 or 1)
Quadriceps tendon thickness > 6.1 mm	(0 or 1)
Superior pole of patella erosion	(0 or 3)
Superior pole of patella enthesis calcification	(0, 1, 2, or 3)
Superior pole of patella enthesis power Doppler	(0 or 3)
*Olecranon tuberosity: triceps tendon enthesis*	
Triceps tendon structure	(0 or 1)
Triceps tendon thickness > 4.3 mm	(0 or 1)
Olecranon erosion	(0 or 3)
Olecranon enthesis calcification	(0, 1, 2, or 3)
Olecranon enthesis power Doppler	(0 or 3)

Each item scores one point, except for calcification (0, 1, 2, or 3) and erosion and Doppler signal (0 or 3). The total possible score on both sides (12 entheses) is 136.

### US is conditionally recommended to monitor therapeutic responses of enthesitis in patients with SpA

Multiple clinical trials employing US as the assessment tool of enthesitis revealed that, after the medication of biologic DMARDs or conventional synthetic DMARDs, US scores decreased significantly (27, 30, 38, 40, 49–51). Aydin et al. and Wang et al. demonstrated that, after medication of tumor necrosis factor-α (TNF-α) inhibitors in patients with ankylosing spondylitis, both GSUS scores and total US scores decreased significantly, whereas there was no significant difference between different TNF-α inhibitors (49, 51). A study by Hartung et al. showed that PDUS scores were significantly lowered after patients with SpA were treated with csDMARDs and bDMARDs (27). Another study by Seven et al. demonstrated that enthesitis was not significantly improved by treatment, which could be attributed to the fact that this study mainly evaluated chronic lesions (30).

Most studies could not identify an association between disease activity indicators and US scores, suggesting that enthesitis might be an indicator independent from systemic inflammation (17, 40, 48, 51, 52).

Overall, US is a reliable tool in the assessment of therapeutic responses of enthesitis in patients with SpA. This guideline endorsed the application of US in the context of both clinical trials and clinical practice.

### MRI is conditionally recommended in the assessment of enthesitis in patients with SpA

MRI is a reliable tool in the evaluation of enthesitis, capable of providing high-resolution evidence of tissue abnormalities at the entheses (23, 53, 54). High spatial resolution of MRI could help clinicians differentiate enthesitis from other conditions causing regional pain (15). Apart from the imaging of the inflammatory changes at the soft tissue adjacent to the entheses, MRI is currently the only modality able to present osteitis at the insertion sites (54, 55). Numerous studies have substantiated that sensitivity of MRI in the detection of enthesitis could parallel US examination, and the agreement between MRI and US was satisfactory (23, 56, 57). However, several disadvantages have limited the wide application of MRI in clinical scenarios. Compared with US, MRI is more costly and less available. Conventional MRI sequences could only image entheses at a specific location, incapable of getting the bigger picture, with the exception of whole-body MRI (58, 59). Moreover, the enthesis is mostly composed of tightly packed collagen fibers with little water accumulation, resulting in difficulties in imaging the entheses with conventional fat-saturated water-sensitive MRI sequences (60). With the recent developments of MRI imaging techniques, whole-body MRI and novel sequences such as UTE have shown great promise in the detection of enthesitis (59, 60). Therefore, this guideline conditionally recommended MRI examinations in patients with SpA whose US examinations are inconclusive.

### Whole-body MRI is conditionally recommended against as the screening method for enthesitis in patients with SpA

Conventional MRI sequences could only image entheses at a specific location, whereas whole-body MRI could present inflammation at the axial skeleton as well as peripheral entheses (58, 59). By placing multiple coils throughout the body, whole-body MRI could complete scanning from head to toe in one single examination, without the need of repositioning (61, 62). Research showed that the overall readability of whole-body MRI was satisfactory, whereas the agreement between different observers was good (63, 64). OMERACT MRI group standardized the image acquisition and the scanning plane for evaluations in whole-body MRI in 2017 (65). MRI-Whole-Body Score for Inflammation in Peripheral Joints and Entheses in Inflammatory Arthritis (MRI-WIPE) scoring system was devised by Krabbe et al. for the assessment of peripheral joints and entheses in whole-body MRI (64). Credibility of whole-body MRI has been validated and supported by a number of clinical trials (66–69). On the basis of the current evidence of whole-body MRI, common sites of enthesitis included anterior chest wall, pelvis and lower limbs, notably sternoclavicular joint, acromioclavicular joint, ischial tuberosity, and Achilles tendon (70, 71). However, compared with conventional sequences, spatial resolution of whole-body MRI is much lower, especially at the entheses of the distal limbs, resulting in weak confidence of evaluations at these sites (70). Another weakness of whole-body MRI is that the scanning time is much longer, ranging from 40 min to 1 h, whereas reading and scoring of the whole-body MRI images take another hour or so, rendering the whole process time-consuming (63). This guideline conditionally recommended against using whole-body MRI as the routine examination for enthesitis in clinical practice. It is suggested that this modality should only be considered in clinical trials.

### Ultrashort echo time sequence is conditionally recommended in the MRI examination of enthesitis in patients with SpA

The transverse relaxation time (T2) is very short at locations such as tendons and entheses, hence the extremely low signal or no signal on images of conventional MRI sequences, especially T2 fat-saturated images (60). Only advanced enthesitis with very conspicuous edema could be observed on these images, whereas early phase of enthesitis is difficult to visualize and differentiate from normal tissue (72). The echo time of ultrashort echo time (UTE) sequence is much shorter than conventional sequences, enabling it to detect the signal emitted from the short T2 components at the entheses (72). On the basis of this rationale, the UTE spectroscopic imaging sequence, three-dimensional UTE cones sequence are developed, with quantifying measurement of Cones-T2* values and Cones-MTR, which could assist in the differentiation between early enthesitis and normal enthesis (60, 73). According to related studies, UTE sequence enables the clear visualization of entheseal structures, with display of the fibrocartilaginous components and collagenous components of the enthesis (60, 72, 74). However, availability limits the application of this sequence. This guideline conditionally recommended the use of UTE sequence in the MRI examination of enthesitis when UTE is available.

### Contrast-enhanced MRI is strongly recommended against in the MRI examination of enthesitis in patients with SpA

There is limited research on the diagnostic accuracy of contrast-enhanced MRI in the imaging of enthesitis. A few studies demonstrated that contrast-enhanced MRI could identify relatively more sites of enthesitis at lumbar vertebra and pelvis, yet the identification of enthesitis did not provide incremental diagnostic values to bone marrow edema (75, 76). At the peripheral entheses, the administration of contrast agents could help identify only a small number of extra enthesitic lesions, approximately 10% more (62). Conversely, injection of contrast agents exposed patients to threats of impaired renal function and allergies to contrast agents (77). In this case, benefits of contrast-enhanced MRI are outweighed by the potential risks, because contrast-enhanced MRI does not provide much additional information. Therefore, this guideline strongly recommended against contrast-enhanced MRI in the detection of enthesitis.

### The OMERACT Heel Enthesitis MRI Scoring System is conditionally recommended in the evaluation of heel enthesitis in patients with SpA

The Achilles tendon and plantar fascia are one of the most frequent sites of enthesitis in patients with SpA. By using the heel as the prototype, the OMERACT MRI group put forward the Heel Enthesitis MRI Scoring System (HEMRIS), which categorized the pathologies of enthesitis on MRI into inflammatory lesions and structural lesions (**Table 6**) (78, 79). This scoring system has been validated in patients with enthesitis at the Achilles tendon and plantar fascia and proved to be reliable (80). The inflammatory parameters evaluated intra-tendon hypersignal on T2w/short-tau inversion recovery (STIR) sequences, peri-tendon hypersignal, bone marrow edema, and bursitis, whereas structural lesions were defined as enthesophyte, bone erosion, and tendon thickening. STIR or T2 fat-saturated sequence is recommended in the assessment of inflammatory pathologies. T1-weighted images are recommended when evaluating structural lesions. However, no prospective study has employed this scoring system as the trial endpoints, so its capacity of monitoring disease modification or therapeutic responses was still inconclusive. HEMRIS is currently applied in the evaluation of heel, but its potential in evaluating other entheses awaits further exploring.

**Table 6:** Heel Enthesitis Scoring System (HEMRIS) (Ref. Mathew et al., *J Rheumatol*, 2019., 46(9): 1232–1238).

Pathology	Definition
1. Intra-tendon hypersignal (entheseal tendonitis)	Signal characteristics consistent with increased water content/inflammation* within the tendon/ligament/aponeurosis close to its insertion
2. Peri-tendon hypersignal (entheseal peritendinitis)	Signal characteristics consistent with increased water content/inflammation * in the soft tissues surrounding the tendon/ligament/aponeurosis, close to its insertion
3. Bone marrow edema (entheseal osteitis)	Bone lesion with ill-defined margins and signal characteristics consistent with increased water content/inflammation*, close to the tendon/aponeurosis insertion
4. Bursitis†	Signal characteristics consistent with increased water content/inflammation* in an above-normal sized bursa
5. Tendon/aponeurosis thickening	Abnormal thickening of the tendon/aponeurosis close to its insertion
6. Enthesophyte	Abnormal bone formation at the insertion of tendon/ligament/aponeurosis insertion into the bone
7. Bone erosion (entheseal bone erosion)	A sharply marginated bone lesion, with typical signal characteristics** and a visible cortical break, located close to the tendon/ligament/aponeurosis insertion
8. Intra-tendon hypersignal on T1w	Increased signal in T1-weighted sequence within the tendon/ligament/aponeurosis close to its insertion

^†^This lesion should only be assessed in entheseal regions in which a relevantly located bursa is present.

*High signal intensity on short-tau inversion recovery/T2wFS images and/or above normal post-gadolinium enhancement on T1W images.

**On T1W images without contrast injection: loss of normal low signal intensity of cortical bone and loss of normal high signal intensity of marrow fat. T2wFS, T2w fat-suppressed (images).

### Radiograph is conditionally recommended against in the assessment of enthesitis in patients with SpA

Radiograph is a viable tool in the detection of enthesitis, but it could only present chronic structural changes such as bone erosions and enthesophytes. Unlike US or MRI, it could not provide information of inflammatory lesions in the acute phase, such as edema and thickening at the enthesis or osteitis. On the other hand, the sensitivity of radiographs in the detection of bone erosions and enthesophytes was relatively low. Radiographs could not compete with US or MRI even in the detection of bone structures and enthesophytes. Only in patients with long-standing enthesitis who present conspicuous cortical bone changes or enthesophytes, radiograph is capable of imaging such changes. Considering that radiograph could provide only limited information about enthesitis, which can rarely instruct therapeutic decision-making but exposes patients to certain amount of radiation, radiograph is conditionally recommended against in this guideline.

### PET/CT is strongly recommended against in the assessment of enthesitis in patients with SpA

It was hypothesized that, because the regional accumulation of 18F-FDG could reflect certain extent of tissue inflammation status, PET/CT could potentially be a promising tool in diagnosing enthesitis (80). However, studies showed that enthesitis as observed on MRI images was not correlated with the accumulation of 18F-FDG (80). Only the structural damage of the Achilles tendon was weakly correlated with the metabolic activity on PET/CT. On the basis of the current evidence, we do not consider PET/CT a reliable tool in the detection of enthesitis in patients with SpA. This is probably due to the relatively low vascularization of the enthesis, which fails to deliver the tracers to the enthesis. Considering that not only PET/CT is expensive and hardly available, but also the tracers of this examination expose patients to unwarranted radiation (81), this guideline strongly recommended against using PET/CT as the routine approach to the diagnosis of enthesitis in clinical practice.

## Author contributions

JG, KY, and JR belonged on the core team and were in charge of the overall development of this guideline. XW, DL, YL, YX, LT, Yanli Zhang, XZ, LF, XL, Zhiming Lin, ZetL, and BL conducted the systematic literature review. LR, YuqZ, NY, JX, HZ, BX, ZW, FZ, ZhiL, WX, SL, YiZ, SY, QL, LJZ, DZ, SH, LKZ, LW, HL, DG, YunZ, and ZY served on the voting panel. All authors were involved in drafting the article or revising it critically for important intellectual content, and all authors approved the final version to be submitted for publication. Dr. Gu had full access to all of the data in the study and takes responsibility for the integrity of the data and the accuracy of the data analysis.

## Funding

This work was supported by the grants from the National Natural Science Foundation of China (81871294); the Science and Technology Planning Project of Guangdong Province, China (2019B030316004); Guangdong Clinical Research Center of Immune diseases (2020B1111170008).

## Acknowledgments

We thank Joachim Sieper, David Yu, Buyun Yu, Xiaofeng Zeng, and James Zheng for providing counsel for this guideline. We thank Guangdong Provincial Clinical Studies Center for Immunological Diseases in organizing the online meetings and undertaking the administrative work of this project.

## Conflict of interest

The authors declare that the research was conducted in the absence of any commercial or financial relationships that could be construed as a potential conflict of interest.

## Publisher’s note

All claims expressed in this article are solely those of the authors and do not necessarily represent those of their affiliated organizations, or those of the publisher, the editors and the reviewers. Any product that may be evaluated in this article, or claim that may be made by its manufacturer, is not guaranteed or endorsed by the publisher.
