# Supplementary material for: A clinical practice guideline for the screening and assessment of enthesitis in patients with spondyloarthritis
Source: Front Immunol. 2022 Sep 12;13:978504. doi: 10.3389/fimmu.2022.978504 (PMC9510351; doi:10.3389/fimmu.2022.978504)
Supplement: Supplementary file 1 [file DataSheet_1.docx]

Supplementary appendix 1: methods

Methodology overview

This guideline was developed following the Grading of Recommendations Assessment, Development, and Evaluation (GRADE) methodology (www.gradeworkinggroup.org).[1-3] Since this guideline is targeted at non-interventional approaches to the screening of enthesitis in the population of SpA patients, the GRADE methodology was modified to suit its nature.

Teams involved

The core team was comprised of experts in the field of rheumatology and methodology, including one GRADE methodologist who provided counsel on the modification of the GRADE methodology in non-interventional approaches, as well as on the process of evidence gathering and evaluation. The core team (5 members) was in charge of this project and was tasked with defining the scope, drafting the basic structure of this guideline, devising clinical questions, handing out assignments to the literature review team, supervising the voting process and the drafting of this guideline.

The literature review team (5 members) received assignments from the core team and conducted systemic literature review on the topics concerned. Team members were required to search the databases for pertinent papers, assessed the quality of the studies, extracted the data, computed pooled estimates, graded the quality of evidence, generated the summary of findings table and drafted the evidence reports.

The voting panel was comprised of individuals with the expertise in rheumatology, radiology, ultrasonography and orthopedics. The voting panel was invited to participate in defining the scope and devising the clinical questions. After evidence reports were generated, evidence reports were present to the voting panel. Having examined the evidence reports, the voting panel was invited to vote on each clinical question.

All members participating in the development of this guideline received training sessions regarding the GRADE methodology and its modification. Rosters of each team could be seen in Supplementary Appendix 2.

Conflict of interests

Principal investigators of this guideline had no relevant conflicts of interest before the initiation of this project, while the majority of the team members in the development of this guideline declared no conflicts of interest throughout the period. Individuals employed by companies that manufactured or sold diagnostics or therapeutics were deemed ineligible to participate. In the meanwhile, individuals who had any relationship with such companies were considered conflicted. Intellectual conflicts, defined as previous publication or projects pertinent to the diagnosis of enthesitis were exempt, under the condition that such conflicts were fully disclosed.

Scope and target audience

The scope of this guideline was mainly approaches to the screening and evaluation of enthesitis in the population of SpA patients. Enthesitis related to mechanical injuries was not included in the scope of this guideline. The target audience for this guideline includes physicians and radiologists who might come in contact with patients with SpA.

Development of the framework and clinical questions

The core team led a joint effort with the voting panel and the literature review team to devise the framework and the initial set of clinical questions for this guideline. Since this guideline mainly cover non-interventional approaches to the screening and evaluation of enthesitis, the PICO questions do not fit in our framework. Instead, the clinical questions were mostly concerned about the clinical information these diagnostic approaches were able to present, their capacity in assisting in diagnosis and monitoring therapeutic responses, their expenses and accessibility as well as the potential risks.

The core team held weekly meetings with the literature review team to discuss the developments of this guideline. A conference was convened among all the members of the core team, the voting panel and the literature review team to determine the framework of this guideline. It was agreed upon that this guideline should address the clinical significance of the following approaches to the screening and evaluation of enthesitis: history taking, physical examination, ultrasonography, MRI, radiographs and PET/CT. Once the basic framework was determined, clinical questions were further devised to specify the significance and technical details of each approach. Additional questions were discussed by email after this meeting.

Systemic review of the literature

Literature searches

In order to gather all the articles pertinent to the clinical questions, each member of the literature review team performed systemic literature searches based on the assignments each member received. All the articles were searched in the databases including Embase, Pubmed, Web of science and Cochrane Library. Details of the search strategies could be seen in Supplementary Appendix 3.

Study selection

Having retrieved articles identified as potentially eligible with full text, two independent members of the literature review team screened through these articles to rule out duplicates. Another two independent reviewers went through all the eligible articles and matched each article to the clinical questions. All the manuscripts were subject to evaluation of study quality, employing the Newcastle-Ottawa Quality Assessment Scale.[4] In circumstances where the studies investigated enthesitis in patients with conditions other than SpA, such as psoriatic arthritis, such studies were also included, but the quality of evidence was rated down for indirectness.

Data extraction and analysis

Members of the literature review team extracted data from the included studies, when applicable. The R platform (The R Project for Statistical Computing, Vienna, Austria), version 3.6.3 was employed to calculate the pooled estimates of the statistics, using the R package “meta”. All the pooled estimates were reported with 95% confidence intervals. The majority of the included studies were observational studies, and data pooling was conducted mostly on epidemiological data and discriminative power of the evaluation approaches.

Evidence report drafting

Two independent reviewers were tasked with evaluating the quality of evidence for each clinical question, by means of the GRADE quality assessment criteria.[1] Once disagreements occurred between the two reviewers, the rest of the literature review team reviewed the evidence and settle the disagreements. The summary of findings tables were presented in the evidence reports listed in Supplementary Appendix 3. After the compilation, the evidence reports were submitted to the core team for further evaluation. One member of the literature review team collected criticisms from the core team, and revised the evidence reports.

Moving from evidence to recommendations

Since this guideline mainly addressed the non-interventional approaches to the screening and evaluation of enthesitis, the GRADE methodology was not fully applicable to this guideline. Instead, certain modifications were applied to the GRADE methodology under the advice of Professor Kehu Yang. A recommendation should be made based on the inclusive consideration of cost, accessibility and the clinical significance of the evaluation approaches, together with the quality of evidence. The overarching principle is that a recommendation should only be made when this approach can provide clinical information which can assist in diagnosis and monitoring of therapeutic responses, with relatively low costs and easy accessibility.

Based on the costs and accessibility, all the approaches were categorized as 1) inexpensive and easily accessible, 2) moderately costly and relatively accessible; 3) expensive and difficult to access. The strengths of each recommendation were classified as strong or conditional. A strong recommendation was given upon the consideration that the approach of screening and examining enthesitis could provide critical information that could educate and modify disease management options with relatively low costs and high accessibility. A conditional recommendation was given when moderate information could be gained with corresponding costs and accessibility. Where there was low evidence supporting the application of approaches that are inexpensive and easily accessible, a strong recommendation was given. For approaches that were moderately costly and relatively accessible, moderate certainty of evidence warranting its necessity was deemed sufficient to support a strong recommendation. For approaches that were expensive and difficult to access, it takes high certainty of evidence to make a recommendation.

Consensus building

Once all the evidence reports had been compiled, the evidence reports were sent to the voting panel through email. After careful examination of the evidence reports, each member of the voting panel received a ballot through email and was invited to fill out the ballot, regarding the direction and strength of each recommendation. One member of the literature review team collected the ballots and calculated the approval ratings and strengths of each recommendation. Only one round of voting was held, and a 70% consensus was regarded necessary for a recommendation to be included in the guideline; if the 70% threshold was not fulfilled, this recommendation was discarded from the guideline.

As stated above, the voting panel could make a strong recommendation even though evidence quality was low. This type of recommendation was usually found in approaches that were inexpensive and easily accessible.

Reference:

1. Guyatt G.H., Oxman A.D., Vist G.E., Kunz R., Falck-Ytter Y., Alonso-Coello P., et al. GRADE: an emerging consensus on rating quality of evidence and strength of recommendations*.* *Bmj* 2008;**336**(7650):924-6 doi: 10.1136/bmj.39489.470347.AD.

2. Andrews J., Guyatt G., Oxman A.D., Alderson P., Dahm P., Falck-Ytter Y., et al. GRADE guidelines: 14. Going from evidence to recommendations: the significance and presentation of recommendations*.* *J Clin Epidemiol* 2013;**66**(7):719-25 doi: 10.1016/j.jclinepi.2012.03.013.

3. Andrews J.C., Schünemann H.J., Oxman A.D., Pottie K., Meerpohl J.J., Coello P.A., et al. GRADE guidelines: 15. Going from evidence to recommendation-determinants of a recommendation's direction and strength*.* *J Clin Epidemiol* 2013;**66**(7):726-35 doi: 10.1016/j.jclinepi.2013.02.003.

4. Wells GA, Shea B, O’Connell D, Peterson J, Welch V, Losos M, et al. The Newcastle-Ottawa Scale (NOS) for assessing the quality of nonrandomized studies in meta-analyses. Available at: http://www.ohri.ca/programs/clinical_epidemiology/oxford.htm. Accessed March 20, 2022.
