# Supplementary material for: A clinical practice guideline for the screening and assessment of enthesitis in patients with spondyloarthritis
Source: Front Immunol. 2022 Sep 12;13:978504. doi: 10.3389/fimmu.2022.978504 (PMC9510351; doi:10.3389/fimmu.2022.978504)
Supplement: Supplementary file 2 [file DataSheet_2.docx]

**SUPPLEMENTARY APPENDIX 2: Panel/Teams Involved**

**Core Leadership Team**

Jieruo Gu, MD, PhD (the Third Affiliated Hospital of Sun Yat-Sen University, Guangzhou, China; Project Co-PI), Jun Shen, MD, PhD (Sun Yat-Sen Memorial Hospital of Sun Yat-Sen University, Guangzhou, China, Project Co-PI), Jie Ren, MD, PhD (the Third Affiliated Hospital of Sun Yat-Sen University, Guangzhou, China; Project Co-PI), Kehu Yang, MD, PhD (Lanzhou University, Lanzhou, China; Project Co-PI)

**Voting Panel**

Limin Rong (the Third Affiliated Hospital of Sun Yat-Sen University, Guangzhou, China), Yuqi Zhou (the Third Affiliated Hospital of Sun Yat-Sen University, Guangzhou, China), Niansheng Yang (the First Affiliated Hospital of Sun Yat-Sen University, Guangzhou, China), Jian Xu (the First Affiliated Hospital of Kunming Medical University, Kunming, China), Hua Zhang (the Fifth Affiliated Hospital of Sun Yat-Sen University, Guangzhou, China), Baijie Xu (Jieyang City People’s Hospital, Jieyang, China), Zhenbiao Wu (Xijing Hospital, Xi’an, China), Feng Zhan (Hainan General Hospital, Haikou, China), Zhenbin Li (Bethune International Peace Hospital, Shijiazhuang, China), Weiguo Xiao (the First Hosptial of China Medical University, Shenyang, China), Shengyun Liu (the first Affiliated Hospital of Zhengzhou University, Zhengzhou, China), Yi Zhou (the First Affiliated Hospital of Jinan University, Guangzhou, China), Shanhui Ye (the First Affiliated Hospital of Guangzhou Medical University, Guangzhou, China), Qing Lyu (the Third Affiliated Hospital of Sun Yat-Sen University, Guangzhou, China), Lijun Zhang (the University of Hong Kong-Shenzhen Hosptital, Shenzhen, China), Dongbao Zhao (Changhai Hospital, Shanghai, China), Shanzhi He (Zhongshan City People’s Hospital, Zhongshan, China), Like Zhao (Beijing Hospital, Beijing, China), Lijun Wu (People’s Hospital of Xinjiang Uygur Autonomous Region, Urumchi, China), He Lin (Fujian Provincial Hospital, Fuzhou, China), Yunxiao Zhu (the Seventh Affiliated Hospital of Sun Yat-Sen University, Shenzhen, China), Donggeng Guo (People’s Hospital of Ningxia Hui Autonomous Region, Yinchuan, China), Zehong Yang (Sun Yat-Sen Memorial Hospital of Sun Yat-Sen University, Guangzhou, China)

**Literature Review Team**

Xinyu Wu (the Third Affiliated Hospital of Sun Yat-Sen University, Guangzhou, China), Dong Liu (the Third Affiliated Hospital of Sun Yat-Sen University, Guangzhou, China), Wang (Lanzhou University, Lanzhou, China), Ya Xie (the Third Affiliated Hospital of Sun Yat-Sen University, Guangzhou, China), Liudan Tu (the Third Affiliated Hospital of Sun Yat-Sen University, Guangzhou, China), Yanli Zhang (the Third Affiliated Hospital of Sun Yat-Sen University, Guangzhou, China), Xi Zhang (the Third Affiliated Hospital of Sun Yat-Sen University, Guangzhou, China), Linkai Fang (the Third Affiliated Hospital of Sun Yat-Sen University, Guangzhou, China), Xiqing Luo (the Third Affiliated Hospital of Sun Yat-Sen University, Guangzhou, China), Zhiming Lin (the Third Affiliated Hospital of Sun Yat-Sen University, Guangzhou, China), Zetao Liao (the Third Affiliated Hospital of Sun Yat-Sen University, Guangzhou, China), Budian Liu (the Third Affiliated Hospital of Sun Yat-Sen University, Guangzhou, China)
