# Supplementary material for: A clinical practice guideline for the screening and assessment of enthesitis in patients with spondyloarthritis
Source: Front Immunol. 2022 Sep 12;13:978504. doi: 10.3389/fimmu.2022.978504 (PMC9510351; doi:10.3389/fimmu.2022.978504)
Supplement: Supplementary file 3 [file DataSheet_3.docx]

**Search Strategies**

| **Syntax Guide for PubMed** |
| --- |
| MeSH = Medical Subject Heading |
| All Fields = Word appears in title, abstract, keyword, author, editor, publisher, journal, etc. |
| **Additional Syntax Guide for Embase** |
| Exp = A command to retrieve all narrower subject headings (MeSH) |
| **Additional Syntax Guide for Cochrane Library** |
| ti,ab,kw = Word appears in title, abstract or keyword field of record; word variations are searched in title, abstract, keyword search |
| **Boolean Operators** |
| AND = retrieves results that include all the search terms |
| OR = retrieves results that include at least one of the search terms |
| NOT = excludes the retrieval of terms from the search |

**Database: PubMed**

Search strategies:

| #1 | "spondylarthritis"[MeSH Terms] OR "spondylarthritis"[All Fields] OR "spondyloarthritis"[All Fields] |  |
| --- | --- | --- |
| #2 | "spondylitis, ankylosing"[MeSH Terms] OR ("spondylitis"[All Fields] AND "ankylosing"[All Fields]) OR "ankylosing spondylitis"[All Fields] OR ("ankylosing"[All Fields] AND "spondylitis"[All Fields]) |  |
| #3 | #1 OR #2 |  |
| #4 | #3 AND ("enthesal"[All Fields] OR "entheseal"[All Fields] OR "entheses"[All Fields] OR "enthesitis"[All Fields]) |  |
| #5 | #4 AND 1946/01/01:2021/06/01[Date - Publication] |  |
| #6 | #5 AND "english"[Language] |  |
| #7 | #6 AND ("diagnosable"[All Fields] OR "diagnosi"[All Fields] OR "diagnosis"[MeSH Terms] OR "diagnosis"[All Fields] OR "diagnose"[All Fields] OR "diagnosed"[All Fields] OR "diagnoses"[All Fields] OR "diagnosing"[All Fields] OR "diagnosis"[Subheading]) |  |
| #8 | #6 AND ("quality of life"[MeSH Terms] OR ("quality"[All Fields] AND "life"[All Fields]) OR "quality of life"[All Fields] OR ("life"[All Fields] AND "quality"[All Fields]) OR "life quality"[All Fields]) |  |
| #9 | #6 AND ("cost of illness"[MeSH Terms] OR ("cost"[All Fields] AND "illness"[All Fields]) OR "cost of illness"[All Fields] OR ("disease"[All Fields] AND "burden"[All Fields]) OR "disease burden"[All Fields]) |  |
| #10 | #8 OR #9 |  |
| #11 | #6 AND ("prognosis"[MeSH Terms] OR "prognosis"[All Fields] OR "prognoses"[All Fields]) |  |
| #12 | #6 AND ("disease progression"[MeSH Terms] OR ("disease"[All Fields] AND "progression"[All Fields]) OR "disease progression"[All Fields]) |  |
| #13 | #11 OR #12 |  |
| #14 | #7 OR #10 OR #13 | PART 1:  OVERVIEW  (Q1-Q3)  1142 results |
| #15 | #6 AND ("history"[MeSH Terms] OR "history"[All Fields] OR "histories"[All Fields] OR "history"[Subheading]) |  |
| #16 | #6 AND ("symptoms"[All Fields] OR "symptom"[All Fields] OR "symptom's"[All Fields] OR "symptomes"[All Fields]) |  |
| #17 | #15 OR #16 | PART 2:  HISTORY TAKING (Q4)  353 results |
| #18 | #6 AND ("physical examination"[MeSH Terms] OR ("physical"[All Fields] AND "examination"[All Fields]) OR "physical examination"[All Fields] OR ("clinical"[All Fields] AND "examination"[All Fields]) OR "clinical examination"[All Fields]) |  |
| #19 | #6 AND ("Mander"[All Fields] AND "enthesitis index"[Title/Abstract]) |  |
| #20 | #6 AND "maastricht ankylosing spondylitis enthesitis score"[Title/Abstract] |  |
| #21 | #6 AND ("SPARCC"[All Fields] AND "enthesitis index"[Title/Abstract]) |  |
| #22 | #6 AND "leeds enthesitis index"[Title/Abstract] |  |
| #23 | #6 AND ("Gladman"[All Fields] AND "Index"[Title/Abstract]) |  |
| #24 | #6 AND "major index"[Title/Abstract] |  |
| #25 | #6 AND "berlin index"[Title/Abstract] |  |
| #26 | #19 OR #20 OR #21 OR #22 OR #23 OR #24 OR #25 | PART 3:  PHYSICAL EXAMINATION (Q5-Q8)  266 results |
| #27 | #6 AND ("diagnostic imaging"[Subheading] OR ("diagnostic"[All Fields] AND "imaging"[All Fields]) OR "diagnostic imaging"[All Fields] OR "ultrasound"[All Fields] OR "ultrasonography"[MeSH Terms] OR "ultrasonography"[All Fields] OR "ultrasonics"[MeSH Terms] OR "ultrasonics"[All Fields] OR "ultrasounds"[All Fields] OR "ultrasound's"[All Fields]) | PART 4：  ULTRASOUND  (Q9-Q17)  481 results |
| #28 | #6 AND ("magnetic resonance imaging"[MeSH Terms] OR "magnetic resonance imaging"[All Fields] OR "mri"[All Fields]) | PART 5：  MRI  (Q18-Q22)  283 results |
| #29 | #6 AND ("radiology"[MeSH Terms] OR "radiology"[All Fields] OR "radiography"[MeSH Terms] OR "radiography"[All Fields] OR "radiology's"[All Fields]) |  |
| #30 | #6 AND ("x ray"[All Fields] OR "x-rays"[MeSH Terms] OR "x-rays"[All Fields]) |  |
| #31 | #29 OR #30 | PART 6:  X-RAY (Q23)  277 results |
| #32 | #6 AND ("positron emission tomography computed tomography"[MeSH Terms] OR "positron emission tomography"[All Fields] OR "positron emission tomography computed tomography"[All Fields] OR ("pet"[All Fields] AND "ct"[All Fields]) OR "pet ct"[All Fields]) | PART 7:  PET-CT (Q24)  15 results |

**Database: Embase**

Search strategies:

| #1 | 'spondylarthritis'/exp OR spondylarthritis |  |
| --- | --- | --- |
| #2 | 'ankylosing spondylitis'/exp OR 'ankylosing spondylitis' |  |
| #3 | #1 OR #2 |  |
| #4 | 'enthesitis'/exp OR 'enthesitis' |  |
| #5 | #3 AND #4 AND [01-01-1974]/sd NOT [02-06-2021]/sd AND [english]/lim |  |
| #6 | 'diagnosis'/exp OR 'diagnosis' |  |
| #7 | 'quality of life'/exp OR 'quality of life' |  |
| #8 | 'disease burden'/exp OR 'disease burden' |  |
| #9 | 'prognosis'/exp OR 'prognosis' |  |
| #10 | 'disease exacerbation'/exp OR 'disease exacerbation' |  |
| #11 | #6 OR #7 OR #8 OR #9 OR #10 |  |
| #12 | #5 AND #11 | PART 1:  OVERVIEW  (Q1-Q3)  2755 results |
| #13 | 'history'/exp OR 'history' |  |
| #14 | 'symptom'/exp OR 'symptom' |  |
| #15 | #13 OR #14 |  |
| #16 | #5 AND #15 | PART 2:  HISTORY TAKING (Q4)  915 results |
| #17 | 'clinical examination'/exp OR 'clinical examination' |  |
| #18 | 'physical examination'/exp OR 'physical examination' |  |
| #19 | 'mander enthesitis index' |  |
| #20 | 'maastricht ankylosing spondylitis enthesitis score' |  |
| #21 | 'sparcc enthesitis index' |  |
| #22 | 'leeds enthesitis index' |  |
| #23 | 'gladman index' |  |
| #24 | 'major index' |  |
| #25 | 'berlin index' |  |
| #26 | #17 OR #18 OR #19 OR #20 OR #21 OR #22 OR #23 OR #24 OR #25 |  |
| #27 | #5 AND #26 | PART 3:  PHYSICAL EXAMINATION (Q5-Q8)  692 results |
| #28 | 'ultrasound'/exp OR 'ultrasound' |  |
| #29 | #5 AND #28 | PART 4：  ULTRASOUND  (Q9-Q17)  545 results |
| #30 | 'nuclear magnetic resonance imaging' |  |
| #31 | #5 AND #30 | PART 5：  MRI  (Q18-Q22)  828 results |
| #32 | 'radiology'/exp OR 'radiology' |  |
| #33 | 'x ray' |  |
| #34 | #32 OR #33 |  |
| #35 | #5 AND #34 | PART 6:  X-RAY (Q23)  586 results |
| #36 | 'positron emission tomography' |  |
| #37 | #5 AND #36 | PART 7:  PET-CT (Q24)  33 results |

**Database: Cochrane Library**

Search strategies:

| #1 | ("spondylarthritis"):ti,ab,kw |  |
| --- | --- | --- |
| #2 | ("ankylosing spondylitis"):ti,ab,kw |  |
| #3 | #1 OR #2 |  |
| #4 | ("enthesitis"):ti,ab,kw |  |
| #5 | ("enthesis"):ti,ab,kw |  |
| #6 | ("enthesiopathy"):ti,ab,kw |  |
| #7 | #4 OR #5 OR #6 |  |
| #8 | #3 AND #7 with Cochrane Library publication date from Jan 1971 to May 2021 |  |
| #9 | ("diagnosis"):ti,ab,kw |  |
| #10 | ("quality of life"):ti,ab,kw |  |
| #11 | ("quality of life"):ti,ab,kw |  |
| #12 | #10 OR #11 |  |
| #13 | ("prognosis"):ti,ab,kw |  |
| #14 | ("disease progression"):ti,ab,kw |  |
| #15 | #13 OR #14 |  |
| #16 | #9 OR #12 #15 |  |
| #17 | #8 AND #16 | PART 1:  OVERVIEW  (Q1-Q3)  82 results |
| #18 | ("history"):ti,ab,kw |  |
| #19 | ("symptom"):ti,ab,kw |  |
| #20 | #18 OR #19 |  |
| #21 | #8 AND #20 | PART 2:  HISTORY TAKING (Q4)  73 results |
| #22 | ("clinical examination"):ti,ab,kw |  |
| #23 | ("physical examination"):ti,ab,kw |  |
| #24 | ("mander enthesitis index"):ti,ab,kw |  |
| #25 | ("maastricht ankylosing spondylitis enthesitis score"):ti,ab,kw |  |
| #26 | ("sparcc enthesitis index"):ti,ab,kw |  |
| #27 | ("leeds enthesitis index"):ti,ab,kw |  |
| #28 | ("gladman index"):ti,ab,kw |  |
| #29 | ("major index"):ti,ab,kw |  |
| #30 | ("berlin index"):ti,ab,kw |  |
| #31 | #22 OR #23 OR #24 OR #25 OR #26 OR #27 OR #28 OR #29 OR #30 |  |
| #32 | #8 AND #31 | PART 3:  PHYSICAL EXAMINATION (Q5-Q8)  76 results |
| #33 | ("ultrasound"):ti,ab,kw |  |
| #34 | #8 AND #33 | PART 4：  ULTRASOUND  (Q9-Q17)  13 results |
| #35 | ("magnetic resonance imaging"):ti,ab,kw |  |
| #36 | #8 AND #35 | PART 5：  MRI  (Q18-Q22)  34 results |
| #37 | ("radiology"):ti,ab,kw |  |
| #38 | ("x ray"):ti,ab,kw |  |
| #39 | #37 OR #38 |  |
| #40 | #8 AND #39 | PART 6:  X-RAY (Q23)  15 results |
| #41 | ("positron emission tomography"):ti,ab,kw |  |
| #42 | ("PET CT"):ti,ab,kw |  |
| #43 | #41 OR #42 |  |
| #44 | #8 AND #43 | PART 7:  PET-CT (Q24)  0 result |
