# Supplementary material for: A clinical practice guideline for the screening and assessment of enthesitis in patients with spondyloarthritis
Source: Front Immunol. 2022 Sep 12;13:978504. doi: 10.3389/fimmu.2022.978504 (PMC9510351; doi:10.3389/fimmu.2022.978504)
Supplement: Supplementary file 4 [file DataSheet_4.docx]

**Flowchart of the Study Selection Process**

**PART 1: OVERVIEW (Question 1-3)**

Records identified through searching of PubMed (n=1142), Embase (n=2755) and Cochrane Library (n = 82)

Total records screened after duplicates and
non-English publications removed
(n = 3362)

Total excluded after title and abstract screening (n = 3300)

*Citations did not match study designs of interest, did not examine populations or interventions of interest, or did not report outcome measures of interest*

Full-text articles assessed for eligibility
(n = 62)

Full-text articles excluded (n = 42)

*Citations did not provide evidence matching questions of interest*

Studies considered
for evidence report
(n = 20)

Studies excluded during data extraction
(n = 1)

Studies matched

to this part
(n = 19)

**PART 2: HISTORY TAKING (Question 4)**

Records identified through searching of PubMed (n=353), Embase (n=915) and Cochrane Library (n = 73)

Total records screened after duplicates and
non-English publications removed
(n = 1187)

Total excluded after title and abstract screening (n = 1161)

*Citations did not match study designs of interest, did not examine populations or interventions of interest, or did not report outcome measures of interest*

Full-text articles assessed for eligibility
(n = 22)

Full-text articles excluded (n = 21)

*Citations did not provide evidence matching questions of interest*

Studies considered
for evidence report
(n = 1)

Studies excluded during data extraction
(n = 0)

Studies matched

to this part
(n = 1)

**PART 3: PHYSICAL EXAMINATION (Question 5-8)**

Records identified through searching of PubMed (n=266), Embase (n=692) and Cochrane Library (n = 76)

Total records screened after duplicates and
non-English publications removed
(n = 883)

Total excluded after title and abstract screening (n = 827)

*Citations did not match study designs of interest, did not examine populations or interventions of interest, or did not report outcome measures of interest*

Full-text articles assessed for eligibility
(n = 56)

Full-text articles excluded (n = 33)

*Citations did not provide evidence matching questions of interest*

Studies considered
for evidence report
(n = 23)

Studies excluded during data extraction
(n = 0)

Studies matched

to this part
(n = 23)

**PART 4: ULTRASOUND (Question 9-17)**

Records identified through searching of PubMed (n=481), Embase (n=545) and Cochrane Library (n = 13)

Total records screened after duplicates and
non-English publications removed
(n = 894)

Total excluded after title and abstract screening (n = 809)

*Citations did not match study designs of interest, did not examine populations or interventions of interest, or did not report outcome measures of interest*

Full-text articles assessed for eligibility
(n = 85)

Full-text articles excluded (n = 54)

*Citations did not provide evidence matching questions of interest*

Studies considered
for evidence report
(n = 31)

Studies excluded during data extraction
(n = 0)

Studies matched

to this part
(n = 31)

**PART 5: MRI (Question 18-22)**

Records identified through searching of PubMed (n=283), Embase (n=828) and Cochrane Library (n = 34)

Total records screened after duplicates and
non-English publications removed
(n = 997)

Total excluded after title and abstract screening (n = 910)

*Citations did not match study designs of interest, did not examine populations or interventions of interest, or did not report outcome measures of interest*

Full-text articles assessed for eligibility
(n = 87)

Full-text articles excluded (n = 54)

*Citations did not provide evidence matching a question of interest*

Studies considered
for evidence report
(n = 33)

Studies excluded during data extraction
(n = 0)

Studies matched

to this part
(n = 33)

**PART 6: X-RAY (Question 23)**

Records identified through searching of PubMed (n=277), Embase (n=586) and Cochrane Library (n = 15)

Total records screened after duplicates and
non-English publications removed
(n = 773)

Total excluded after title and abstract screening (n = 745)

*Citations did not match study designs of interest, did not examine populations or interventions of interest, or did not report outcome measures of interest*

Full-text articles assessed for eligibility
(n = 28)

Full-text articles excluded (n = 23)

*Citations did not provide evidence matching questions of interest*

Studies considered
for evidence report
(n = 5)

Studies excluded during data extraction
(n = 0)

Studies matched

to this part
(n = 5)

**PART 7: PET/CT (Question 24)**

Records identified through searching of PubMed (n=15),

Embase (n=33) and Cochrane Library (n = 0)

Total records screened after duplicates and
non-English publications removed
(n = 40)

Total excluded after title and abstract screening (n = 33)

*Citations did not match study designs of interest, did not examine populations or interventions of interest, or did not report outcome measures of interest*

Full-text articles assessed for eligibility
(n = 7)

Full-text articles excluded (n = 4)

*Citations did not provide evidence matching questions of interest*

Studies considered
for evidence report
(n = 3)

Studies excluded during data extraction
(n = 0)

Studies matched

to this part
(n = 3)
