# Supplementary material for: A clinical practice guideline for the screening and assessment of enthesitis in patients with spondyloarthritis
Source: Front Immunol. 2022 Sep 12;13:978504. doi: 10.3389/fimmu.2022.978504 (PMC9510351; doi:10.3389/fimmu.2022.978504)
Supplement: Supplementary file 5 [file DataSheet_5.docx]

**NOS Scores of Selected Articles**

**PART 1: OVERVIEW (Question1-3)**

**Question 1:**

Table 1:

| Author | Publish year | Design | Country | Patient diagnosis | Patient number | Control type | Control number | Age (year) Patient/control | NOS score | Database |
| --- | --- | --- | --- | --- | --- | --- | --- | --- | --- | --- |
| Poulain | 2018 | cohort | France | recent-onset IBP | 708 | - | - | 35.5 (28.7-44) and 32.91 (26.4-39.1)/- | 4 | PubMed, Embase |
| Eder | 2014 | cross-sectional | Canada | PsA/PsO | 50/66 | HC | 60 | 53.2±12.5 and 51.2±14/42.4±12.7 | 5 | PubMed, Embase |
| D'Agostino | 2011 | Cohort | France | SpA | 51 | Non-SpA | 48 | 38.3(18.5-68.5) | 5 | PubMed, Embase |
| de Miguel | 2011 | cross-sectional | Spain | early spondyloarthritis | 113 | HC | 57 | 32.69±7.52/36.17±9.8 | 6 | PubMed, Embase |
| de Miguel | 2009 | cross-sectional | Spain | SpA | 25 | HC | 29 | 43.3±15.6/46.1±13.4 | 6 | PubMed |

**Question 2:**

Table 2:

| Author | Publish year | Design | Country | Patient diagnosis | Patient number | Control type | Control number | Age (year) Patient/control | NOS score | Database |
| --- | --- | --- | --- | --- | --- | --- | --- | --- | --- | --- |
| Mease | 2020 | Cross-sectional | United States | SpA | 477 | - | - | 47.3±13.1 and 47.3±14.1/- | 4 | PubMed, Embase |
| Sunar | 2020 | Cross-sectional | Turkey | PsA | 1130 | - | - | 46.9±12.2/- | 3 | PubMed, Embase |
| Strand | 2019 | Cross-sectional | United States | axSpA | 5660 | PsA | 3570 | mean 42.2/47.5 | 3 | Embase |
| Behrens | 2019 | Clinical trial | Germany | SpA | 76 | PsA | 128 | 45.1±10.9/49.4±11.1 | 5 | Embase, Cochrane |
| Kwan | 2019 | Cohort | Singapore | axSpA | 138 | - | - | 39.3±17.1/- | 4 | PubMed, Embase |
| de Winter | 2019 | Cohort | Netherlands | axSpA | 230 | pSpA | 84 | median 41 (IQR 33-52)/48 (37-56) | 4 | PubMed, Embase |
| Laatiris | 2012 | Cross-sectional | Morocco | AS | 76 | - | - | 38±12.9/- | 3 | PubMed, Embase |
| Turan | 2007 | Cross-sectional | Turkey | AS | 46 | - | - | 39.2±11.46/- | 3 | PubMed, Embase |

**Question 3:**

Table 3:

| Author | Publish year | Design | Country | Patient diagnosis | Patient number | Control type | Control number | Age (year) Patient/control | NOS score | Database |
| --- | --- | --- | --- | --- | --- | --- | --- | --- | --- | --- |
| Solmaz | 2020 | Cross-sectional | Canada | axSpA | 120 | - | - | 45.3 ±13.8/- | 5 | PubMed, Embase |
| Ruyssen-Witrand | 2017 | Cohort | France | SpA | 402 | - | - | median 33.5 (IQR 26.7–39.4)/- | 3 | PubMed, Embase |
| Polachek | 2017 | Cross-sectional | Canada | PsA | 223 | - | - | 56±12.9/- | 3 | PubMed, Embase |
| Costantino | 2017 | Cohort | France | SpA | 953 | - | - | 24.6±9.6/- | 5 | PubMed, Embase |
| Aydin | 2016 | Case-control | Turkey | AS | 225 | HC | 95 | median 34 (range 18-76)/35 (20-80) | 6 | PubMed, Embase |
| Poddubnyy | 2012 | Cohort | Germany | axSpA | 210 | - | - | 37.1±10.6/- | 4 | PubMed, Embase |

**PART 2: HISTORY TAKING (Question 4)**

**Question 4:**

Table 4:

| Author | Publish year | Design | Country | Patient diagnosis | Patient number | Control type | Control number | Age (year) Patient/control | NOS score | Database |
| --- | --- | --- | --- | --- | --- | --- | --- | --- | --- | --- |
| Klauser | 2008 | Cohort | Austria | multiple enthesitis | 21 | RA/non-rheumatic diseases | 12 | median 51（range 30-75)/65（56-77） and 55(45-65) | 4 | PubMed, Embase |

**PART 3: PHYSICAL EXAMINATION (Question 5-8)**

**Question 5:**

Table 5:

| Author | Publish year | Design | Country | Patient diagnosis | Patient number | Control type | Control number | Age (year) Patient/control | NOS score | Database |
| --- | --- | --- | --- | --- | --- | --- | --- | --- | --- | --- |
| Fiorenza | 2020 | Cross-sectional | Italy | PsA | 39 | - | - | median（IQR）56（9）/- | 5 | PubMed, Embase |
| Macchioni | 2019 | Cross-sectional | Italy | PsA | 140 | - | - | median(IQR range) 48 (40–58)/- | 4 | PubMed, Embase |
| Zhang | 2017 | Cohort | China | AS | 20 | - | - | 25.5±9.6/- | 5 | PubMed, Embase |
| Michelsen | 2017 | cross-sectional | Norway | PsA | 141 | - | - | 52.4±10.2 | 4 | Embase |
| Althoff | 2016 | Cohort | Germany | axSpA | 41 | - | - | 32.8±8.1/- | 5 | PubMed, Embase |
| Poggenborg | 2013 | Cross-sectional | Denmark | PsA/axSpA | 18/18 | HC | 12 | median 49 (IQR 37–58) and 42 (32–52) /32 (27–47) | 4 | PubMed, Embase |
| Bandinelli | 2013 | Cross-sectional | Italy | ePsA | 92 | HC | 40 | 51±15/49.6±11.1 | 5 | PubMed, Embase |
| Spadaro | 2011 | Cross-sectional | Italy | AS | 36 | - | - | mean 51.3 (range 23-75)/- | 4 | PubMed, Embase |
| Ruta | 2011 | Cross-sectional | Italy | SpA | 60 | HC | 30 | 37±14/38±13 | 6 | PubMed |
| Laatiris | 2010 | Cross-sectional | Morocco | AS | 76 | - | - | 38±12.9/- | 3 | PubMed, Embase |
| Klauser | 2008 | Cohort | Austria | multiple enthesitis | 21 | RA/non-rheumatic diseases | 12 | median 51（range 30-75)/65（56-77） and 55(45-65) | 4 | PubMed, Embase |
| Genc | 2005 | Cross-sectional | Turkey | AS | 18 | HC | 20 | 41.38±8.49/42.60±10.51 | 5 | PubMed, Embase |
| Balint | 2002 | Cross-sectional | United Kingdom | SpA | 35 | - | - | mean 48 (median 49.3)/- | 5 | PubMed, Embase |

**Question 6:**

Table 6:

| Author | Publish year | Design | Country | Patient diagnosis | Patient number | Control type | Control number | Age (year) Patient/control | NOS score | Database |
| --- | --- | --- | --- | --- | --- | --- | --- | --- | --- | --- |
| Zhang | 2017 | Cross-sectional | China | AS | 20 | - | - | 25.5±9.6/- | 5 | PubMed, Embase |
| Wiell | 2012 | Case-control | Denmark | SpA/non-SpA | 12/15 | HC | 10 | median 38.5 (range 27-59)/47 (31-60) | 4 | PubMed |
| Spadaro | 2011 | Cross-sectional | Italy | AS | 36 | - | - | mean 51.3 (range 23-75)/- | 4 | PubMed, Embase |
| Klauser | 2008 | Cohort | Austria | multiple enthesitis | 21 | RA/non-rheumatic diseases | 12 | median 51（range 30-75)/65（56-77） and 55(45-65) | 4 | PubMed, Embase |
| Balint | 2002 | Cross-sectional | United Kingdom | SpA | 35 | - | - | mean 48 (median 49.3)/- | 5 | PubMed, Embase |

**Question 7:**

Table 7:

| Author | Publish year | Design | Country | Patient diagnosis | Patient number | Control type | Control number | Age (year) Patient/control | NOS score | Database |
| --- | --- | --- | --- | --- | --- | --- | --- | --- | --- | --- |
| Heuft-Dorenbosch | 2003 | Cross-sectional | Netherlands | AS | 162 | - | - | 44.9±12.3/- | 4 | PubMed, Embase |
| Healy | 2008 | Cohort | New Zealand | PsA | 28 | - | - | 46.5±10.5/- | 3 | PubMed, Embase |
| Hamdi | 2011 | Cross-sectional | Tunisia | AS | 60 | - | - | 36±11/- | 3 | PubMed, Embase |

**Question 8:**

Table 8:

| Author | Publish year | Design | Country | Patient diagnosis | Patient number | Control type | Control number | Age (year) Patient/control | NOS score | Database |
| --- | --- | --- | --- | --- | --- | --- | --- | --- | --- | --- |
| Seven | 2020 | Clinical trial | United Kingdom | SpA | 21 | - | - | 39.4±9.4/- | 4 | Embase, Cochrane |
| Lee | 2020 | Clinical trial | Korea | AS | 201 | - | - | 39.8±12.3/- | 5 | PubMed, Embase |
| Gladman | 2018 | Clinical trial | Canada | PsA | 327 | - | - | mean 50.8/- | 5 | PubMed, Embase, Cochrane |
| Hartung | 2018 | Cohort | Germany | SpA | 145 | - | - | 47.5±11/- | 6 | PubMed, Embase |
| Van der Heijde | 2013 | Clinical trial | Netherlands | AS | 140 | - | - | - | 4 | PubMed, Embase, Cochrane |
| Rudwaleit | 2010 | Clinical trial | Germany | AS | 667 | - | - | 42.7±11.1/- | 4 | PubMed, Embase |
| Healy | 2008 | Cohort | United Kingdom | PsA | 28 | - | - | 46.5±10.5/- | 3 | PubMed, Embase |

**PART 4: ULTRASOUND (Question 9-17)**

**Question 9:**

Table 9:

| Author | Publish year | Design | Country | Patient diagnosis | Patient number | Control type | Control number | Age (year) Patient/control | NOS score | Database |
| --- | --- | --- | --- | --- | --- | --- | --- | --- | --- | --- |
| Zhang | 2017 | Cohort | China | AS | 20 | - | - | 25.5±9.6/- | 5 | PubMed, Embase |
| Spadaro | 2011 | Cross-sectional | Italy | AS | 36 | - | - | mean 51.3 (range 23-75)/- | 4 | PubMed, Embase |
| Alcalde | 2007 | Cohort | Spain | AS | 44 | HC | 10 | mean 43 (range 24-72)/- | 4 | PubMed, Embase |
| Balint | 2002 | Cross-sectional | United Kingdom | SpA | 35 | - | - | mean 48 (median 49.3)/- | 5 | PubMed, Embase |

**Question 10:**

Table 10:

| Author | Publish year | Design | Country | Patient diagnosis | Patient number | Control type | Control number | Age (year) Patient/control | NOS score | Database |
| --- | --- | --- | --- | --- | --- | --- | --- | --- | --- | --- |
| Ozsoy-unubol | 2018 | Cross-sectional | Turkey | nr-axSpA | 30 | mechanical back pain | 30 | 31.73±5.68/30.90±7.41 | 6 | PubMed, Embase |
| Poulain | 2018 | Cohort | France | recent-onset IBP | 708 | - | - | 35.5 (28.7–44) and 32.91 (26.4–39.1) | 4 | PubMed, Embase |
| Aydin | 2014 | Cross-sectional | Turkey | SpA(ESSG) | 55 | HC | 46 | 40.2±10.7/36.4±10 | 5 | PubMed, Embase |
| Feydy | 2011 | Cross-sectional | France | SpA(Amor) | 51 | non-IBP | 24 | median 45 ( range 35-64) and 50 (37-59) and43 (39-49)/50 (42-55) | 5 | PubMed, Embase |
| Hu | 2011 | Cross-sectional | China | AS | 161 | - | - | 26±9 and 30±11/- | 5 | PubMed, Embase |
| D'Agostino | 2011 | Cohort | France | SpA | 51 | Non-SpA | 48 | 38.3(18.5-68.5) | 5 | PubMed, Embase |
| de Miguel | 2011 | Cross-sectional | Spain | early spondyloarthritis | 113 | HC | 57 | 32.69±7.52/36.17±9.8 | 6 | PubMed, Embase |
| de Miguel | 2009 | Cross-sectional | Spain | SpA(ESSG) | 25 | HC | 29 | 43.3±15.6/46.1±13.4 | 5 | PubMed |

**Question 11:**

Table 11:

| Author | Publish year | Design | Country | Patient diagnosis | Patient number | Control type | Control number | Age (year) Patient/control | NOS score | Database |
| --- | --- | --- | --- | --- | --- | --- | --- | --- | --- | --- |
| Ishida | 2019 | Cross-sectional | Brazil | SpA | 50 | HC | 30 | 43.44±9.91/38.70±8.52 | 3 | PubMed, Embase |
| Ozsoy-unubol | 2018 | Cross-sectional | Turkey | nr-axSpA | 30 | mechanical back pain | 30 | 31.73±5.68/30.90±7.41 | 6 | PubMed, Embase |
| Ruta | 2014 | Cohort | Argentina | SpA | 34 | - | - | median 31 (interquartile rang,25-78:38-62) | 4 | PubMed, Embase |
| Falcao | 2014 | Cohort | Spain | SpA | 146 | - | - | 32.4±7.4/- | 4 | PubMed, Embase |
| Aydin | 2010 | Cohort | Turkey | AS | 43 | - | - | 35.4±12.5/- | 4 | PubMed, Embase |
| Naredo | 2010 | Cohort | Spain | SpA(ESSG) | 327 | - | - | 44.5±11.3/- | 5 | PubMed, Embase |
| de Miguel | 2009 | Cross-sectional | Spain | SpA(ESSG) | 25 | HC | 29 | 43.3±15.6/46.1±13.4 | 5 | PubMed |
| Alcalde | 2007 | Cohort | Spain | AS | 44 | HC | 10 | mean 43 (range 24-72)/- | 4 | PubMed, Embase |

**Question 12:**

Table 12.1:

| Author | Publish year | Design | Country | Patient diagnosis | Patient number | Control type | Control number | Age (year) Patient/control | NOS score | Database |
| --- | --- | --- | --- | --- | --- | --- | --- | --- | --- | --- |
| Seven | 2020 | Clinical trial | Denmark | axSpA | 21 | - | - | 39.4±9.4/- | 4 | Embase, Cochrane |
| Ishida | 2019 | Cross-sectional | Brazil | AS | 50 | HC | 30 | 43.44±9.91/38.70±8.52 | 3 | PubMed, Embase |
| Ruyssen-Witrand | 2017 | Cohort | France | SpA | 402 | - | - | median 33.5 (IQR 26.7–39.4)/- | 3 | PubMed, Embase |
| Zhang | 2017 | Cross-sectional | China | AS | 20 | - | - | 25.5±9.6/- | 5 | PubMed, Embase |
| Harman | 2017 | Cross-sectional | Turkey | AS/RA | 40/27 | HC | 30 | 43.37±9.55 and 45.63±11.76/42.13±4.93 | 6 | PubMed, Embase |
| Baraliakos | 2017 | Cross-sectional | Germany | pSpA | 30 | non-SpA | 30 | 37.5±5.9/36.9±7.7 | 4 | PubMed, Embase |
| Wink | 2017 | Cohort | Netherland | AS | 111 | - | - | 42.9 ± 10.9/- | 6 | PubMed, Embase |
| Sudol-Szopinska | 2014 | Cross-sectional | Poland | enthesitis | 68 | - | - | median 67.5 (range 41-93)/- | 3 | Embase |
| Wiell | 2012 | Cross-sectional | Denmark | SpA | 12 | non-SpA | 15 | median 38.5 (range 27-59)/47 (31-60) | 5 | PubMed |
| Hamdi | 2011 | Cross-sectional | Tunisia | AS | 60 | - | - | 36±11/- | 3 | PubMed, Embase |
| Spadaro | 2011 | Cross-sectional | Italy | AS | 36 | - | - | mean 51.3 (range 23-75)/- | 4 | PubMed, Embase |
| Feydy | 2011 | Cross-sectional | France | SpA(Amor) | 51 | non-IBP | 24 | median 45 ( range 35-64) and 50 (37-59) and43 (39-49)/50 (42-55) | 5 | PubMed, Embase |
| Naredo | 2010 | Cohort | Spain | SpA(ESSG) | 327 | - | - | 44.5±11.3/- | 5 | PubMed, Embase |
| Kiris | 2006 | Cross-sectional | Turkey | AS | 30 | - | - | 34.1±6.5/- | 3 | PubMed, Embase |
| Balint | 2002 | Cross-sectional | United Kingdom | SpA | 35 | - | - | mean 48 (median 49.3)/- | 5 | PubMed, Embase |

Table 12.2:

| Author | Publish year | Design | Country | Patient diagnosis | Patient number | Control type | Control number | Age (year) Patient/control | NOS score | Database |
| --- | --- | --- | --- | --- | --- | --- | --- | --- | --- | --- |
| Seven | 2020 | Clinical trial | Denmark | axSpA | 21 | - | - | 39.4±9.4/- | 4 | Embase, Cochrane |
| Ishida | 2019 | Cross-sectional | Brazil | AS | 50 | HC | 30 | 43.44±9.91/38.70±8.52 | 3 | PubMed, Embase |
| Zhang | 2017 | Cohort | China | AS | 20 | - | - | 25.5±9.6/- | 5 | PubMed, Embase |
| Baraliakos | 2017 | Cross-sectional | Germany | pSpA | 30 | non-SpA | 30 | 37.5±5.9/36.9±7.7 | 4 | PubMed, Embase |
| Wink | 2017 | Cohort | Netherland | AS | 111 | - | - | 42.9 ± 10.9/- | 6 | PubMed, Embase |
| Spadaro | 2011 | Cross-sectional | Italy | AS | 36 | - | - | mean 51.3 (range 23-75)/- | 4 | PubMed, Embase |
| Hamdi | 2011 | Cross-sectional | Tunisia | AS | 60 | - | - | 36±11/- | 3 | PubMed, Embase |
| Feydy | 2011 | Cross-sectional | France | SpA(Amor) | 51 | non-IBP | 24 | median 45 ( range 35-64) and 50 (37-59) and43 (39-49)/50 (42-55) | 5 | PubMed, Embase |
| Naredo | 2010 | Cohort | Spain | SpA(ESSG) | 327 | - | - | 44.5±11.3/- | 5 | PubMed, Embase |
| Balint | 2002 | Cross-sectional | United Kingdom | SpA | 35 | - | - | mean 48 (median 49.3)/- | 5 | PubMed, Embase |

Table 12.3:

| Author | Publish year | Design | Country | Patient diagnosis | Patient number | Control type | Control number | Age (year) Patient/control | NOS score | Database |
| --- | --- | --- | --- | --- | --- | --- | --- | --- | --- | --- |
| Seven | 2020 | Clinical trial | Denmark | axSpA | 21 | - | - | 39.4±9.4/- | 4 | Embase, Cochrane |
| Ishida | 2019 | Cross-sectional | Brazil | AS | 50 | HC | 30 | 43.44±9.91/38.70±8.52 | 3 | PubMed, Embase |
| Zhang | 2017 | Cohort | China | AS | 20 | - | - | 25.5±9.6/- | 5 | PubMed, Embase |
| Spadaro | 2011 | Cross-sectional | Italy | AS | 36 | - | - | mean 51.3 (range 23-75)/- | 4 | PubMed, Embase |
| Hamdi | 2011 | Cross-sectional | Tunisia | AS | 60 | - | - | 36±11/- | 3 | PubMed, Embase |
| Naredo | 2010 | Cohort | Spain | SpA(ESSG) | 327 | - | - | 44.5±11.3/- | 5 | PubMed, Embase |
| Balint | 2002 | Cross-sectional | United Kingdom | SpA | 35 | - | - | mean 48 (median 49.3)/- | 5 | PubMed, Embase |

Table 12.4:

| Author | Publish year | Design | Country | Patient diagnosis | Patient number | Control type | Control number | Age (year) Patient/control | NOS score | Database |
| --- | --- | --- | --- | --- | --- | --- | --- | --- | --- | --- |
| Seven | 2020 | Clinical trial | Denmark | axSpA | 21 | - | - | 39.4±9.4/- | 4 | Embase, Cochrane |
| Ishida | 2019 | Cross-sectional | Brazil | AS | 50 | HC | 30 | 43.44±9.91/38.70±8.52 | 3 | PubMed, Embase |
| Ruyssen-Witrand | 2017 | Cohort | France | SpA | 402 | - | - | median 33.5 (IQR 26.7–39.4) | 3 | PubMed, Embase |
| Zhang | 2017 | Cohort | China | AS | 20 | - | - | 25.5±9.6/- | 5 | PubMed, Embase |
| Baraliakos | 2017 | Cross-sectional | Germany | pSpA | 30 | non-SpA | 30 | 37.5±5.9/36.9±7.7 | 4 | PubMed, Embase |
| Wink | 2017 | Cohort | Netherland | AS | 1111 | - | - | 42.9 ± 10.9/- | 6 | PubMed, Embase |
| Spadaro | 2011 | Cross-sectional | Italy | AS | 36 | - | - | mean 51.3 (range 23-75)/- | 4 | PubMed, Embase |
| Hamdi | 2011 | Cross-sectional | Tunisia | AS | 60 | - | - | 36±11/- | 3 | PubMed, Embase |
| Naredo | 2010 | Cohort | Spain | SpA(ESSG) | 327 | - | - | 44.5±11.3/- | 5 | PubMed, Embase |
| Balint | 2002 | Cross-sectional | United Kingdom | SpA | 35 | - | - | mean 48 (median 49.3)/- | 5 | PubMed, Embase |

**Question 13:**

Table 13:

| Author | Publish year | Design | Country | Patient diagnosis | Patient number | Control type | Control number | Age (year) Patient/control | NOS score | Database |
| --- | --- | --- | --- | --- | --- | --- | --- | --- | --- | --- |
| Ishida | 2019 | Cross-sectional | Brazil | AS | 50 | HC | 30 | 43.44±9.91/38.70±8.52 | 3 | PubMed, Embase |
| Ozsoy-unubol | 2018 | Cross-sectional | Turkey | nr-axSpA(ASAS) | 30 | mechanical back pain | 30 | 31.73±5.68/30.90±7.41 | 6 | PubMed, Embase |
| Harman | 2017 | Cross-sectional | Turkey | AS/RA | 40/27 | HC | 30 | 43.37±9.55/45.63±11.76  /42.13±4.93 | 6 | PubMed, Embase |
| Wiell | 2012 | Cross-sectional | Denmark | SpA | 12 | non-SpA | 15 | median 38.5 (range 27-59)/47 (31-60) | 5 | PubMed |
| D'Agostino | 2011 | Cohort | France | SpA | 51 | Non-SpA | 48 | 38.3(18.5-68.5) | 5 | PubMed, Embase |
| Feydy | 2011 | Cross-sectional | France | SpA(Amor) | 51 | non-IBP | 24 | median 45 ( range 35-64) and 50 (37-59) and43 (39-49)/50 (42-55) | 5 | PubMed, Embase |
| D'Agostino | 2003 | Cross-sectional | France | SpA | 164 | mechanical back pain and RA | 64 | 38±12/49±13 and 53±14 | 4 | PubMed, Embase |

**Question 14:**

Table 14:

| Author | Publish year | Design | Country | Patient diagnosis | Patient number | Control type | Control number | Age (year) Patient/control | NOS score | Database |
| --- | --- | --- | --- | --- | --- | --- | --- | --- | --- | --- |
| Seven | 2020 | Clinical trial | Denmark | axSpA | 21 | - | - | 39.4±9.4/- | 4 | Embase, Cochrane |
| Ozsoy-unubol | 2018 | Cross-sectional | Turkey | nr-axSpA(ASAS) | 30 | mechanical back pain | 30 | 31.73±5.68/30.90±7.41 | 6 | PubMed, Embase |
| Zhang | 2017 | Cohort | China | AS | 20 | - | - | 25.5±9.6/- | 5 | PubMed, Embase |
| Wink | 2017 | cohort | Netherland | AS | 111 | - | - | 42.9 ± 10.9/- | 6 | PubMed, Embase |
| Spadaro | 2011 | Cross-sectional | Italy | AS | 36 | - | - | mean 51.3 (range 23-75)/- | 4 | PubMed, Embase |
| D'Agostino | 2011 | Cohort | France | SpA | 51 | Non-SpA | 48 | 38.3(18.5-68.5)  /41.6(18.2-85.3) | 5 | PubMed, Embase |
| D'Agostino | 2003 | Cross-sectional | France | SpA | 164 | mechanical back pain and RA | 64 | 38±12/49±13 and 53±14 | 4 | PubMed, Embase |

**Question 15:**

Table 15:

| Author | Publish year | Design | Country | Patient diagnosis | Patient number | Control type | Control number | Age (year) Patient/control | NOS score | Database |
| --- | --- | --- | --- | --- | --- | --- | --- | --- | --- | --- |
| Ishida | 2019 | Cross-sectional | Brazil | AS | 50 | HC | 30 | 43.44±9.91/38.70±8.52 | 3 | PubMed, Embase |
| Ozsoy-unubol | 2018 | Cross-sectional | Turkey | nr-axSpA | 30 | mechanical back pain | 30 | 31.73±5.68/30.90±7.41 | 6 | PubMed, Embase |
| Lanfranchi | 2017 | Cross-sectional | France | axSpA | 30 | athletes and non-athlete healthy | 30/29 | 36.1±6.8/29.3±8.9 and 30.1±8.0 | 4 | PubMed, Embase |
| de Miguel | 2011 | Cross-sectional | Spain | early spondyloarthritis | 113 | HC | 57 | 32.69±7.52/36.17±9.8 | 6 | PubMed, Embase |
| de Miguel | 2009 | Cross-sectional | Spain | SpA(ESSG) | 25 | HC | 29 | 43.3±15.6/46.1±13.4 | 5 | PubMed |

**Question 16:**

Table 16:

| Author | Publish year | Design | Country | Patient diagnosis | Patient number | Control type | Control number | Age (year) Patient/control | NOS score | Database |
| --- | --- | --- | --- | --- | --- | --- | --- | --- | --- | --- |
| Ozsoy-unubol | 2018 | Cross-sectional | Turkey | nr-axSpA(ASAS) | 30 | mechanical back pain | 30 | 31.73±5.68/30.90±7.41 | 6 | PubMed, Embase |
| Ruyssen-Witrand | 2017 | Cohort | France | SpA | 402 | - | - | median 33.5 (IQR 26.7–39.4) | 3 | PubMed, Embase |
| Harman | 2017 | Cross-sectional | Turkey | AS/RA | 40/27 | HC | 30 | 43.37±9.55/45.63±11.76  /42.13±4.93 | 6 | PubMed, Embase |
| Lanfranchi | 2017 | Cross-sectional | France | axSpA | 30 | athletes and non-athlete healthy | 30/29 | 36.1±6.8/29.3±8.9 and 30.1±8.0 | 4 | PubMed, Embase |
| Wink | 2017 | Cohort | Netherland | AS | 111 | - | - | 42.9 ± 10.9/- | 6 | PubMed, Embase |
| Wang | 2015 | Cohort | China | AS | 100 | - | - | 34.4±9.1 | 3 | PubMed, Embase |
| Ruta | 2014 | Cohort | Argentina | SpA(ASAS) | 34 | - | - | median 31 (interquartile rang,25-78:38-62) | 4 | PubMed, Embase |
| Naredo | 2010 | Cohort | Spain | SpA(ESSG) | 327 | - | - | 44.5±11.3/- | 5 | PubMed, Embase |
| Aydin | 2010 | Cohort | Turkey | AS | 43 | - | - | 35.4±12.5/- | 4 | PubMed, Embase |
| Alcalde | 2007 | Cohort | Spain | AS | 44 | HC | 10 | mean 43 (range 24-72)/- | 4 | PubMed, Embase |
| Kiris | 2006 | Cross-sectional | Turkey | AS | 30 | - | - | 34.1±6.5/- | 3 | PubMed, Embase |
| Borman | 2005 | Cross-sectional | Turkey | SpA(ESSG) | 44 | - | - | 39.5±12.5/- | 3 | PubMed, Embase |
| Balint | 2002 | Cross-sectional | United Kingdom | SpA | 35 | - | - | mean 48 (median 49.3)/- | 5 | PubMed, Embase |

**Question 17:**

Table 17:

| Author | Publish year | Design | Country | Patient diagnosis | Patient number | Control type | Control number | Age (year) Patient/control | NOS score | Database |
| --- | --- | --- | --- | --- | --- | --- | --- | --- | --- | --- |
| Seven | 2020 | Clinical trial | Denmark | axSpA | 21 | - | - | 39.4±9.4/- | 4 | Embase, Cochrane |
| Hartung | 2018 | Cohort | Germany | SpA | 145 | - | - | 47.5±11/- | 6 | PubMed, Embase |
| Zhang | 2017 | Cohort | China | AS | 20 | - | - | 25.5±9.6/- | 5 | PubMed, Embase |
| Wink | 2017 | cohort | Netherland | AS | 111 | - | - | 42.9 ± 10.9/- | 6 | PubMed, Embase |
| Wang | 2015 | Cohort | China | AS | 100 | - | - | 34.4±9.1/- | 3 | PubMed, Embase |
| Ruta | 2014 | Cohort | Argentina | SpA(ASAS) | 34 | - | - | median 31 (interquartile rang,25-78:38-62)/- | 4 | PubMed, Embase |
| Naredo | 2010 | Cohort | Spain | SpA(ESSG) | 327 | - | - | 44.5±11.3/- | 5 | PubMed, Embase |
| Aydin | 2009 | Cohort | Turkey | AS | 43 | - | - | 35.4±12.5/- | 4 | PubMed, Embase |

**PART 5: MRI (Question 18-22)**

**Question 18:**

Table 18:

| Author | Publish year | Design | Country | Patient diagnosis | Patient number | Control type | Control number | Age (year) Patient/control | NOS score | Database |
| --- | --- | --- | --- | --- | --- | --- | --- | --- | --- | --- |
| Kleinrensink | 2020 | Cohort | Netherlands | PsO/PsA/AS | 13/13/12 | - | - | median 41.4 (IQR30.0-52.3) and 50.5 (42.4-52.8) and 48.5 (37.9-51.9)/- | 5 | PubMed, Embase |
| Renson | 2020 | Cohort | Belgium | pSpA | 32 | - | - | 37.4±12.7/- | 3 | Embase |
| Aguila Maldonado | 2017 | Cross-sectional | Argentina | SpA | 40 | - | - |  | 6 | PubMed, Embase |
| Lorenzin | 2016 | Cohort | Italy | SpA | 60 | - | - | 29.05±8.38/- | 4 | PubMed, Embase |
| Herregods | 2015 | Cohort | Belgium | enthesitis-related arthritis | 143 | - | - | median 14.3 (range 7-16)/- | 4 | Embase |
| Klang | 2014 | Cohort | Israel | Enthesitis | 67 | - | - | mean 53 | 4 | Embase |
| Aydin | 2013 | Cohort | Turkey | SpA | 21 | - | - | 41.1±17.9/- | 4 | PubMed, Embase |
| Jans | 2013 | Cohort | Belgium | SpA | 176 | non-SpA | 268 | median 30.9 (range 17.0-44.9)/34.7 (17.3–44.9) | 4 | PubMed, Embase |
| Paramarta | 2013 | Case-control | Netherlands | SpA/RA/CA | 13/20/8 | - | - | median 46 ( range 32–51) and 53 (39–57) and 53 (48–61)/- | 4 | PubMed, Embase |
| Wiell | 2012 | Case-control | Denmark | SpA/non-SpA | 12/15 | HC | 10 | median 38.5 (range 27-59)/47 (31-60) | 4 | PubMed |
| Emad | 2009 | Case-control | Netherlands | UA/RA/SpA | 25/15/15 | - | - | 33.0±5.7 and 43.3±9.3 and 39.5±7.3/- | 4 | PubMed, Embase |
| Kamel | 2002 | Cohort | Arabiau | SpA | 32 | - | - | mean 29 | 4 | Embase |

**Question 19:**

Table 19:

| Author | Publish year | Design | Country | Patient diagnosis | Patient number | Control type | Control number | Age (year) Patient/control | NOS score | Database |
| --- | --- | --- | --- | --- | --- | --- | --- | --- | --- | --- |
| Krabbe | 2020 | Cohort | Denmark | axSpA | 53 |  |  | mean 37.5 (median 35, IQR 28–44)/- | 5 | PubMed |
| Poulsen | 2020 | Cross-sectional | Denmark | PsA/RA | 14/10 | HC | 16 | median 48 (range 31-68) and 49 (26-58)/35 (23-54) | 4 | PubMed |
| Krabbe | 2019 | RCT | Denmark | axSpA | 50 | - | - | median 36 (IQR 30-44) | 5 | PubMed, Embase, Cochrane |
| Krabbe | 2019 | Cross-sectional | Denmark | axSpA/RA/PsA | 13/4/4 | - | - | - | 4 | Embase |
| Krabbe | 2018 | RCT | Denmark | axSpA | 49 | - | - | 39.9±10.8 and 35.1±7.8 | 5 | PubMed, Cochrane |
| Althoff | 2016 | Cohort | Germany | axSpA | 41 | - | - | 32.8±8.1/- | 5 |  |
| Poggenborg | 2014 | Cross-sectional | Denmark | PsA/axSpA | 18/18 | HC | 12 | median 49 (IQR 37–58) and 42 (32–52) /32 (27–47) | 4 | PubMed, Embase |
| Karpitschka | 2013 | Cohort | Germany | AS | 10 | - | - | 40±11/- | 5 | PubMed, Embase |
| Althoff | 2012 | Cross-sectional | Germany | axSpA | 75 | - | - | 32.9±8.4/- | 4 | PubMed, Embase |
| Weber | 2012 | Cross-sectional | Canada | axSpA | 122 | HC | 75 | median 33.8（IQR 17.3-71.6）/30.3（17.7-63.8） | 5 | PubMed, Embase |
| Song | 2011 | RCT | Germany | axSpA | 76 | - | - | 33.7±8.5/- | 5 | PubMed, Embase, Cochrane |
| Weckbach | 2011 | Cross-sectional | Germany | PsA | 30 | - | - | mean 47（range 25-78）/- | 5 | PubMed, Embase |

**Question 20:**

Table 20:

| Author | Publish year | Design | Country | Patient diagnosis | Patient number | Control type | Control number | Age (year) Patient/control | NOS score | Database |
| --- | --- | --- | --- | --- | --- | --- | --- | --- | --- | --- |
| Dallaudière | 2018 | Animal experiment | France | immunocompetent Wistar male rats | 12 | - | - | - | 3 | PubMed |
| Chen | 2018 | Cadaveric analysis | USA | fresh cadaveric ankle specimens | 5 | - | - | - | 5 | PubMed |
| Chen | 2018 | Cross-sectional | USA | PsA | 9 | HC | 7 | mean 47（range27-65）/31（28-33） | 4 | PubMed |
| Du | 2010 | Cadaveric analysis | USA | fresh cadaveric ankle specimens | 4 | - | - | - | 3 | PubMed |

**Question 21:**

Table 21:

| Author | Publish year | Design | Country | Patient diagnosis | Patient number | Control type | Control number | Age (year) Patient/control | NOS score | Database |
| --- | --- | --- | --- | --- | --- | --- | --- | --- | --- | --- |
| Sung | 2017 | Cross-sectional | Korea | axSpA | 92 | - | - | mean 34（range 15-63）/- | 5 | PubMed, Embase |
| Klang | 2014 | Cohort | Israel | Enthesitis | 67 | - | - | mean 53/- | 4 | PubMed, Embase |
| de Hooge | 2013 | Cohort | Netherlands. | axSpA | 127 | - | - | 30.8±8.4/- | 4 | PubMed, Embase |
| Weckbach | 2011 | Cross-sectional | Germany | PsA | 30 | - | - | mean 47（range 25-78）/- | 5 | PubMed, Embase |
| Maksymowicz | 2010 | Cross-sectional | Poland | axSpA | 35 | - | - | mean 32.35（range 19-67）/- | 3 | PubMed, Embase |

**Question 22:**

Table 22:

| Author | Publish year | Design | Country | Patient diagnosis | Patient number | Control type | Control number | Age (year) Patient/control | NOS score | Database |
| --- | --- | --- | --- | --- | --- | --- | --- | --- | --- | --- |
| Kleinrensink | 2020 | Cross-sectional | Netherlands | Pso/PsA/AS | 13/13/12 | - | - | median 48.4 (IQR 35.4–52.5)/- | 5 | PubMed, Embase |
| Mathew | 2020 | Atlas | Denmark | SpA/PsA | - | -- | - | - | - | PubMed |
| Mathew | 2019 | Cross-sectional | Denmark | ankles：pSpA/mechanical enthesitis | 4/4 | HC | 2 | - | 3 | PubMed, Embase |

**PART 6: X-RAY (Question 23)**

**Question 23**

Table 23

| Author | Publish year | Design | Country | Patient diagnosis | Patient number | Control type | Control number | Age (year) Patient/control | NOS score | Database |
| --- | --- | --- | --- | --- | --- | --- | --- | --- | --- | --- |
| Kim | 2018 | Cohort | Korea | AS | 40 | - | - | mean 28.68/- | 4 | PubMed |
| Helliwell | 2007 | Case-control | UK | PsA | 588 | RA/AS/uSpA/other | 384/72/38/42 | median50 (range19-87)/ (24-88) and 46 (19–81) and 41 (20-82) and 57 (21-79) | 4 | PubMed |
| Taylor | 2003 | Cohort | New Zealand | PsA | 62 | - | - | - | 4 | PubMed |
| Secundini | 1997 | Cross-sectional | Argentina | AS/PsA/Reiter's syndrome | 19/15/6 | - | - | mean 40.4 | 4 | PubMed, Embase |
| Resnick | 1977 | Case-control | US | RA/AS/PsA/Reiter syndrome | 64/40/26/15 | HC | 75 | mean 57 and 55 and 40 and 33 /46 | 3 | PubMed |

**PART 7: PET/CT (Question 24)**

**Question 24**

Table 24

| Author | Publish year | Design | Country | Patient diagnosis | Patient number | Control type | Control number | Age (year) Patient/control | NOS score | Database |
| --- | --- | --- | --- | --- | --- | --- | --- | --- | --- | --- |
| Kleinrensink | 2020 | Cross-sectional | Netherlands | PsO/PsA/AS | 13/13/12 | - | - | median 48.4 (IQR 35.4–52.5)/- | 5 | PubMed, Embase |
| Bruijnen | 2012 | Cross-sectional | Netherlands | AS | 12 | - | - | median 31.0 (range 25-50) and 41.0 (24-56) | 5 | PubMed, Embase |
| Vijayant | 2012 | Cross-sectional | India | RA/axSpA | 17/11 | - | - | mean 40（range 27-60）and 29.8（17-53）/- | 3 | PubMed, Embase |
