# Supplementary material for: A clinical practice guideline for the screening and assessment of enthesitis in patients with spondyloarthritis
Source: Front Immunol. 2022 Sep 12;13:978504. doi: 10.3389/fimmu.2022.978504 (PMC9510351; doi:10.3389/fimmu.2022.978504)
Supplement: Supplementary file 6 [file DataSheet_6.docx]

**SUPPLEMENTARY APPENDIX: Evidence Report**

**PART 1：OVERVIEW (Question 1-3)**

**Question 1: Diagnostic value of enthesitis screening in patients with Spondyloarthritis**

We found 2 Cohort and 3 Cross-sectional studies addressing this question, which included 944 participants.

The evidence shows:

-In patients suspected of spondyloarthritis, presence of enthesitis by ultrasound, MRI or other approaches might enhance the confidence of diagnosis.

-Detection of peripheral enthesitis may assist in early diagnosis of spondyloarthritis with relatively high sensitivity and specificity.

The quality of evidence is MODERATE.

**Question 2: Enthesitis screening and improvement of life quality in patients with Spondyloarthritis**

We found 5 Cross-sectional, 2 Cohort studies and 1 Clinical trial addressing this question, which included 11615 participants.

The evidence shows:

-Enthesitis composes a significant portion of disease burden in SpA patients.

-SpA patients suffering from enthesitis generally have worse quality of life and impaired mobility.

The quality of evidence is MODERATE.

**Question 3: Prognostic value of enthesitis screening in patients with Spondyloarthritis**

We found 3 Cohort, 2 Cross-sectional and 1 Case-control studies addressing this question, which included 2228 participants.

The evidence shows:

-Regarding the association between enthesitis and radiographic progression of the axial skeleton, results were inconsistent across different studies.

-A few studies have linked the presence of enthesitis to the structural damage in the axial skeleton, either in the spine or in the sacroiliac joint.

-The majority of the US studies identified a correlation between enthesitis and radiographic progression of the spine, especially between enthesophytes and syndesmophytes.

The quality of evidence is LOW.

**Table1: Evidence profile**

| Certainty assessment | | | | | | | Summary of findings | |
| --- | --- | --- | --- | --- | --- | --- | --- | --- |
| No of participants  (studies)  Follow-up | Risk of bias | Inconsistency | Indirectness | Imprecision | Publication bias | Overall certainty of evidence | Pooled Result (95%CI) | Brief Summary |
| Question 1: | | | | | | | | |
| 944 (2 Cohort, 3 Cross-sectional studies) | Serious | Not serious | Not serious | Not serious | Not serious | ⨁⨁⨁◯  MODERATE | \ | In patients suspected of spondyloarthritis, presence of enthesitis by ultrasound, MRI or other approaches might enhance the confidence of diagnosis and facilitate the timely diagnosis with relatively high sensitivity and specificity. |
| Question 2: | | | | | | | | |
| 11615 (5 Cross-sectional, 2 Cohort, 1 Clinical trial) | Serious | Not serious | Not serious | Not serious | Not serious | ⨁⨁⨁◯  MODERATE | \ | Enthesitis composes a significant portion of disease burden in SpA patients. Patients suffering from enthesitis generally have worse quality of life and lower mobility. |
| Question 3: | | | | | | | | |
| 2228 (3 Cohort, 2 Cross-sectional, 1 Case-control) | Serious | Serious | Not serious | Serious | Not serious | ⨁⨁◯◯  LOW | \ | Despite conflicting results, a number of studies identified enthesitis as a risk factor of radiographic progression in axSpA patients, either in the spine or in the sacroiliac joint. |

**Table 2: Studies addressing Question 1**

| Study | Year | Design | Population | Result |
| --- | --- | --- | --- | --- |
| Poulain[1] | 2018 | Cohort | 402 IBP (strong suspicion of axSpA) | PDUS enthesitis was detected in 58 (14.4%) patients of whom 40 (14.2%) belonged to the ASAS+ patients and 18 (17%) to the ASAS- patients. The sensitivity of PDUS enthesitis was 13.9% and the specificity was 83.5%, with a positive predictive value of 69% and 26.8% of negative predictive value for meeting ASAS criteria for axSpA. |
| Eder[2] | 2014 | Cross-sectional | 50 PsA, 66 PsC, 60 HC | Using a cut-off of 20 for the MASEI, considering clinical diagnosis as reference standard, PsA could be diagnosed with a sensitivity of 0.3 and a specificity of 0.89, with a positive LR of 2.63 to differentiate PsA from psoriasis without joint involvement. |
| D’Agostino[3] | 2011 | Cohort | 51 SpA, 48 non-SpA, 19 unclassifed | PDUS detection of at least one vascularised enthesis provided good predictive value for diagnosing SpA (sensitivity 76.5%; specifi city 81.3%; positive likelihood ratio 4.1; OR 14.1; p<0.0001) |
| de Miguel[4] | 2011 | Cross-sectional | 113 SpA, 81 control | The enthesis ultrasound score seems to have diagnostic accuracy and may be useful for improving the diagnostic accuracy of early spondyloarthritis. |
| de Miguel[5] | 2009 | Cross-sectional | 25 SpA, 29 HC | The findings suggest that the ultrasound enthesis score could be a valid tool in the diagnosis of SpA. |

**Table 3: Studies addressing Question 2**

| Study | Year | Design | Population | Result |
| --- | --- | --- | --- | --- |
| Mease[6] | 2020 | Cross-sectional | 477 SpA | Higher proportions of patients with enthesitis had prior biologic and conventional synthetic disease-modifying antirheumatic drug (csDMARD) use and were currently receiving a combination of biologics and csDMARDs than those without enthesitis. Patients with enthesitis had worse disease activity, spinal mobility, and quality of life; greater work impairment; and had a history of depression and fibromyalgia than those without enthesitis. |
| Sunar[7] | 2020 | Cross-sectional | 1130 PsA | Our study found higher frequency of dactylitis and chronic back pain, and worse quality of life determined with SF-36 and PsAQoL scores in patients with enthesitis |
| Strand[8] | 2019 | Cross-sectional | 5660 axSpA, 3570 PsA | After adjusting for confounding factors in propensity score analysis, burden in terms of pain, HRQoL, activity impairment, and HCRU was mostly significantly higher in patients with enthesitis vs those without in axSpA. |
| Behrens[9] | 2019 | Clinical trial | 76 SpA, 128 PsA | In SpA patients suffering from enthesitis, axSpA patients present with a higher burden of disease compared to PsA patients. PsA patients report higher disease burden when compared to patients without enthesitis, suggesting that enthesitis may be an important factor contributing to the burden of disease in PsA. |
| Kwan[10] | 2019 | Cohort | 138 axSpA | History of extra-spinal symptoms (including peripheral arthritis, heel enthesitis, or dactylitis) (HPED) was associated with QoL in patients with axSpA. After 1-year, patients with HPED have poorer QoL especially for physical health domains than patients without HPED. |
| de Winter[11] | 2019 | Cohort | 314 SpA | Half of the patients classified as axial SpA according to the ASAS criteria also have peripheral disease manifestations such as arthritis, enthesitis and/ or dactylitis. These peripheral disease manifestations contribute significantly to overall disease activity. |
| Laatiris[12] | 2012 | Cross-sectional | 76 AS | Severity of enthesitis was significantly correlated with disease activity, functional disability and degradation of quality of life. |
| Turan[13] | 2007 | Cross-sectional | 46 AS | It has been found out that in patients with AS, the QOL subgroups are mostly related with enthesis involvement. |

**Table 4: Studies addressing Question 3**

| Study | Year | Design | Population | Result |
| --- | --- | --- | --- | --- |
| Solmaz[14] | 2020 | Cross-sectional | 120 axSpA | For spinal damage, entheseal damage was an independent and the strongest predictor. |
| Ruyssen-Witrand[15] | 2017 | Cohort | 402 IBP (suggestive of SpA) | There was a correlation between US structural and PDUS scores and the mSASSS. The proportion of patients with syndesmophytes was higher in the case of US enthesophytes. |
| Polachek[16] | 2017 | Cross-sectional | 223 PsA | Regression analyses yielded an association between higher MASEI scores and peripheral joint damage including mSS, joint ankylosis, arthritis mutilans, and periostitis. Similarly, an association was found between higher MASEI scores and axial damage as measured by mSASSS and sacroiliitis. |
| Costantino[17] | 2017 | Cohort | 953 SpA | Factors independently associated with radiographic sacroiliitis at inclusion were male sex, younger age at disease onset, longer disease duration, inflammatory back pain, uveitis and lack of enthesitis. |
| Aydin[18] | 2016 | Case-control | 225 AS, 95 HC | Male AS patients that have more severe US-determined Achilles enthesophyte also associated spinal syndesmophytes suggesting a bone-forming gender-specific phenotype that could be a useful marker predicting of new bone formation. |
| Poddubnyy[19] | 2012 | Cohort | 210 axSpA | No clear association with spinal radiographic progression was observed for HLA-B27 status, sex, age, disease duration, Bath Ankylosing Spondylitis Disease Activity Index, Bath Ankylosing Spondylitis Functional Index, presence of peripheral arthritis, enthesitis, psoriasis, treatment with nonsteroidal antiinflammatory drugs, or treatment with disease-modifying antirheumatic drugs at baseline. |

**PART2：HISTORY TAKING (Question 4)**

**Question 4: The value of history taking in the assessment of enthesitis in patients with spondyloarthritis**

We found 1 Cohort study addressing this question, which included 33 participants.

The evidence shows:

-Research on the value of history taking in the assessment of enthesitis is scarce.

-The only study on this topic revealed that history of chronic enthesitic pain were sensitive and specific for the diagnosis of enthesis inflammation.

The quality of evidence is VERY LOW.

**Table 5: Evidence profile**

| Certainty assessment | | | | | | | Summary of findings | |
| --- | --- | --- | --- | --- | --- | --- | --- | --- |
| No of participants  (studies)  Follow-up | Risk of bias | Inconsistency | Indirectness | Imprecision | Publication bias | Overall certainty of evidence | Pooled Result (95%CI) | Brief Summary |
| Question 4: | | | | | | | | |
| 33 (1 Cohort) | Serious | Not serious | Serious | Serious | Serious | ⨁◯◯◯  VERY LOW | \ | There is very limited research investigating the value of history taking in the assessment of enthesitis. However, symptom evaluation could serve as an initial step, requiring further validation. |

**Table 6: Studies addressing Question 4**

| Study | Year | Design | Population | Result |
| --- | --- | --- | --- | --- |
| Klauser[20] | 2008 | Cohort | 21 Enthesitis  12 Controls | Sensitivity and specificity of selected MASES points were 66.7 – 86.4 % and 85.0 – 91.7 % for history and 71.4 – 87.0 %. At specific enthesal sites, history of chronic enthesitic pain and clinical signs of acute inflammation are sensitive and specific for the diagnosis of chronic and/or acute inflammation. |

**PART 3：PHYSICAL EXAMINATION (Question 5-8)**

**Question 5: The frequency of enthesitis identified by physical examination in patients with Spondyloarthritis**

We found 10 Cross-sectional and 3 Cohort studies addressing this question, which included 737 participants.

The evidence shows:

-Upon physical examination, enthesitis could be found in approximately 19% of SpA patients.

The quality of evidence is MODERATE.

**Question 6: The sensitivity and specificity of physical examination to identify enthesitis in patients with Spondyloarthritis**

We found 2 Cohort and 3 Cross-sectional studies addressing this question, which included 111 participants.

The evidence shows:

-By using US as the reference standard, the sensitivity of physical examination is approximately 20%.

-By using contrast-enhanced MRI as the reference standard, its sensitivity was 58%.

-The specificity of physical examination is consistent across different studies, exceeding 80%.

The quality of evidence is LOW.

**Question 7: The comparison of different physical examination score used to assess enthesitis in patients with Spondyloarthritis**

We found 1 Cohort and 2 Cross-sectional studies addressing this question, which included 305 participants.

The evidence shows:

-Common scoring systems of physical examination of enthesitis include MEI, MASES, SPARCC enthesitis index, LEI, and Gladman index.

-MASES could be considered as a simplified version of MEI, only examining 13 entheses with a total score of 13.

-Compared with MEI, MASES is much less time-consuming and more practical in daily practices.

The quality of evidence is VERY LOW.

**Question 8: The change of physical examination enthesitis score after treatment in patients with Spondyloarthritis**

We found 6 Clinical Trial and 2 Cohort studies addressing this question, which included 2201 participants.

The evidence shows:

-MASES was widely applied as the endpoint in multiple clinical trials to assess the efficacy of treatment on enthesitis.

-Results showed that following treatment, enthesitis was significantly improved as indicated by MASES.

-MASES is a viable tool to monitor the therapeutic responses of enthesitis.

The quality of evidence is LOW.

**Table 7: Evidence profile**

| Certainty assessment | | | | | | | Summary of findings | |
| --- | --- | --- | --- | --- | --- | --- | --- | --- |
| No of participants  (studies)  Follow-up | Risk of bias | Inconsistency | Indirectness | Imprecision | Publication bias | Overall certainty of evidence | Pooled Result (95%CI) | Brief Summary |
| Question 5: | | | | | | | | |
| 737 (10 Cross-sectional, 3  Cohort) | Not serious | Not serious | Not serious | Serious | Not serious | ⨁⨁⨁◯  MODERATE | 0.19  (0.15, 0.25) | Upon physical examination, enthesitis could be found in approximately 19% of SpA patients. |
| Question 6: | | | | | | | | |
| 111 (2 Cohort, 3 Cross-sectional) | Serious | Serious | Not serious | Serious | Not serious | ⨁⨁◯◯  LOW | \ | Compared with other approaches such as US or MRI, sensitivity of physical examination in the detection of enthesitis is very limited, approximately 20%. However, its specificity exceeded 80%. |
| Question 7: | | | | | | | | |
| 305 (1 Cohort, 2 Cross-sectional) | Serious | Serious | Serious | Serious | Not serious | ⨁◯◯◯  VERY LOW | \ | MEI, MASES and SPARCC enthesitis index are some of common scoring methods in the physical examination of enthesitis. Compared with MEI, MASES is much less time-consuming and more practical in daily practices. |
| Question 8: | | | | | | | | |
| 2201 (6 Clinical Trial, 2 Cohort) | Not serious | Not serious | Serious | Not serious | Not serious | ⨁⨁◯◯  LOW | \ | A number of clinical trials have employed MASES as the endpoint to assess the efficacy of treatment of enthesitis. Results showed that following treatment, enthesitis showed improvements as indicated by MASES. |

**Table 8: Studies addressing Question 5**

| Study | Year | Design | Population | Entheses | Abnormal | Percentage |
| --- | --- | --- | --- | --- | --- | --- |
| Fiorenza[21] | 2020 | Cross-sectional | 39 PsA | 390 | 167 | 0.43 |
| Macchioni[22] | 2019 | Cross-sectional | 140 PsA | 1960 | 453 | 0.23 |
| Zhang[23] | 2017 | Cohort | 20 AS | 240 | 47 | 0.20 |
| Michelsen[24] | 2017 | Cross-sectional | 141 PsA | 282 | 88 | 0.31 |
| Althoff[25] | 2016 | Cohort | 41 SpA | 697 | 85 | 0.12 |
| Poggenborg[26] | 2013 | Cross-sectional | 18 SpA | 321 | 79 | 0.25 |
| Bandinelli[27] | 2013 | Cross-sectional | 92 PsA | 920 | 57 | 0.06 |
| Spadaro[28] | 2011 | Cross-sectional | 36 AS | 432 | 64 | 0.15 |
| Ruta[29] | 2011 | Cross-sectional | 60 SpA | 600 | 56 | 0.09 |
| Laatiris[12] | 2010 | Cross-sectional | 76 AS | 2584 | 925 | 0.36 |
| Klauser[20] | 2008 | Cohort | 21 pts of suspected multiple enthesitis | 273 | 75 | 0.27 |
| Genc[30] | 2005 | Cross-sectional | 18 AS | 252 | 63 | 0.25 |
| Balint[31] | 2002 | Cross-sectional | 35 SpA | 350 | 71 | 0.20 |


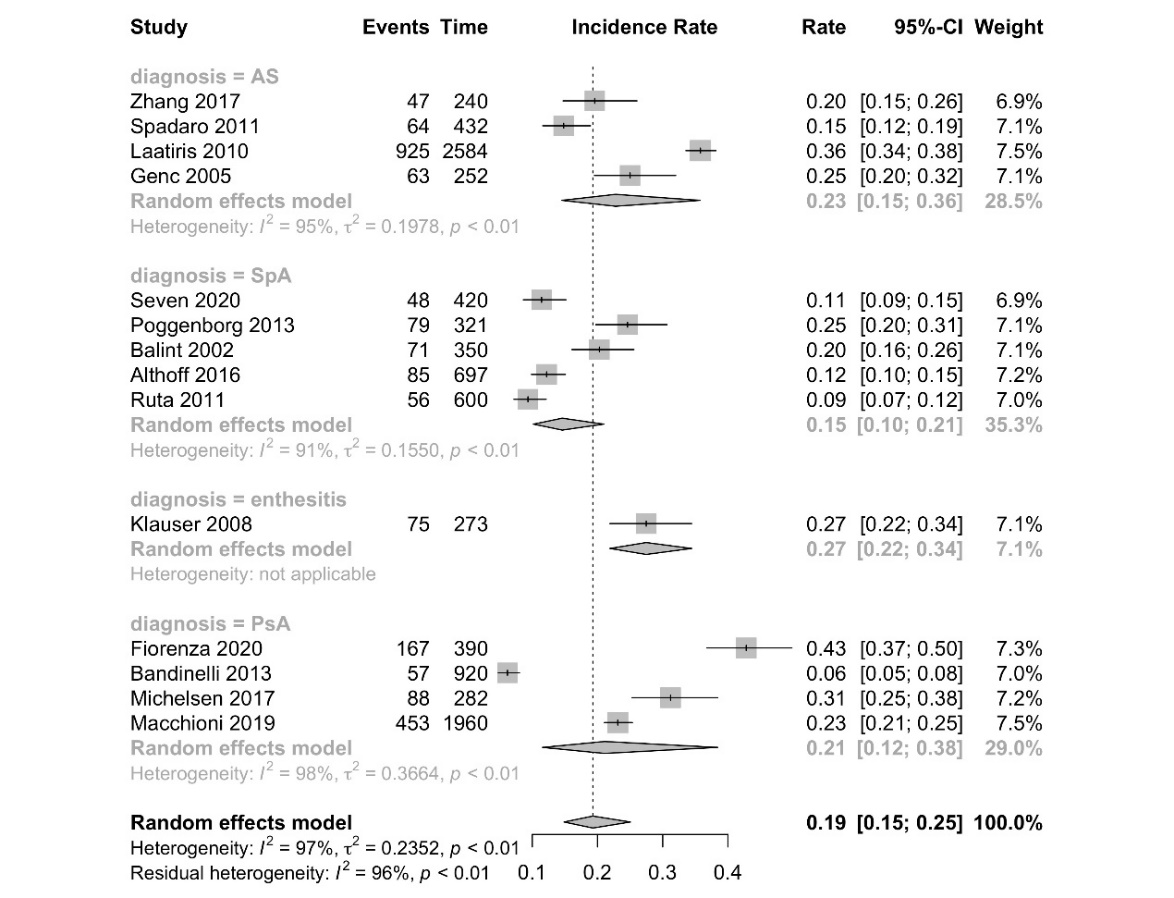


**Figure 1: Forest Plot of Positive Rate: enthesitis identified by physical examination in patients with SpA**

**Table 9: Studies addressing Question 6**

| Study | Year | Design | Population | Enthesis | Item | Reference | sensitivity | specificity |
| --- | --- | --- | --- | --- | --- | --- | --- | --- |
| Zhang[23] | 2017 | Cohort | 20 AS | peripheral entheses | PE | US (with vascularization) | 0.16 | 0.86 |
|  |  |  |  |  |  | US (without vascularization) | 0.23 | 0.85 |
| Wiell[32] | 2012 | Cross-sectional | 12 SpA | heel | PE | contrast-enhanced MRI | 0.58 | 0.84 |
| Spadaro[28] | 2011 | Cross-sectional | 23 AS | peripheral entheses | PE | US (with vascularization) | 0.23 | 0.85 |
|  |  |  |  |  |  | US (without vascularization) | 0.16 | 0.86 |
| Klauser[20] | 2008 | Cohort | 21 pts of suspected multiple enthesitis | peripheral entheses | MASES | US | 0.71-0.87 | 0.47-0.75 |
| Balint[31] | 2002 | Cross-sectional | 35 SpA | lower limbs | PE | US | 0.226 | 0.797 |

**Table 10: Studies addressing Question 7**

| Study | Year | Design | Population | Result |
| --- | --- | --- | --- | --- |
| Heuft-Dorenbosch[33] | 2003 | Cross-sectional | 217 AS | The MASES (range 0–13) has much greater feasibility than the MEI (range 0–90). However, up to 21% of patients with a score >0 on the MEI were not identified by a score on the MASES >0. Only 2.1% of the patients with an original enthesis score >0 had an original score on the MEI >3 (range 0–90) and it can be questioned whether a low score on the MEI index represents clinically important enthesitis. The Spearman correlation coefficient between the MASES score and the MEI was 0.90 and between the MASES and the BASDAI was 0.53 compared with a correlation of 0.59 between the MEI and the BASDAI. |
| Healy[34] | 2008 | Cohort | 60 PsA | Tenderness at the 6 points of MEI was detected in 80% of patients with an MEI scored＞0, suggesting that a positive score with this combination is likely to be sensitive enough to monitor entheses that are tender. |
| Hamdi[35] | 2011 | Cross-sectional | 28 AS | The SPARCC correlated closely with the MASES (P= 0.0001), although the two indices share only four entheseal sites. |

**Table 11: Studies addressing Question 8**

| Study | Year | Design | Polulation | Treatment | Duration | Score | Baseline mean | Baseline SD | Follow-up mean | Follow-up SD | p | Indicator |
| --- | --- | --- | --- | --- | --- | --- | --- | --- | --- | --- | --- | --- |
| Seven[36] | 2020 | Clinical trial | 11 SpA | adalimumab | 24weeks | MASES | 3 | 3.7 | 2 | 3 | nr | yes |
|  |  |  |  |  |  | LEI | 1.1 | 1.1 | 0.3 | 0.7 | nr |  |
|  |  |  |  |  |  | SPARCC-EI | 2.6 | 2.9 | 0.8 | 1.1 | p＜0.05 |  |
| Lee[37] | 2020 | Clinical trial | 201 AS | adalimumab | 12weeks | MASES | 2.67 | 1.88 | 0.85 | 1.86 | P＜0.0001 | yes |
| Gladman[38] | 2018 | Clinical trial | 327 PsA | apremilast | 24weeks | MASES | 4.5 | 3.2 | 3.2 | nr | nr | no |
| Hartung[39] | 2018 | Cohort | 145 SpA | DMARD  / Prednisone  / iTNF | 3months | MASES | 5.57 | 3.37 | 3.7 | 3.28 | p＜0.001 | yes |
| Van der Heijde[40] | 2013 | Clinical trial | 140 AS | golimumab | 14weeks | MASES | 3.8 | 3.36 | 2.5 | 3.11 | nr | yes |
|  |  |  |  |  |  | Berlin EI | 2.7 | 2.9 | 2.0 | 2.47 | p＜0.05 |  |
|  |  |  |  |  |  | University of California San Francisco EI | 4.6 | 4.03 | 3.1 | 3.54 | p＜0.01 |  |
| Rudwaleit[41] | 2010 | Clinical trial | 667 AS | adalimumab | 12weeks | MASES | 5 | 4.44 | 1 | 2.96 | nr | no |
| Healy[34] | 2008 | Cohort | 28 PsA | Methotrexate  / leflunomide  / etanercept  / hydroxychloroquine | 3months | MEI | 11.6 | 7.6 | 7.2 | 6.5 | p＜0.05 | yes |
|  |  |  |  |  |  | MASES | 4.9 | 3.9 | 2.2 | 2.1 | p＜0.01 |  |
|  |  |  |  |  |  | Leeds EI | 2.9 | 1.7 | 1.9 | 1.7 | p＜0.05 |  |
|  |  |  |  |  |  | Gladman indix | 2.5 | 1.6 | 0.6 | 0.8 | p＜0.01 |  |
|  |  |  |  |  |  | Major index | 3.9 | 2.2 | 2.6 | 2.1 | p＜0.01 |  |

**PART 4：ULTRASOUND (Question 9-17)**

**Question 9: The frequency of enthesitis identified by physical examination or ultrasound in patients with Spondyloarthritis**

We found 2 Cohort and 2 Cross-sectional studies addressing this question, which included 135 participants.

The evidence shows:

-It was consistently reported that ultrasound examination could detect significantly higher number of enthesitic lesions than physical examination.

-It could be inferred that subclinical enthesitis could be present in a significant portion of SpA patients.

-US is a promising approach to the detection of subclinical enthesitic lesions.

The quality of evidence is MODERATE.

**Question 10: The sensitivity and specificity of ultrasound to identify enthesitis in patients with Spondyloarthritis**

We found 2 Cohort and 6 Cross-sectional studies addressing this question, which included 955 participants.

The evidence shows:

-The sensitivity of abnormal findings of entheses upon ultrasound examination varied across different studies, depending on the diagnosis and the definition of abnormal findings.

-Comprehensive scoring systems, such as MASEI or GUESS, could yield a sensitivity between 50% to 90%.

-Specificity of ultrasound examination is consistently reported to be around 80%.

The quality of evidence is LOW.

**Question 11: The inter-observer and intra-observer coefficients of ultrasound to identify enthesitis in patients with Spondyloarthritis**

We found 5 Cohort and 3 Cross-sectional studies addressing this question, which included 699 participants.

The evidence shows:

-Despite concerns expressed in earlier studies regarding the reliability of US, it has been proved that US is a reliable tool in the assessment of enthesitis.

-Multiple studies reported a stellar intra-class correlation coefficient (ICC) of US, which exceeded 0.9 according to most of the studies.

-Either the intra-observer and the inter-observer ICC of US is excellent.

The quality of evidence is LOW.

**Question 12: The enthesis lesion under ultrasound screening in patients with Spondyloarthritis**

We found 1 Clinical trial, 4 Cohort and 10 Cross-sectional studies addressing this question, which included 1255 participants.

The evidence shows:

-Pathologies of enthesitis under ultrasound examination include signs of acute inflammation and chronic enthesitis.

-Signs of acute inflammation include edema, thickening, vascularization and bursitis, while chronic lesions include calcification, erosion and enthesophytes.

-Occurrence rates of edema, thickening, bursitis and PD signal at each enthesis in SpA patients were 10%, 16%, 12% and 8%, respectively.

-Occurrence rates of calcification, erosion and enthesophytes at each enthesis in SpA patients were 15%, 18% and 12%, respectively.

The quality of evidence is HIGH.

**Question 13: The vascularization of enthesis in patients with Spondyloarthritis**

We found 1 Cohort and 6 Cross-sectional studies addressing this question, which included 619 participants.

The evidence shows:

-Vascularization of the entheses is significantly more common in SpA/AS patients than in controls.

-Rarely seen in healthy individuals, PD signal can be considered a very specific sign to SpA/AS.

-Power doppler ultrasound (PDUS) is an important tool in the visualization of vascularized entheses.

The quality of evidence is HIGH.

**Question 14: The distribution of enthesitis under ultrasound screening in patients with Spondyloarthritis**

We found 1 Clinical trial, 3 Cohort and 3 Cross-sectional studies addressing this question, which included 551 participants.

The evidence shows:

-Occurrence rates of enthesitis at lateral epicondyle, medial epicondyle, greater trochanter, quadriceps tendon, patellar ligament, Achilles tendon and plantar fascia were 30%, 7%, 30%, 38%, 42%, 39% and 21%, respectively.

-Enthesitis is more common in the lower limbs, especially at the knee and Achilles tendons.

The quality of evidence is LOW.

**Question 15: The MASEI score in patients with Spondyloarthritis**

We found 5 Case-control studies addressing this question, which included 484 participants.

The evidence shows:

-The MASEI score is significantly higher in SpA/AS patients than in healthy controls. (SMD=9.88, 95%CI = [7.37, 12.40])

-MASEI is a reliable quantitative tool in the ultrasound assessment of enthesitis.

-MASEI evaluates enthesis thickness, bursitis, PD signal, structure, calcification and erosion.

-Compared with GUESS, MASEI incorporated PDUS as one of the scoring items, providing an opportunity of visualizing vascularized entheses.

The quality of evidence is MODERATE.

**Question 16: The correlation between ultrasound enthesitis score and disease activity score in patients with Spondyloarthritis**

We found 7 Cohort and 6 Cross-sectional studies addressing this question, which included 1740 participants.

The evidence shows:

-The majority of the studies failed to identify an association between US enthesitis score and disease activity status.

-Enthesitis might be an independent indicator not subject to systemic inflammation.

The quality of evidence is LOW.

**Question 17: The change of ultrasound enthesitis score after treatment in patients with Spondyloarthritis**

We found 1 Clinical trial and 7 Cohort studies addressing this question, which included 749 participants.

The evidence shows:

-Despite the variety of indices employed as the endpoints, ultrasound examination is widely used in clinical trials to assess the improvements of enthesitis.

-Common US scoring systems include MASEI, GUESS, SEI and D’agostino grading system.

-Results showed that following treatment, enthesitis significantly improved as indicated by the US indices.

-US is capable of reflecting the therapeutic responses of enthesitis.

The quality of evidence is LOW.

**Table 12: Evidence profile**

| Certainty assessment | | | | | | | Summary of findings | |
| --- | --- | --- | --- | --- | --- | --- | --- | --- |
| No of participants  (studies)  Follow-up | Risk of bias | Inconsistency | Indirectness | Imprecision | Publication bias | Overall certainty of evidence | Pooled Result (95%CI) | Brief Summary |
| Question 9: | | | | | | | | |
| 135 (2 Cohort, 2 Cross-sectional) | Serious | Not serious | Not serious | Not serious | Not serious | ⨁⨁⨁◯  MODERATE | 3.22  (2.33, 4.45) | It was consistently reported that ultrasound examination could detect significantly higher number of enthesitis lesions than physical examination. |
| Question 10: | | | | | | | | |
| 955 (2 Cohort, 6 Cross-sectional) | Serious | Serious | Serious | Serious | Not serious | ⨁⨁◯◯  LOW | \ | The sensitivity of abnormal findings of entheses upon ultrasound examination varied across different studies, depending on the diagnosis and the definition of abnormal findings, approximately 50%-90%. Specificity was approximately 80%. |
| Question 11: | | | | | | | | |
| 699 (5 Cohort, 3 Cross-sectional) | Serious | Serious | Serious | Serious | Not serious | ⨁⨁◯◯  LOW | \ | The ultrasound examination is a reliable tool in the assessment of enthesitis, as indicated by its stellar intra-class correlation coefficient (ICC), which exceeded 0.9 according to most of the studies. |
| Question 12: | | | | | | | | |
| 1255  (1 Clinical trial, 4 Cohort, 10 Cross-sectional) | Not serious | Not serious | Not serious | Not serious | Not serious | ⨁⨁⨁⨁  HIGH | \ | Pathologies of enthesitis under ultrasound examination include signs of acute inflammation and chronic enthesitis. Signs of acute inflammation include edema, thickening, vascularization and bursitis, while chronic lesions include calcification, erosion and enthesophytes. Occurrence rates of each pathology differ from 10% to 20%. |
| Question 13: | | | | | | | | |
| SpA | | | | | | | | Vascularization of the entheses is significantly more common in SpA/AS than in controls. Rarely seen in healthy individuals, PD signal can be considered a very specific sign to SpA/AS. |
| 609 (1 Cohort, 6 Cross-sectional) | Not serious | Not serious | Not serious | Not serious | Not serious | ⨁⨁⨁⨁  HIGH | 6.45  (18.9, 22.04) |  |
| SpA vs. HC | | | | | | | |  |
| 172 (3 Cross-sectional) | Not serious | Not serious | Not serious | Not serious | Not serious | ⨁⨁⨁⨁  HIGH | 6.10  (1.14, 32.80) |  |
| SpA vs. non-SpA | | | | | | | |  |
| 459 (1 Cohort, 4 Cross-sectional) | Not serious | Not serious | Not serious | Not serious | Not serious | ⨁⨁⨁⨁  HIGH | 4.40  (0.79, 24.50) |  |
| Question 14: | | | | | | | | |
| 551  (1 Clinical trial, 3 Cohort, 3 Cross-sectional) | Not serious | Not serious | Not serious | Not serious | Not serious | ⨁⨁⨁⨁  HIGH | \ | Occurrence rates of enthesitis at lateral epicondyle, medial epicondyle, greater trochanter, quadriceps tendon, patellar ligament, Achilles tendon and plantar fascia were 30%, 7%, 30%, 38%, 42%, 39% and 21%, respectively. Enthesitis is more common in the lower limbs. |
| Question 15: | | | | | | | | |
| 424 (5 Cross-sectional) | Not Serious | Not Serious | Not Serious | Not Serious | Not Serious | ⨁⨁⨁◯  MODERATE | 9.88  (7.37, 12.40) | The MASEI score is significantly higher in SpA/AS patients than in healthy controls. MASEI is a reliable quantitative tool in the ultrasound assessment of enthesitis. |
| Question 16: | | | | | | | | |
| 1740  (7 Cohort, 6 Cross-sectional) | Serious | Serious | Serious | Serious | Not Serious | ⨁⨁◯◯  LOW | \ | The majority of the studies failed to identify an association between US enthesitis score and disease activity status. Enthesitis might be an independent indicator not subject to systemic inflammation. |
| Question 17: | | | | | | | | |
| 749  (1 Clinical trial, 7 Cohort) | Serious | Serious | Serious | Serious | Not Serious | ⨁⨁◯◯  LOW | \ | Despite the variety of indices employed as the endpoints, ultrasound examination is widely used in clinical trials to assess the improvements of enthesitis. Results showed that following treatment, enthesitis significantly improved as indicated by the US indices. |

**Table 13: Studies addressing Question 9**

| Study | Year | Design | Population | Sites | Enthesitis identified by physical examination | Enthesitis identified by ultrasound |
| --- | --- | --- | --- | --- | --- | --- |
| Zhang[23] | 2017 | Cohort | 20 AS | 240 | 47 | 123 |
| Spadaro[28] | 2011 | Cross-sectional | 36 AS | 432 | 36 | 192 |
| Alcalde[42] | 2007 | Cohort | 44 AS | 440 | 35 | 109 |
| Balint[31] | 2002 | Cross-sectional | 35 AS | 348 | 75 | 195 |


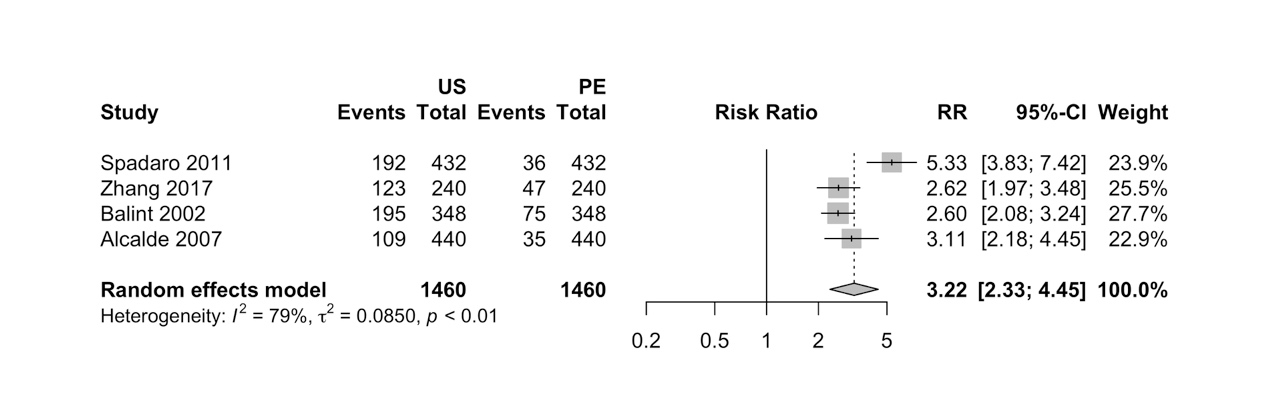


**Figure 2: Forest Plot of comparison: enthesitis identified by ultrasound vs. physical examination in patients with SpA**

**Table 14: Studies addressing Question 10**

| Study | Year | Design | Population | Enthesis | Item | Reference | Sensitivity | Specificity |
| --- | --- | --- | --- | --- | --- | --- | --- | --- |
| Ozsoy-unubol[43] | 2018 | Cross-sectional | 30 nr-axSpA | peripheral entheses | GUESS | ASAS | 0.967 | 0.8 |
|  |  |  |  |  | MASEI | ASAS | 0.933 | 0.8 |
| Poulain[1] | 2018 | Cohort | 402 recent-onset IBP | peripheral entheses | PDUS enthesitis | ASAS | 0.139 | 0.835 |
| Aydin[44] | 2014 | Cross-sectional | 55 SpA | heel | achilles thickness > 4.8mm in male | ESSG | 0.43 | 0.85 |
|  |  |  |  |  | achilles thickness > 3.7mm in female | ESSG | 0.7 | 0.59 |
| Feydy[45] | 2011 | Cross-sectional | 51 SpA | heel | PDUS | Amor | 0.59 | 0.87 |
| Hu[46] | 2011 | Cross-sectional | 161 AS | SIJ + peripheral entheses | CDUS | AS | 0.522 | 0.729 |
| D'Agostino[3] | 2011 | Cohort | 118 SpA | peripheral entheses | vascularized enthesis | SpA | 0.765 | 0.812 |
| de Miguel[4] | 2011 | Cross-sectional | 113 early  spondyloarthritis | peripheral entheses | MASEI>20 | SpA | 0.5575 | 0.8947 |
| de Miguel[5] | 2009 | Cross-sectional | 25 SpA | peripheral entheses | MASEI>18 | ESSG | 0.833 | 0.828 |

**Table 15: Studies addressing Question 11**

| Study | Year | Design | Population | Score | Class | ICC | 95%CI | |
| --- | --- | --- | --- | --- | --- | --- | --- | --- |
| Ishida[47] | 2019 | Cross-sectional | 50 SpA | Brachial triceps thickness | inter-observer | 0.968 | 0.909 | 0.989 |
|  |  |  |  | Distal quadriceps thickness | inter-observer | 0.971 | 0.917 | 0.99 |
|  |  |  |  | Proximal patellar tendon thickness | inter-observer | 0.987 | 0.961 | 0.995 |
|  |  |  |  | Distal patellar tendon thickness | inter-observer | 0.975 | 0.789 | 0.994 |
|  |  |  |  | Achilles tendon thickness | inter-observer | 0.99 | 0.97 | 0.997 |
|  |  |  |  | plantar fascia thickness | inter-observer | 0.97 | 0.916 | 0.99 |
| Ozsoy-unubol[43] | 2018 | Cross-sectional | 30 nr-axSpA | MASEI | intra-observer | 0.975 | 0.898 | 0.994 |
| Ruta[48] | 2014 | Cohort | 34 SpA | global score | intra-observer | 0.977 | 0.961 | 0.993 |
| Falcao[49] | 2014 | Cohort | 146 SpA | MASEI | intra-observer | 0.77 | 0.2 | 0.95 |
| Aydin[50] | 2010 | Cohort | 43 AS | achilles thickness | intra-observer | 0.88-0.92 | 0.85 | 0.93 |
| Naredo[51] | 2010 | Cohort | 327 SpA (ESSG) | Morphologic abnormalities score | intra-observer | 0.95 | 0.93 | 0.958 |
|  |  |  |  | Calcific deposit score | intra-observer | 0.96 | 0.948 | 0.068 |
|  |  |  |  | Cortical abnormalities score | intra-observer | 0.97 | 0.966 | 0.979 |
|  |  |  |  | Adjacent bursitis score | intra-observer | 0.955 | 0.936 | 0.961 |
|  |  |  |  | Intraenthesis power Doppler score | intra-observer | 0.98 | 0.979 | 0.987 |
|  |  |  |  | Perienthesis power Doppler score | intra-observer | 0.98 | 0.977 | 0.986 |
| de Miguel[5] | 2009 | Cross-sectional | 25 SpA (ESSG) | MASEI | inter-observer | 0.6 | 0.42 | 0.76 |
| Alcalde[42] | 2007 | Cohort | 44 AS | SEI | inter-observer | 0.72 | 0.56 | 0.83 |

**Table 16: Studies addressing Question 12 (Achilles tendon)**

| Study | Year | Design | Population | Calcification | | Erosion | | Enthesophyte | | Edema | | Thickening | | Bursitis | | PD signal | |
| --- | --- | --- | --- | --- | --- | --- | --- | --- | --- | --- | --- | --- | --- | --- | --- | --- | --- |
| Seven[36] | 2020 | Clinical trial | 21 axSpA | 42 | 28 | 42 | 3 | - | - | - | - | 42 | 6 | - | - | 42 | 0 |
| Ishida[47] | 2019 | Cross-sectional | 50 AS | 100 | 48 | 100 | 17 | - | - | 100 | 17 | 100 | 36 | 100 | 10 | 100 | 6 |
| Ruyssen-Witrand[15] | 2017 | Cohort | 402 SpA | 804 | 32 | 804 | 30 | 402 | 110 | - | - | 804 | 51 | 804 | 52 | 804 | 17 |
| Zhang[23] | 2017 | Cohort | 20 AS | 40 | 3 | 40 | 2 | - | - | 40 | 5 | 40 | 11 | 40 | 11 | 40 | 15 |
| Harman[52] | 2017 | Cross-sectional | 40 axSpA | 80 | 66 | 80 | 72 | - | - | - | - | 80 | 22 | 80 | 8 | 80 | 4 |
| Baraliakos[53] | 2017 | Cross-sectional | 30 pSpA | 37 | 5 | 37 | 22 | - | - | - | - | - | - | - | - | 37 | 12 |
| Wink[54] | 2017 | Cohort | 111 AS | 222 | 8 | 222 | 10 | 222 | 67 | 222 | 0 | 222 | 2 | 222 | 14 | 222 | 5 |
| Sudol-Szopinska[55] | 2014 | Cross-sectional | 30 enthesitis | - | - | 30 | 8 | 30 | 21 | - | - | 30 | 0 | - | - | - | - |
| Wiell[32] | 2012 | Cross-sectional | 12 SpA | 24 | 6 | 24 | 2 | 24 | 21 | 24 | 6 | 24 | 6 | 24 | 5 | 24 | 2 |
| Hamdi[56] | 2011 | Cross-sectional | 60 AS | - | - | 120 | 115 | 120 | 100 | 120 | 24 | - | - | - | - | - | - |
| Spadaro[28] | 2011 | Cross-sectional | 36 AS | 72 | 5 | 72 | 3 | 72 | 35 | 72 | 10 | 72 | 20 | 72 | 10 | 72 | 6 |
| Feydy[45] | 2011 | Cross-sectional | 51 AS | 94 | 3 | 94 | 6 | 94 | 12 | - | - | 94 | 14 | 94 | 3 | 94 | 5 |
| Naredo[51] | 2010 | Cohort | 327 SpA | 394 | 67 | - | - | - | - | - | - | - | - | 394 | 69 | 394 | 61 |
| Kiris[57] | 2006 | Cross-sectional | 30 AS | 60 | 2 | - | - | - | - | 60 | 6 | - | - | 60 | 23 | 60 | 12 |
| Balint[31] | 2002 | Cross-sectional | 35 SpA | - | - | 70 | 9 | 70 | 21 | - | - | 70 | 14 | 70 | 7 | - | - |


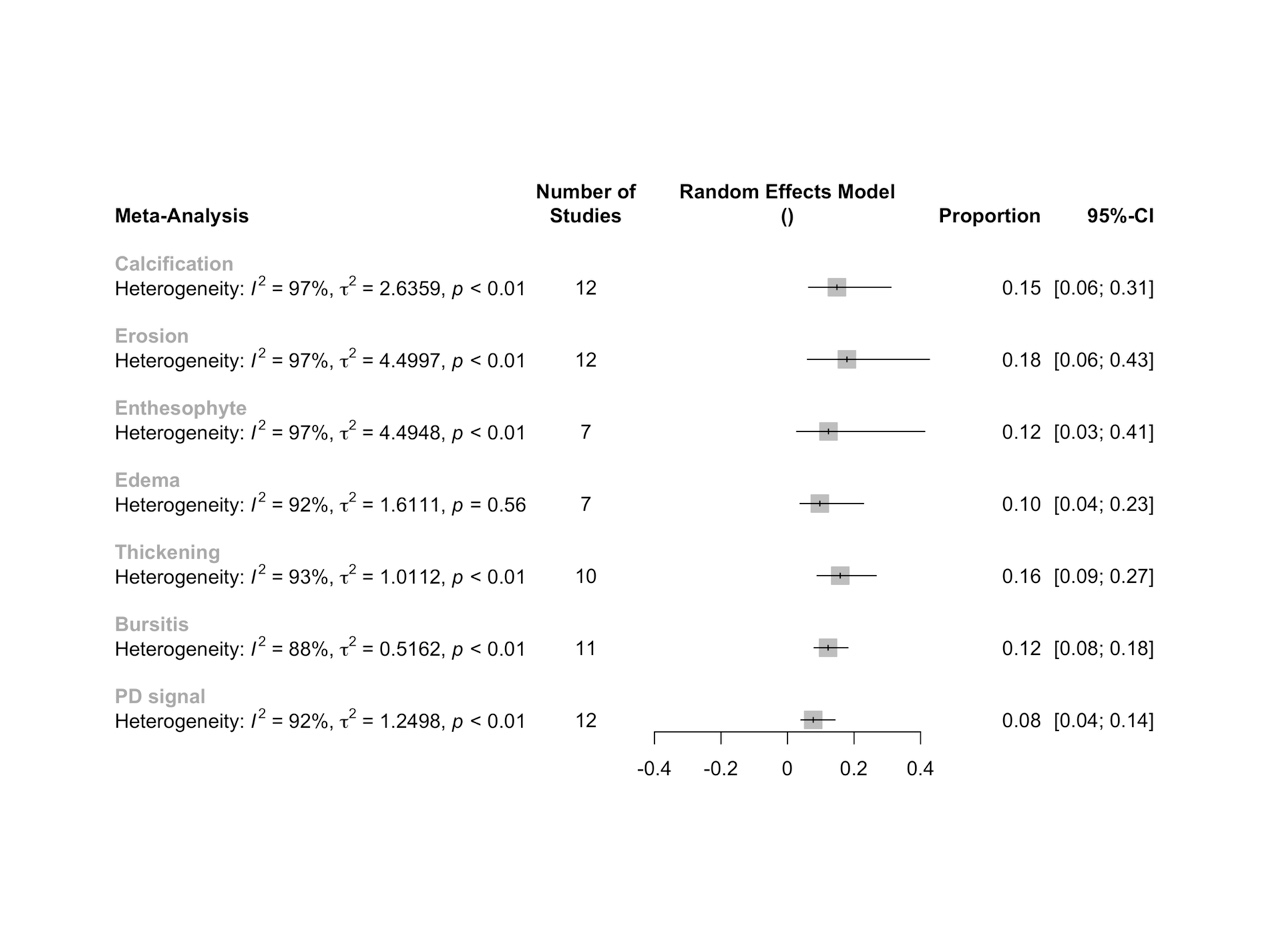


**Figure 3: Forest Plot: ultrasound signs of enthesitis in Achilles tendon**

**Table 17: Studies addressing Question 12 (plantar fascia)**

| Study | Year | Design | Population | Calcification | | Erosion | | Enthesophyte | | Edema | | Thickening | | Bursitis | | PD signal | |
| --- | --- | --- | --- | --- | --- | --- | --- | --- | --- | --- | --- | --- | --- | --- | --- | --- | --- |
| Seven[36] | 2020 | Clinical trial | 21 axSpA | 40 | 0 | 40 | 1 | - | - | 40 | 4 | 40 | 6 | - | - | 40 | 0 |
| Ishida[47] | 2019 | Cross-sectional | 50 AS | 100 | 31 | 100 | 7 | - | - | 100 | 27 | 100 | 38 | - | - | 100 | 0 |
| Zhang[23] | 2017 | Cohort | 20 AS | 40 | 4 | 40 | 0 | - | - | - | - | 40 | 5 | 40 | 0 | 40 | 6 |
| Baraliakos[53] | 2017 | Cross-sectional | 30 pSpA | 37 | 5 | 37 | 2 | - | - | 72 | 7 | - | - | - | - | 37 | 1 |
| Wink[54] | 2017 | Cohort | 111 AS | 222 | 0 | 222 | 3 | 222 | 12 | 222 | 0 | 222 | 0 | 222 | 0 | 222 | 1 |
| Spadaro[28] | 2011 | Cross-sectional | 36 AS | 72 | 7 | 72 | 0 | 72 | 5 | - | - | 72 | 10 | 72 | 0 | 72 | 0 |
| Hamdi[56] | 2011 | Cross-sectional | 60 AS | - | - | 120 | 18 | 120 | 91 | 120 | 36 | - | - | - | - | - | - |
| Feydy[45] | 2011 | Cross-sectional | 51 AS | 94 | 0 | 94 | 4 | 94 | 22 | - | - | 94 | 4 |  |  | 94 | 0 |
| Naredo[51] | 2010 | Cohort | 327 SpA | 394 | 10 | - | - | - | - | - | - | - | - | 10 | 1 | 394 | 5 |
| Balint[31] | 2002 | Cross-sectional | 35 SpA | - | - | 70 | 6 | 70 | 4 | - | - | 70 | 35 | - | - | - | - |


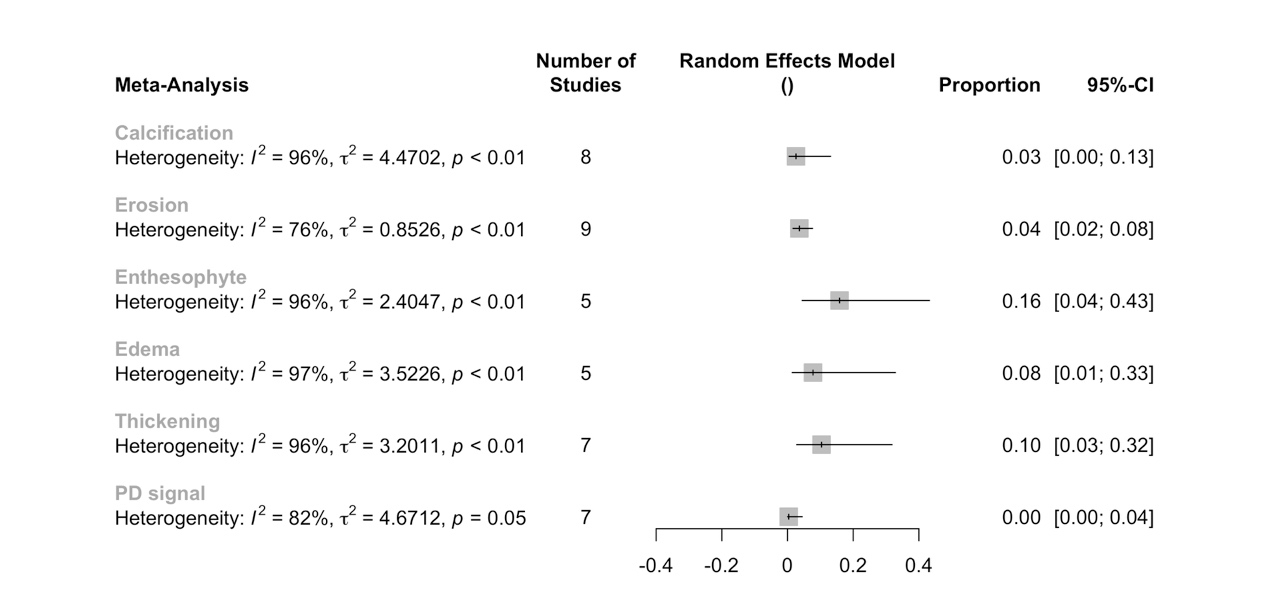


**Figure 4: Forest Plot: ultrasound signs of enthesitis in Plantar fascia**

**Table 18: Studies addressing Question 12 (Quadriceps)**

| Study | Year | Design | Population | Calcification | | Erosion | | Enthesophyte | | Edema | | Thickening | | Bursitis | | PD signal | |
| --- | --- | --- | --- | --- | --- | --- | --- | --- | --- | --- | --- | --- | --- | --- | --- | --- | --- |
| Seven[36] | 2020 | Ckinial trial | 21 axSpA | 42 | 20 | 42 | 3 | - | - | - | - | - | - | - | - | - | - |
| Ishida[47] | 2019 | Cross-sectional | 50 AS | 100 | 41 | 100 | 2 | - | - | 100 | 14 | 100 | 45 | - | - | 100 | 1 |
| Zhang[23] | 2017 | Cohort | 20 AS | 40 | 17 | 40 | 1 | 40 | 2 | 40 | 20 | 40 | 11 | 40 | 1 | 40 | 8 |
| Spadaro[28] | 2011 | Cross-sectional | 36 AS | 72 | 32 | 72 | 1 | 72 | 43 | 72 | 37 | 72 | 21 | 72 | 1 | 72 | 1 |
| Hamdi[56] | 2011 | Cross-sectional | 60 AS | - | - | 120 | 90 | 120 | 112 | - | - | 120 | 58 | - | - | - | - |
| Naredo[51] | 2010 | Cohort | 327 SpA | 394 | 65 | - | - | - | - | - | - | - | - | 394 | 12 | 394 | 49 |
| Balint[31] | 2002 | Cross-sectional | 35 SpA | - | - | 70 | 5 | 70 | 8 | - | - | 70 | 25 | 70 | 9 | - | - |


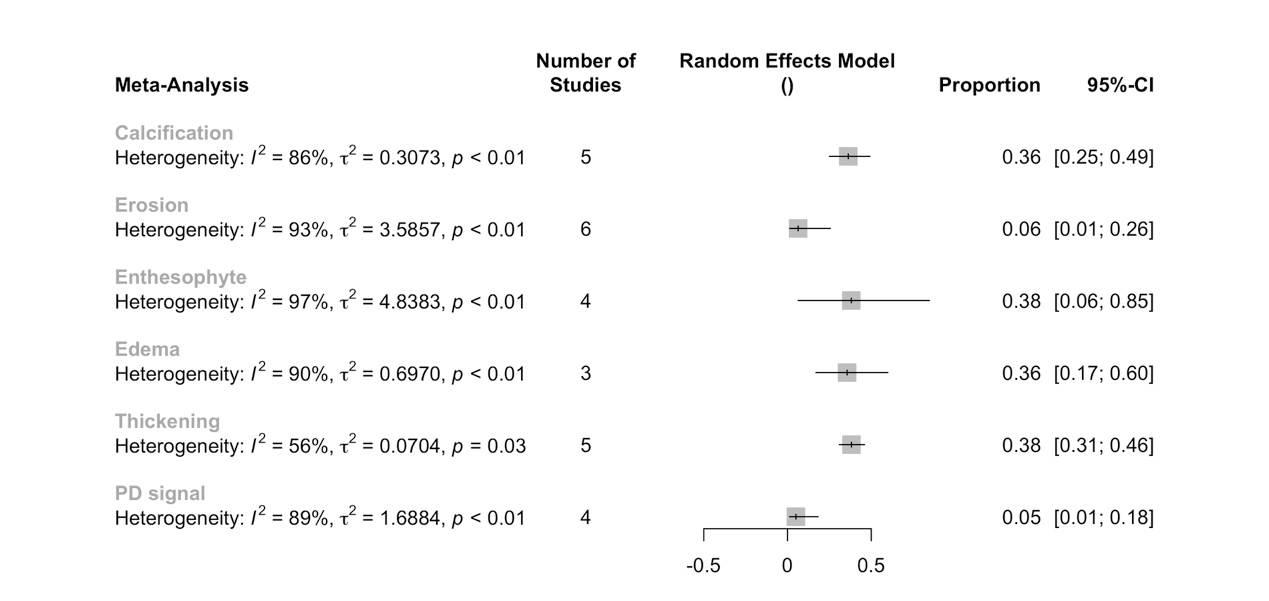


**Figure 5: Forest Plot: ultrasound signs of enthesitis in Quadriceps**

**Table 19: Studies addressing Question 12 (Tibial tuberosity)**

| Study | Year | Design | Population | Calcification | | Erosion | | Enthesophyte | | Edema | | Thickening | | Bursitis | | PD signal | |
| --- | --- | --- | --- | --- | --- | --- | --- | --- | --- | --- | --- | --- | --- | --- | --- | --- | --- |
| Seven[36] | 2020 | Clinical trial | 21 axSpA | 42 | 3 | 42 | 0 | - | - | - | - | - | - | - | - | - | - |
| Ishida[47] | 2019 | Cross-sectional | 50 AS | 100 | 12 | 100 | 2 | - | - | 100 | 8 | 100 | 69 | 100 | 6 | 100 | 1 |
| Ruyssen-Witrand[15] | 2017 | Cohort | 402 SpA | 804 | 37 | 804 | 17 | 804 | 16 | - |  | 804 | 30 | 804 | 0 | 804 | 27 |
| Zhang[23] | 2017 | Cohort | 20 AS | 40 | 10 | 40 | 3 | 40 | 9 | 40 | 9 | 40 | 13 | 40 | 5 | 40 | 12 |
| Baraliakos[53] | 2017 | Cross-sectional | 30 pSpA | 13 | 0 | 13 | 1 | 13 | 0 | - | - | - | - | - | - | 13 | 4 |
| Wink[54] | 2017 | Cohort | 111 AS | 222 | 1 | 222 | 1 | 222 | 3 | 222 | 0 | 222 | 0 | 222 | 1 | 222 | 12 |
| Spadaro[28] | 2011 | Cross-sectional | 36 AS | 72 | 18 | 72 | 6 | 72 | 23 | 72 | 16 | 72 | 23 | 72 | 21 | 72 | 4 |
| Hamdi[56] | 2011 | Cross-sectional | 60 AS | - | - | 120 | 107 | 120 | 83 | - | - | 120 | 78 | - | - | - | - |
| Naredo[51] | 2010 | Cohort | 327 SpA | 394 | 24 | - | - | - | - | - | - | - | - | 394 | 92 | 394 | 52 |
| Balint[31] | 2002 | Cross-sectional | 35 SpA | - | - | - | - | 69 | 1 | 70 | 2 | 70 | 3 | 70 | 32 | - | - |


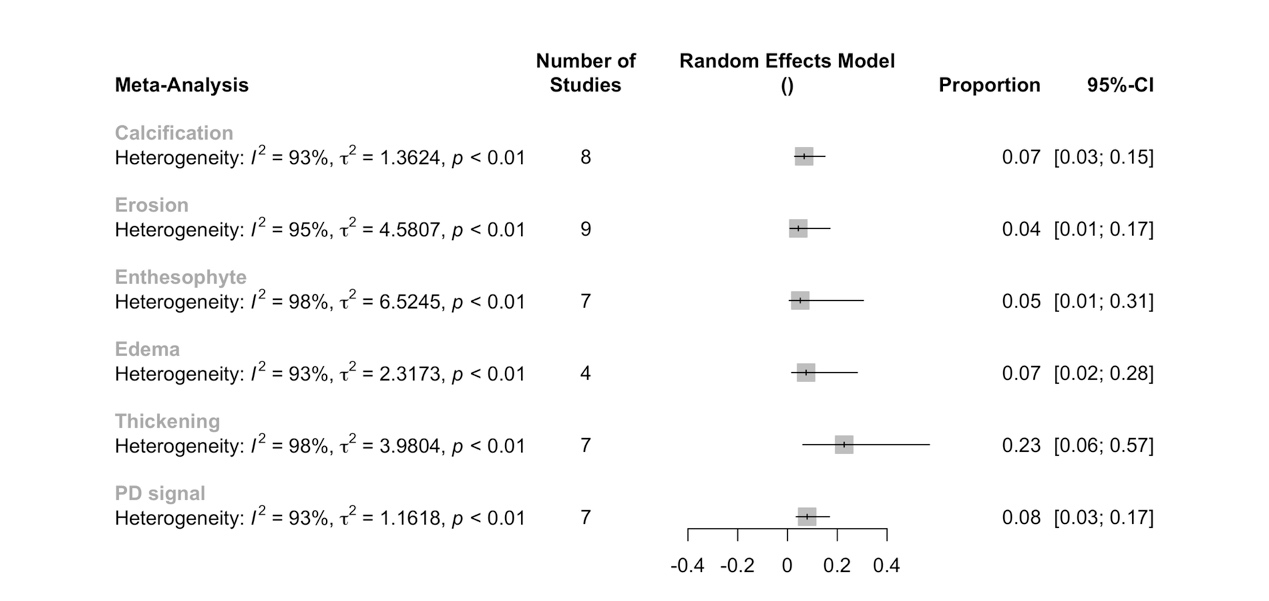


**Figure 6: Forest Plot: ultrasound signs of enthesitis in Tibial tuberosity**

**Table 20: Studies addressing Question 13**

| Study | Year | Design | Sites | Patients | Tendons of patients | Vascularization of patients | Controls | Tendons of controls | Vascularization of controls |
| --- | --- | --- | --- | --- | --- | --- | --- | --- | --- |
| Ishida[47] | 2019 | Cross-sectional | achilles tendon | 50 AS | 100 | 6 | 30 HC | 60 | 0 |
| Ozsoy-Unubol[43] | 2018 | Cross-sectional | all sites | 30 nr-axSpA | 60 | 8 | 30 non-SpA | 60 | 0 |
| Harman[52] | 2017 | Cross-sectional | achilles tendon | 40 axSpA | 80 | 4 | 30HC | 60 | 0 |
| Wiell[32] | 2012 | Cross-sectional | achilles tendon | 12 SpA | 24 | 2 | 15 non-SpA | 30 | 4 |
|  |  |  |  |  |  |  | 10 HC | 20 | 0 |
| D'Agostino[3] | 2011 | Cohort | all sites | 51 SpA | 714 | 83 | 48 non-SpA | 672 | 13 |
| Feydy[45] | 2011 | Cross-sectional | achilles tendon | 51 SpA | 94 | 5 | 24 non-SpA | 32 | 2 |
| D'Agostino[58] | 2003 | Cross-sectional | all sites | 164 SpA | 1131 | 916 | 34 non-SpA | 59 | 0 |


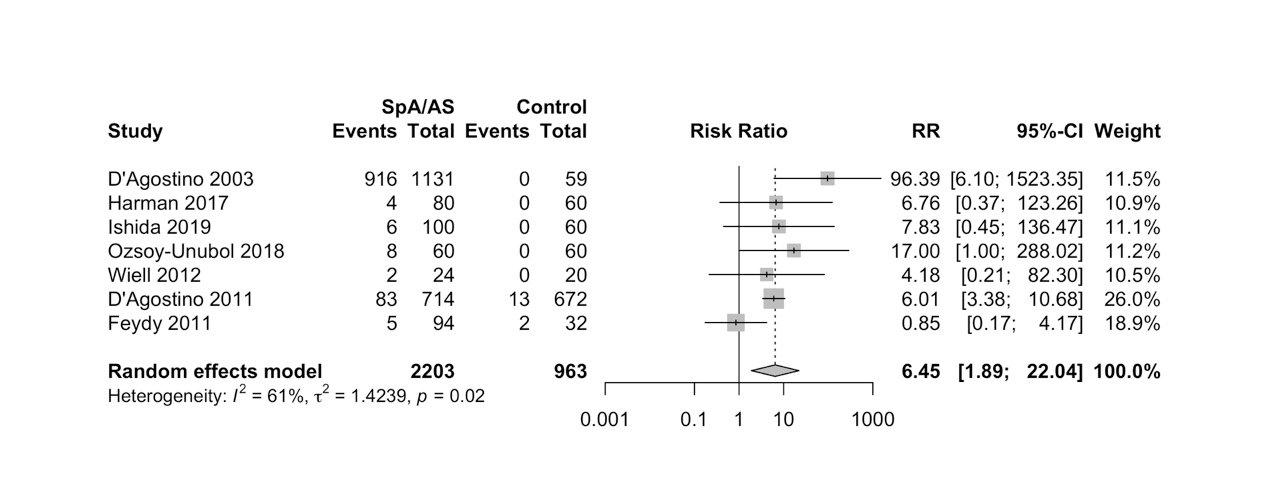


**Figure 7: Forest Plot:** **Risk ratio of enthesis vascularization in SpA**


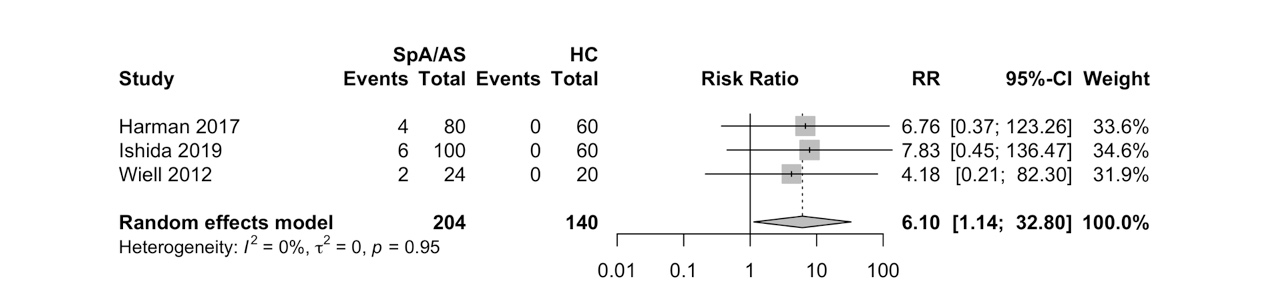


**Figure 8: Forest Plot:** **Risk ratio of enthesis vascularization in SpA vs. HC**


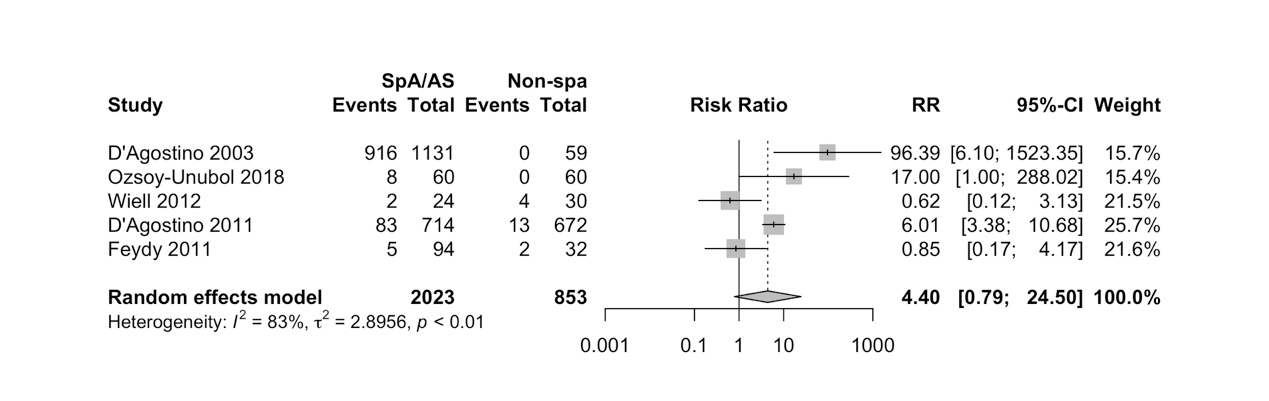


**Figure 9: Forest Plot:** **Risk ratio of enthesis vascularization in SpA vs. non-SpA**

**Table 21: Studies addressing Question 14**

| Study | Year | Design | Population | No of enthesis | No of enthesitis | | | | | | |
| --- | --- | --- | --- | --- | --- | --- | --- | --- | --- | --- | --- |
|  |  |  |  |  | Lateral epicondyle | Medial epicondyle | Greater trochanter | Quadriceps tendon | Patellar tendon | Achilles tendon | Plantar tendon |
| Seven[36] | 2020 | Clinical trial | 21 axSpA | 378 | 5 | 0 | 11 | 13 | 3 | 17 | 4 |
| Ozsoy-Unubol[43] | 2018 | Cross-sectional | 30 nr-axSpA | 1080 | 12 | 2 | - | 21 | 31 | 24 | 21 |
| Zhang[23] | 2017 | Cohort | 20 AS | 240 | 18 | - | 23 | 24 | 28 | 20 | 10 |
| Wink[54] | 2017 | Cohort | 111 AS | 1998 | 39 | 13 | 19 | 43 | 42 | 21 | 3 |
| Spadaro[28] | 2011 | Cross-sectional | 36 AS | 432 | 42 | - | 34 | 46 | 31 | 26 | 13 |
| D'Agostino[3] | 2011 | Cohort | 51 SpA | 714 | 43 | 20 | 14 | 37 | 61 | 35 | 35 |
| D'Agostino[58] | 2003 | Cross-sectional | 164 SpA | 2952 | 80 | 82 | 143 | 91 | 193 | 260 | 243 |
|  |  |  | 104 AS | 1872 | 55 | 52 | 98 | 61 | 133 | 163 | 152 |


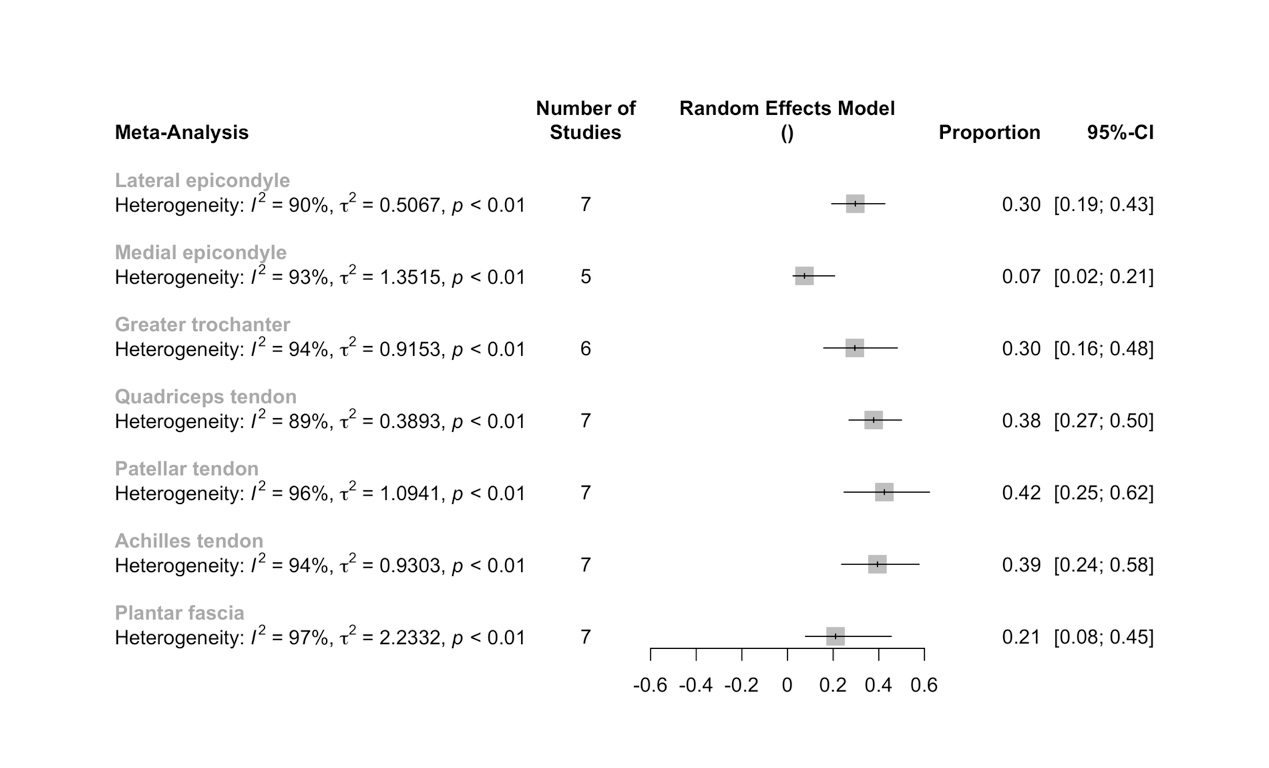


**Figure 10: Forest Plot: Distribution of enthesitis under ultrasound screening in patients with SpA**

**Table 22: Studies addressing Question 15**

| Study | Year | Design | Population | MASEI of patients (mean±SD) | MASEI of controls (mean±SD) |
| --- | --- | --- | --- | --- | --- |
| Ishida[47] | 2019 | Cross-sectional | 50 AS, 30 HC | 16.32±11.22 | 10.7±5.27 |
| Ozsoy-unubol[43] | 2018 | Cross-sectional | 30 nr-axSpA, 30 HC | 10.57±5.54 | 2.33±3.38 |
| Lanfranchi[59] | 2017 | Cross-sectional | 30 axSpA, 30 HC | 26.3±13 | 12.2±7 |
| de Miguel[4] | 2011 | Cross-sectional | 113 SpA, 57 HC | 23.36±11.4 | 12.26±6.85 |
| de Miguel[5] | 2009 | Cross-sectional | 25 SpA, 29 HC | 25.44±7.92 | 12.96±7.84 |


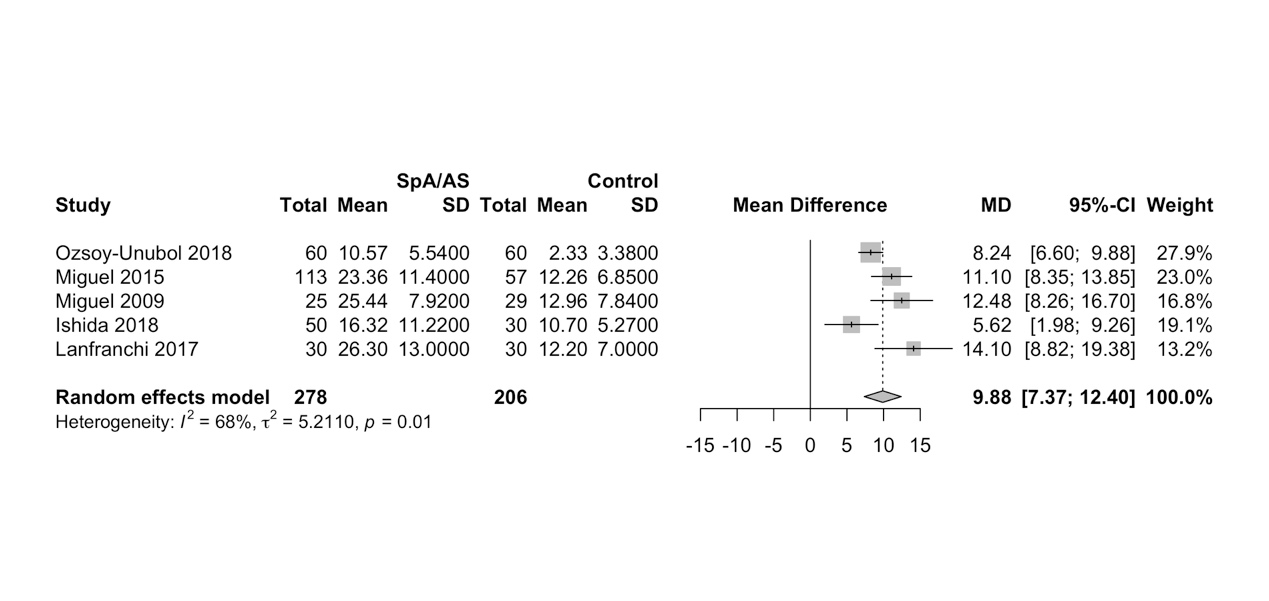


**Figure 11: Forest Plot: Distribution of enthesitis under ultrasound screening in patients with SpA**

**Table 23: Studies addressing Question 16**

| Study | Year | Design | Population | Enthesis | Score | ESR | CRP | ASDAS | BASDAI | BASFI | BASMI | BASRI |
| --- | --- | --- | --- | --- | --- | --- | --- | --- | --- | --- | --- | --- |
| Ozsoy-unubol[43] | 2018 | Cross-sectional | 60 nr-axSpA | peripheral entheses | MASEI | NR | NR | r=0.376, p=0.041 | NR | NR | NR | - |
|  |  |  |  |  | GUESS | NR | NR | NR | NR | NR | NR | - |
| Ruyssen-Witrand[15] | 2017 | Cohort | 708 IBP | peripheral entheses | US structural score | - | NR | NR | NR | NR | NR | - |
|  |  |  |  |  | PDUS score | - | NR | NR | r=0.075, p=0.2 | NR | NR | - |
| Harman[52] | 2017 | Cross-sectional | 40 SpA | heel | Achilles enthesitis total score | NR | NR | - | r = 0.523, p = 0.001 | r = 0.533, p = 0.001 | - | - |
| Lanfranchi[59] | 2017 | Cross-sectional | 164 SpA (ASAS) | peripheral entheses | MASEI | - | - | NR | NR | - | - | - |
| Wink[54] | 2017 | Cohort | 111 AS | peripheral entheses | change in number of inflammatory lesions | NR | NR | NR | NR | NR | - | - |
| Wang[60] | 2015 | Cohort | 100 AS | peripheral entheses | Reduction in total score | NR | NR | - | r = 0.3935, P < 0.0001 | NR | NR | - |
| Ruta[48] | 2014 | Cohort | 34 SpA (ASAS) | peripheral entheses + joints + tendons | Global ultrasound assessment | r=0.3126 | r=0.3111 | - | r=0.0526 | - | - | - |
| Naredo[51] | 2010 | Cohort | 327 SpA (ESSG) | peripheral entheses | elementary lesion score | NR | NR | - | NR | NR | - | - |
|  |  |  |  |  | changes in the elementary lesion score | NR | NR | - | NR | NR | - | - |
| Aydin[50] | 2010 | Cohort | 43 AS | heel | improvement in total score | r= 0.679,  P < 0.001 | NR | - | NR | - | - | - |
| Alcalde[42] | 2007 | Cohort | 44 AS | lower limbs | SEI-A | - | - | NR | NR | - | - | - |
|  |  |  |  |  | SEI-C | - | - | - | - | NR | NR |  |
|  |  |  |  |  | SEI | - | - | - | - | - | - | - |
| Kiris[57] | 2006 | Cross-sectional | 30 AS | axial entheses + achilles tendon | power Doppler score | NR | NR | - | NR | NR | - | - |
| Borman[61] | 2005 | Cross-sectional | 44 SpA (ESSG) | Foot | R GUESS | NR | NR | - | NR | - | NR | NR |
|  |  |  |  |  | L GUESS | NR | NR | - | NR | - | NR | NR |
| Balint[31] | 2002 | Cross-sectional | 35 SpA (ESSG) | lower limbs | GUESS | NR | NR | - | - | - | - | - |

**Table 24: Studies addressing Question 17**

| Study | Year | Design | Population | Treatment | Duration | Score | Baseline mean | Baseline SD | Follow-up mean | Follow-up SD | P | Treatment indicator |
| --- | --- | --- | --- | --- | --- | --- | --- | --- | --- | --- | --- | --- |
| Seven[36] | 2020 | Clinical trial | 49 axSpA | Adalimumab | 24 weeks | GUESS | 3.18 | 1.6 | 2.56 | 1.81 | >0.05 | No |
|  |  |  |  |  |  | SEI | 1.55 | 2.16 | 0.89 | 1.05 | >0.05 |  |
|  |  |  |  |  |  | MASEI | 7.36 | 4.46 | 6.89 | 5.13 | >0.05 |  |
| Hartung[39] | 2018 | Cohort | 65 SpA (AS+PsA) | csDMARDs/bDMARDs | 6 months | PDUS score | 5.47 | 3.97 | 2.88 | 3.61 | <0.001 | Yes |
| Zhang[23] | 2017 | Cohort | 20 AS | Etanercept | 3 months | PDUS score | 106 | (77-136) | 37 | (29-63) | <0.01 | Yes |
| Wink[54] | 2017 | Cohort | 111 AS | TNFi | 6 months | number of inflammatory entheseal lesions | 210 |  | 180 |  | 0.2 | No |
| Wang[60] | 2015 | Cohort | 25 AS | infliximab | 3 months | TS | 9.96 | 9.676 | 3.64 | 4.071 | 0.0005 | Yes |
|  |  |  | 25 AS | etanercept | 3 months | TS | 11.44 | 10.96 | 4.32 | 4.432 | 0.0011 |  |
|  |  |  | 25 AS | adalimumab | 3 months | TS | 11 | 9.055 | 3.4 | 3.651 | 0.0002 |  |
|  |  |  | 25 AS | non-biologic | 3 months | TS | 10.6 | 10.46 | 7.2 | 7.194 | 0.8497 |  |
| Ruta[48] | 2014 | Cohort | 34 SpA | csDMARDs/bDMARDs | 3 months | GSUS  (Soft tissues abnormalities) | 3.88 | (2.8-4.96) | 1.14 | (0.6-1.68) | <0.0001 | Yes |
|  |  |  |  |  |  | GSUS  (Cortical bone changes) | 4.52 | (3.33-5.72) | 3.76 | (2.73-4.79) | 0.1754 |  |
|  |  |  |  |  |  | PDUS | 1.47 | (0.84-2.09) | 0.29 | (-0.03-0.62) | <0.0001 |  |
|  |  |  |  |  |  | Total score | 9.88 | (7.73-12.03) | 5.2 | (3.93-6.47) | <0.0001 |  |
| Naredo[51] | 2010 | Cohort | 327 SpA (ESSG) | TNFi | 6 months | Morphologic abnormalities score | 2.19 | 2.66 | 1.34 | 2.02 | <.0005 | Yes |
|  |  |  |  |  |  | Calcific deposit score | 1.11 | 0.163 | 1.23 | 1.79 | 0.142 |  |
|  |  |  |  |  |  | Cortical abnormalities score | 3.92 | 3.73 | 4.17 | 3.86 | 0.036 |  |
|  |  |  |  |  |  | Adjacent bursitis score | 0.94 | 1.21 | 0.76 | 1.19 | 0.036 |  |
|  |  |  |  |  |  | Intraenthesis power Doppler score | 1.36 | 2.11 | 0.68 | 1.64 | <.0005 |  |
|  |  |  |  |  |  | Perienthesis power Doppler score | 1.75 | 2.92 | 0.98 | 2.23 | <.0005 |  |
| Aydin[50] | 2010 | Cohort | 43 AS | TNFi | 2 months | GS | 3.6 | 3 | 2.3 | 2.2 | <0.001 | Yes |
|  |  |  |  |  |  | PD | 1 | 2.4 | 0.5 | 1.6 | 0.067 |  |
|  |  |  |  |  |  | TS | 4.7 | 4.9 | 2.7 | 3.3 | <0.001 |  |

**PART 5：MRI (Question 18-22)**

**Question 18: The value of MRI in the assessment of enthesitis in patients with spondyloarthritis**

We found 8 Cohort, 3 Case-control and 1 Cross-sectional study addressing this question, which included 1020 participants.

The evidence shows:

-The foremost strength of MRI in the detection of enthesitis is its high resolution.

-MRI is the only modality capable of visualizing osteitis at the entheses.

-MRI examination is relatively expensive and not easily accessible.

-Conventional MRI examination could not provide a head-to-toe image in one scan. It is impractical to scan multiple locations.

The quality of evidence is VERY LOW.

**Question 19: The value of whole-body MRI in the assessment of enthesitis in patients with spondyloarthritis**

We found 7 Cross-sectional, 3 Cohort studies and 3 RCT addressing this question, which included 591 participants.

The evidence shows:

-Several clinical trials have employed whole-body MRI to assess the efficacy of treatment.

-A scoring system named MRI-WIPE was developed to evaluate the whole-body MRI scan.

-There is some concern regarding the readability of whole-body MRI, especially at the distal limbs.

-Each whole-body MRI scan takes approximately one hour, while the evaluation process takes another hour. It is time-consuming and impractical in daily practices.

The quality of evidence is LOW.

**Question 20: The value of UTE sequence of MRI in the assessment of enthesitis in patients with spondyloarthritis**

We found 1 Animal experiment, 2 Cadaveric analysis and 1 Cross-sectional study addressing this question, which included 16 participants.

The evidence shows:

-UTE is a promising novel MRI sequence in the detection of enthesitis, with the unique strength of depicting the structure of entheses more clearly.

-Its foremost limitation is that it is still not commercialized on a large scale.

The quality of evidence is VERY LOW.

**Question 21: The value of contrast-enhanced MRI in the assessment of enthesitis in patients with spondyloarthritis**

We found 3 Cross-sectional and 2 Cohort studies addressing this question, which included 351 participants.

The evidence shows:

-At the peripheral entheses, the administration of contrast agents could help identify a small number of extra enthesitic lesions, approximately 10% more.

-At the entheses of the pelvis, contrast-enhanced MRI could visualize more enthesitic lesions, but the extra findings do not bear incremental diagnostic value.

-Evidence on this topic is very limited. The necessity of contrast agents is still debated.

The quality of evidence is VERY LOW.

**Question 22: The value of HEMRIS score in the assessment of ankle enthesitis in patients with spondyloarthritis**

We found 2 Cross-sectional studies and 1 Atlas addressing this question, which included 48 participants.

The evidence shows:

-HEMRIS is a novel scoring system recently proposed to evaluate enthesitis at the Achilles tendon.

-This scoring system is composed of inflammatory parameters (intra-tendon hypersignal on T2w/STIR sequences, peri-tendon hypersignal, bone marrow edema and bursitis) and structural scores (enthesophyte, bone erosion, tendon thickening).

-Its reliability was validated by a few studies, but its potential in evaluating enthesitis at other entheses awaits further validation.

The quality of evidence is VERY LOW.

**Table 25: Evidence profile**

| Certainty assessment | | | | | | | Summary of findings | |
| --- | --- | --- | --- | --- | --- | --- | --- | --- |
| No of participants  (studies)  Follow-up | Risk of bias | Inconsistency | Indirectness | Imprecision | Publication bias | Overall certainty of evidence | Pooled Result (95%CI) | Brief Summary |
| Question 18: | | | | | | | | |
| 1020 (8 Cohort, 3 Case-control, 1 Cross-sectional) | Serious | Serious | Serious | Serious | Not serious | ⨁◯◯◯  VERY LOW | \ | MRI is capable of depicting the entheses with relatively high resolution as well as presenting osteitis. However, conventional MRI techniques could not provide a head-to-toe image in one scan, apart from the fact that MRI is costly, time-consuming and not easily accessible. |
| Question 19: | | | | | | | | |
| 591 (7 Cross-sectional, 3 Cohort, 3 RCT) | Serious | Not serious | Not serious | Serious | Not serious | ⨁⨁◯◯  LOW | \ | Several clinical trials have employed whole-body MRI to assess the efficacy of treatment, and even devised a scoring system based on whole-body MRI. However, evidence showed that readability, especially in the distal limbs, was still less than satisfactory. It is also time-consuming and not easily accessible. |
| Question 20: | | | | | | | | |
| 16 (1 Animal experiment, 2 Cadaveric analysis, 1 Cross-sectional) | Serious | Not serious | Serious | Serious | Not serious | ⨁◯◯◯  VERY LOW | \ | UTE is a promising novel MRI sequence in the detection of enthesitis, with the unique strength of depicting the structure of entheses more clearly. Its foremost limitation is that it is still not commercialized on a large scale. |
| Question 21: | | | | | | | | |
| 351 (3 Cross-sectional, 2 Cohort) | Serious | Serious | Serious | Serious | Not serious | ⨁◯◯◯  VERY LOW | \ | Few studies have ever investigated the value of contrast-enhanced MRI in the detection of peripheral enthesitis. The administration of contrast agents could help identify a small number of extra enthesitis lesions, but its necessity is still debated. |
| Question 22: | | | | | | | | |
| 48 (2 Cross-sectional, 1 Atlas) | Serious | Serious | Serious | Serious | Not serious | ⨁◯◯◯  VERY LOW | \ | HEMRIS is a novel scoring system recently proposed to evaluate enthesitis at the Achilles tendon. It was validated by a few studies, but its potential in evaluating enthesitis at other entheses awaits further validation. |

**Table 26: Studies addressing Question 18**

| Study | Year | Design | Population | Result |
| --- | --- | --- | --- | --- |
| Kleinrensink[62] | 2020 | Cohort | 13 Pso, 13 PsA, 12 AS | In clinically unaffected entheses,the Heel Enthesitis MRI Scoring model (HEMRIS) abnormalities occurred in 44/70 (63%) of Achilles tendons and in 23/70 (33%) of plantar fascia. |
| Renson[63] | 2020 | Cohort | 60 pSpA | With regard to enthesitis at baseline, we observed MRI abnormalities in 91% of the clinically involved entheses of the heel: 100% of the Achilles tendon entheses and 80% of the plantar fascia entheses showed concordant entheseal STI and/or osteitis on MRI. Neither clinically involved quadriceps femoris tendon entheses or superior patellar ligament entheses showed MRI abnormalities. In addition, in 30 entheses, without tenderness at baseline clinical examination, STI and/or osteitis was detected on MRI: 11 plantar fascia, eight Achilles tendon, five inferior patellar ligament, four superior patellar ligament, and two quadriceps femoris tendon entheses. |
| Aguila Maldonado[64] | 2017 | Cross-sectional | 40 SpA | The unweighted kappa values between US and MRI were 0.80, 0.66, 0.69, 0.70, and 0.70 for thickening, structural changes, bursitis, enthesophytes, and bone erosions, respectively. The present study provides evidence about the high overall agreement between US and MRI for all abnormal comparable findings at entheseal level. |
| Lorenzin[65] | 2016 | Cohort | 60 SpA | 51.6% of patients showed bone marrow edema (BME) in spine-MRI and 56.7% of patients in SIJ-MRI. Signs of enthesitis were found in 35 (58.3%) patients: at the level of the cervical spine in 5% of the patients, of the thoracic spine in 55% of the patients, at the lumbar spine in 6% of the patients. |
| Herregods[66] | 2015 | Cohort | 143 enthesitis-related arthritis (ERA) | MRI showed pelvic enthesitis in 21 % of patients with ERA and in 13 % of patients without ERA. Pelvic enthesitis was seen on MRI in 7/51 (14 %) patients with clinically evident enthesitis, and 16/92 (17 %) patients without clinically evident enthesitis. In 7 of 11 ERA-negative patients without clinical enthesitis but with pelvic enthesitis on MRI, the ILAR criteria could have been fulfilled, if pelvic enthesitis on MRI was included in the criteria. |
| Klang[67] | 2014 | Cohort | 67 enthesitis | A consensus reading of two senior radiologists was regarded as the gold standard. Gold standard analysis diagnosed 83 (36 %) enthesitis lesions. Intra-reader reliability for the experiencedreader was significantly (p=0.0001) higher in the T1+Gd images compared to the STIR images (hamstring: k=0.84/0.45, GM: k=0.84/0.47). Sensitivity and specificity increased from 0.74/0.8 to 0.87/0.9 in the STIR images and T1+Gd sequences. Intra-reader reliability for the inexperienced reader was lower (p>0.05). |
| Aydin[68] | 2013 | Cohort | 21 SpA | Considering all MRI findings only increased signal in the surrounding tissues was higher at tender sites (41 vs. 20%, p=0.01) and the insertions points themselves showed little abnormality. The positive agreements between individual lesions by both methods was very low (10-26%) with low kappa values (0.06-0.18) with no correlations between the MRI and US scores (r²= 0.059). |
| Jans[69] | 2013 | Cohort | 444 SpA | MRI showed pelvic enthesitis in 24.4%ofpatients with SpA and in 7.1 % of patients without SpA. Presence of any enthesitis had sensitivity, specificity, LR+, LR−, PPVandNPVof 24.4 %, 92.9 %, 3.45, 0.81, 69.4 % and 65.2 % for the diagnosis ofSpA, respectively. |
| Paramarta[70] | 2013 | Case-control | 13 SpA, 20 RA | MRI showed similar prevalence of perientheseal fluid/oedema (67% vs 75%), perientheseal bone marrow oedema (0% vs 10%) and entheseal enhancement (46% vs 47%) in SpA versus RA, respectively. The number and distribution of affected entheseal sites were not different between both diseases. Enthesitis on MRI is not a specific feature of peripheral arthritis in recent onset SpA versus RA. |
| Wiell[32] | 2012 | Case-control | 12 SpA, 15 non-SpA | When MRI was considered gold standard, US showed higher sensitivity for intratendinous and entheseal changes than clinical examination (median sensitivity 0.83 versus 0.66). |
| Emad[71] | 2009 | Case-control | 25 oligoarticular-undifferentiated arthritis (UA), 15 RA, 15 SpA | Enthesitis was found in all patients in the SpA group (100%) and differed from RA and UA groups (p < 0.001). Enthesitis is a common feature on MRI in SpA, while absent in the RA and UA groups. |
| Kamel[72] | 2002 | Cohort | 32 SpA | MRI showed tendon enlargement (62.5%) with loss of the normal flattened hypointense appearance, focal thickening and rounded configuration at the insertion site (31.2%), intermediate T1 and high T2 signals, and diminished signals within the pre-Achilles fat pad due to inflammatory edema. MRI was not sensitive compared to US in detecting early changes of enthesopathy. Fatty degeneration appeared late in MRI, while it was detected earlier using US. MRI was not able to detect any calcification process at the insertion site, while US images clearly showed the very early signs of the calcification process. |

**Table 27: Studies addressing Question 19**

| Study | Year | Design | Population | Result |
| --- | --- | --- | --- | --- |
| Krabbe[73] | 2020 | Cohort | 53 axSpA  ADA (48 weeks) | This study proposed novel criteria for treatment response and remission in patients with axSpA as assessed by whole-body magnetic resonance imaging (WB-MRI) of axial and peripheral joints and entheses. WB-MRI demonstrated a significant reduction of inflammation in both the spine, sacroiliac joints and peripheral joints and entheses during golimumab treatment. |
| Poulsen[74] | 2020 | Cross-sectional | 14 PsA  10 RA  16 HC | WBMRI showed good interscan agreement, implying that repositioning of the patient between examinations does not markedly affect scoring of lesions. Intra- and interreader agreement was moderate to almost perfect. |
| Krabbe[75] | 2019 | RCT | 49 axSpA  ADA (48 weeks) | The most frequent “MRI positive” entheses were greater femoral trochanter (21%), calcaneal Achilles tendon insertion (17%) and ischial tuberosity (16%). The agreement between WB-MRI enthesitis and tenderness of the individual joints, agreement was poor with kappa values site by site <0.4. Kappa value of inter-rater agreement for enthesitis was 0.59. |
| Krabbe[76] | 2019 | Cross-sectional | 4 RA  4 PsA | This study developed a whole-body MRI-scoring system (MRI-WIPE) for peripheral arthritis and enthesitis. Reliability was moderate-good for musculoskeletal radiologists and rheumatologists |
| Krabbe[77] | 2018 | RCT | 49 axSpA  ADA (48 weeks) | The WBMRI enthesis inflammation index decreased significantly in the ADA group (mean change –0.9) compared with the placebo group (+0.4) at Week 6. |
| Althoff[25] | 2016 | Cohort | 41 axSpA  ETN (3 years) | Enthesitis at baseline was detected more frequently by standardized clinical examination compared with WB-MRI (57% vs 21% of patients), with a predominance of lesions in the anterior chest wall and pelvis. An overall reduction of enthesitis after 2 and 3 years of therapy was evident both clinically and on WB-MRI. |
| Poggenborg[78] | 2014 | Cross-sectional | 18 PsA  18 axSpA  12 controls | Distal peripheral joints generally showed lower readability than more proximal joints. |
| Poggenborg[26] | 2014 | Cross-sectional | 18 PsA  18 axSpA  12 HC | WBMRI allowed evaluation of 888 (53%) of 1680 sites investigated, and 19 (54%) of 35 entheses had a readability >70%. The percentage agreement between WBMRI and clinical enthesitis was 49–100%. Enthesitis on WBMRI was observed in 148 (17%) of the entheseal sites, and was frequently present at greater trochanters (55%) and Achilles (43%) and supraspinate (23%) tendon insertions in patients and HS. The readability of the MRIs varied substantially, from very high for entheses at pelvis, shoulder and hip to very low for entheses at elbow, knee and foot. |
| Karpitschka[79] | 2013 | Cohort | 10 AS  ETN (52 weeks) | WBMRI detected areas of synovitis and enthesitis with a sensitivity of 90 % compared to a sensitivity of clinical examination alone of 20 %. During ETN therapy, the enthesitis score decreased from Week 0 (3.3 ± 1.2) to Week 52 (0.2 ± 0.2). |
| Althoff[80] | 2012 | Cross-sectional | 39 AS  36 nr-axSpA | Enthesitis was significantly more common in the AS group (n=22) than in the nr-axSpA group (n=12; p=0.0449). Inflammatory signs at ligament and tendon insertions were found most frequently at the pelvis. Multilocular enthesitis was significantly more common in the AS group than in the nr-axSpA group (p=0.0133). |
| Weber[81] | 2012 | Cross-sectional | 122 axSpA  75 HC | WB MRI signs of ACW inflammation were found in a substantial proportion of patients with AS (49.5%) and nr-SpA (25.9%). There was no association between clinical assessments of ACW, including the MASES, and MRI features. |
| Song[82] | 2011 | RCT | 76 axSpA  (40 ETN + 36 SSZ) | The number of enthesitic sites improved significantly from 26 to 11 in the etanercept group versus 24 to 26 in the sulfasalazine group. Enthesitis by MRI was found in 40.8% of patients, a frequency lower than clinically evident enthesitis |
| Weckbach[83] | 2011 | Cross-sectional | 30  PsA | In 24 patients (80%), WB-MRI detected more locations of enthesitis than clinical exam. WB-MRI detected significantly (p < 0.001) more areas of synovitis/enthesitis than clinical exam except for the hands and feet. |

**Table 28: Studies addressing Question 20**

| study | year | Design | Population | Result |
| --- | --- | --- | --- | --- |
| Dallaudière[84] | 2018 | Animal experiment | 12 immunocompetent Wistar male rats | The 3D-UTE sequence identified the anterior fibrocartilage and posterior collagenic areas of Achilles entheses in all cases. Visual analysis and signal intensity measurements distinguished SpA-affected entheses from healthy ones. Neither the normal anatomy of the enthesis nor its pathological pattern could be identified on T2 bSSFP sequences. |
| Chen[85] | 2018 | Cadaveric analysis | 5 fresh cadaveric ankle specimens | High resolution morphological imaging of the Achilles tendon and enthesis can be achieved at 3 T and especially 11.7 T. The 3D UTE Cones sequences also provide quantitative measures of T2*, T1, MTR, and MT modeling of macromolecular proton fractions. |
| Chen[86] | 2018 | Cross-sectional | 9 PsA  7 HC | The 3D-UTE- Cones sequence provided high resolution imaging of entheses and tendons. Cones-T2* and Cones-MTR values were significantly higher for the PsA patients. |
| Du[87] | 2010 | Cadaveric analysis | 4 fresh cadaveric ankle specimens | Ultrashort echo time (UTE) sequences with a minimum TE of 8 μs provide high signal from both tendons and entheses. UTE spectroscopic imaging (UTESI) technique provides quantitative information including T2⁎, chemical shift and resonance frequency shift due to bulk susceptibility effect. |

**Table 29: Studies addressing Question 21**

| Study | Year | Design | Population | Result |
| --- | --- | --- | --- | --- |
| Sung[88] | 2017 | Cross-sectional | 92 axSpA | STIR with T2FS is sufficient to diagnosing spondyloarthropathy, and contrast enhancement is not necessary. T1CE images may have a role in evaluating enthesitis. |
| Klang[67] | 2014 | Cohort | 67 patients with pelvis or hip MRI | Intra-reader reliability for the experienced reader was significantly (p=0.0001) higher in the T1+Gd images compared to the STIR images. Sensitivity and specificity increased from 0.74/0.8 to 0.87/ 0.9 in the STIR images and T1+Gd sequences. |
| de Hooge[89] | 2013 | Cohort | 127 axSpA | Enthesitis are detectable with the administration of Gd. However, they are always observed in the presence of BME. Therefore T1 and STIR sequence alone are sufficient in the MRI assessment that is used for diagnosing patients with early axSpA. |
| Weckbach[83] | 2011 | Cross-sectional | 30  PsA | 27 of 270 pathological findings (10%, among them especially subtle enthesitis) could be detected only after application of contrast media. |
| Maksymowicz[90] | 2010 | Cross-sectional | 35 axSpA | There was no significant difference in the diagnostic value between FSE T2 images and T1 gadolinium-enhanced images with FS in the evaluation of enthesitis. |

**Table 30: Studies addressing Question 22**

| study | year | Design | Population | result |
| --- | --- | --- | --- | --- |
| Kleinrensink[62] | 2020 | Cross-sectional | 13 Pso  13 PsA  12 AS | HEMRIS is a sensitive method for detection of inflammatory and structural disease of enthesitis at the Achilles tendon and plantar fascia. In clinically unaffected entheses, HEMRIS abnormalities occurred in 44/70 (63%) of Achilles tendons and in 23/70 (33%) of plantar fascia. |
| Mathew[91] | 2020 | Atlas | - | This article put forward the atlas of HEMRIS. The presented reference images can be used to guide scoring achilles tendon and plantar fascia enthesitis. |
| Mathew[92] | 2019 | Cross-sectional | 10 ankles  (4 pSpA + 4 mechanical enthesitis+2 HC) | Median pairwise single-measures intra-class correlation coefficients(ICCs; patient-level) for entheseal inflammation status/change scores were 0.83/0.82 for all readers. HEMRIS is reliable among trained readers and promising for clinical trials. |

**PART 6：X-RAY (Question 23)**

**Question 23: The diagnostic value of radiographs in the assessment of enthesitis in patients with spondyloarthritis**

We found 2 Cohort, 2 Case-control and 1 Cross-sectional study addressing this question, which included 2022 participants.

The evidence shows:

-The value of radiographs in the detection of enthesitis is inferior to ultrasound examination and MRI.

-Despite its reliability in the recognition of enthesophytes, its sensitivity is less than satisfactory.

The quality of evidence is VERY LOW.

**Table 31: Evidence profile**

| Certainty assessment | | | | | | | Summary of findings | |
| --- | --- | --- | --- | --- | --- | --- | --- | --- |
| No of participants  (studies)  Follow-up | Risk of bias | Inconsistency | Indirectness | Imprecision | Publication bias | Overall certainty of evidence | Pooled Result (95%CI) | Brief Summary |
| Question 23: | | | | | | | | |
| 2022 (2 Cohort, 2 Case-control, 1 Cross-sectional) | Serious | Serious | Serious | Serious | Not serious | ⨁◯◯◯  VERY LOW | \ | The value of radiographs in the detection of enthesitis is inferior to ultrasound examination and MRI. Despite its reliability in the recognition of enthesophytes, its sensitivity is less than satisfactory. |

**Table 32: Studies addressing Question 23**

| Study | Year | Design | Population | Result |
| --- | --- | --- | --- | --- |
| Kim[93] | 2018 | Cohort | 40 AS | Radiographic changes in bones and soft tissue were found in 36 out of 40 (90%) symptomatic feet compared with 23 out of 40 (57.5%) asymptomatic contralateral feet regardless of the pain location. In group PS (posterior heel pain), 19 (95%) symptomatic feet showed abnormal radiographic changes at the corresponding (posterior) area, whereas 7 out of 20 asymptomatic contralateral feet (35%) showed abnormal radiographic changes (P < 0.001). In group PL (plantar pain), 11 out of 20 symptomatic feet (55%) showed abnormal radiographic changes at the corresponding (plantar) area, whereas 8 out of 20 asymptomatic feet (40%) showed abnormal radiographic changes. |
| Helliwell[94] | 2007 | Case-control | 588 PsA, 536 control (384 RA, 72 AS, 38 undiff. SpA, 32 other inflammatory arthritis) | A significant difference was seen between groups for any entheseal erosion with ankylosing spondylitis having the highest proportion at 18.1%. The differences were mainly due to the proportion of this radiographic feature seen at entheseal sites in the pelvis. Entheseal new bone formation was also uncommon, more often found in ankylosing spondylitis, and in particular the inferior pelvic bones. |
| Taylor[95] | 2003 | Cohort | 62 PsA | The following features showed sufficient reliability to be reasonably included in further testing of their discriminatory value (intra- and interobserver kappa values): entheseal erosion (0.80, 0.71), entheseal ossification (0.69, 0.76), |
| Secundini[96] | 1997 | Cross-sectional | 19 AS, 15 PsA, 6 Reiter's syndrome | When radiologic enthesopathies were classified according to Resnick, 27/40 patients (67.5%) had predominantly the inflammatory type (16 patients with AS and 11 with PsA) with the ischiatic tuberosity being the most frequent location. |
| Resnick[97] | 1977 | Case-control | 64 RA, 40 AS, 26 PsA, 15 Reiter syndrome, 75 control | 90 patients with definite ankylosing spondylitis (Rome Criteria). Of these patients, 40 had radiographs of heels and 15 (38%) had abnormalities. Radiographs of 25 heels in 15 patients were abnormal. The typical radiological features included bony erosions along the posterior aspect of the calcaneus in association with proliferative change. |

**PART 7：PET/CT (Question 24)**

**Question 24: The diagnostic value of PET/CT in the assessment of enthesitis in patients with spondyloarthritis**

We found 3 Cross-sectional studies addressing this question, which included 78 participants.

The evidence shows:

-All the studies failed to link the metabolic uptake on PET/CT to the activity of enthesitis.

-PET/CT is not a reliable tool in the detection of enthesitis.

-PET/CT is expensive and not easily accessible, with ionizing radiation.

The quality of evidence is VERY LOW.

**Table 33: Evidence profile**

| Certainty assessment | | | | | | | Summary of findings | |
| --- | --- | --- | --- | --- | --- | --- | --- | --- |
| No of participants  (studies)  Follow-up | Risk of bias | Inconsistency | Indirectness | Imprecision | Publication bias | Overall certainty of evidence | Pooled Result (95%CI) | Brief Summary |
| Question 24: | | | | | | | | |
| 78 (3 Cross-sectional) | Serious | Serious | Serious | Serious | Not serious | ⨁◯◯◯  VERY LOW | \ | All the studies failed to link the metabolic uptake on PET/CT to the activity of enthesitis. PET/CT is not a reliable tool in the detection of enthesitis. |

**Table 34: Studies addressing Question 24**

| Study | Year | Design | Population | Result |
| --- | --- | --- | --- | --- |
| Kleinrensink[62] | 2020 | Cross-sectional | 13 Pso, 13 PsA,  12 AS | Inflammatory HEMRIS at the Achilles tendon and plantar fascia were not associated with local uptake on PET/CT. The diagnostic value of PET/CT for AS/spondyloarthritis has not been proven. |
| Bruijnen[98] | 2012 | Cross-sectional | 12 AS | This PET/CT study suggested that AS activity is reflected by bone activity (formation) rather than inflammation. This study did not provide data about enthesitis. |
| Vijayant[99] | 2012 | Cross-sectional | 17 RA,  11 axSpA | In axSpA patients, uptake in the affected joint was heterogeneous, low grade and non- symmetrical. No data about enthesitis. |

Reference:

1. Poulain C., D'Agostino M.A., Thibault S., Daures J.P., Ferkal S., Le Corvoisier P., et al. Can power Doppler ultrasound of the entheses help in classifying recent axial spondyloarthritis? Data from the DESIR cohort*.* *RMD Open* 2018;**4**(2):e000686 doi: 10.1136/rmdopen-2018-000686.

2. Eder L., Jayakar J., Thavaneswaran A., Haddad A., Chandran V., Salonen D., et al. Is the MAdrid Sonographic Enthesitis Index useful for differentiating psoriatic arthritis from psoriasis alone and healthy controls? *J Rheumatol* 2014;**41**(3):466-72 doi: 10.3899/jrheum.130949.

3. D'Agostino M.A., Aegerter P., Bechara K., Salliot C., Judet O., Chimenti M.S., et al. How to diagnose spondyloarthritis early? Accuracy of peripheral enthesitis detection by power Doppler ultrasonography*.* *Ann Rheum Dis* 2011;**70**(8):1433-40 doi: 10.1136/ard.2010.138701.

4. de Miguel E., Munoz-Fernandez S., Castillo C., Cobo-Ibanez T., and Martin-Mola E. Diagnostic accuracy of enthesis ultrasound in the diagnosis of early spondyloarthritis*.* *Ann Rheum Dis* 2011;**70**(3):434-9 doi: 10.1136/ard.2010.134965.

5. de Miguel E., Cobo T., Muñoz-Fernández S., Naredo E., Usón J., Acebes J.C., et al. Validity of enthesis ultrasound assessment in spondyloarthropathy*.* *Ann Rheum Dis* 2009;**68**(2):169-74 doi: 10.1136/ard.2007.084251.

6. Mease P.J., Liu M., Rebello S., Hua W., McLean R.R., Yi E., et al. Characterization of Patients With Axial Spondyloarthritis by Enthesitis Presence: Data from the Corrona Psoriatic Arthritis/Spondyloarthritis Registry*.* *ACR Open Rheumatol* 2020;**2**(7):449-456 doi: 10.1002/acr2.11154.

7. Sunar I., Ataman S., Nas K., Kilic E., Sargin B., Kasman S.A., et al. Enthesitis and its relationship with disease activity, functional status, and quality of life in psoriatic arthritis: a multi-center study*.* *Rheumatol Int* 2020;**40**(2):283-294 doi: 10.1007/s00296-019-04480-9.

8. Strand V., Deodhar A., Conaghan P.G., Gilloteau I., Massey O., Tian H., et al. Assessing the humanistic and economic burden of enthesitis among patients with peripheral and axial spondyloarthritis: Results from a multi-national real world survey database*.* *Arthritis and Rheumatology* 2019;**71**:1082-1085 doi: 10.1002/art.41108.

9. Behrens F., Sewerin P., De Miguel E., Patel Y., Batalov A., Dokoupilova E., et al. Achilles tendon enthesitis and disease burden in psoriatic arthritis and axial spondyloarthritis: baseline results from a randomized controlled trial*.* *Arthritis & rheumatology* 2019;**71**:4432‐4434 doi: 10.1002/art.41108.

10. Kwan Y.H., Fong W., Leung Y.Y., Lui N.L., Tan C.S., Malhotra R., et al. Are extra-spinal symptoms associated with quality of life in patients with axial spondyloarthritis? A 1-year follow-up study*.* *Clin Rheumatol* 2019;**38**(7):1881-1887 doi: 10.1007/s10067-019-04514-4.

11. de Winter J.J., Paramarta J.E., de Jong H.M., van de Sande M.G., and Baeten D.L. Peripheral disease contributes significantly to the level of disease activity in axial spondyloarthritis*.* *RMD Open* 2019;**5**(1):e000802 doi: 10.1136/rmdopen-2018-000802.

12. Laatiris A., Amine B., Ibn Yacoub Y., and Hajjaj-Hassouni N. Enthesitis and its relationships with disease parameters in Moroccan patients with ankylosing spondylitis*.* *Rheumatol Int* 2012;**32**(3):723-7 doi: 10.1007/s00296-010-1658-0.

13. Turan Y., Duruoz M.T., and Cerrahoglu L. Quality of life in patients with ankylosing spondylitis: a pilot study*.* *Rheumatol Int* 2007;**27**(10):895-9 doi: 10.1007/s00296-007-0315-8.

14. Solmaz D., Bakirci S., Jibri Z., Sampaio M., Karsh J., and Aydin S.Z. Psoriasis is an independent risk factor for entheseal damage in axial spondyloarthritis*.* *Semin Arthritis Rheum* 2020;**50**(1):42-47 doi: 10.1016/j.semarthrit.2019.06.016.

15. Ruyssen-Witrand A., Jamard B., Cantagrel A., Nigon D., Loeuille D., Degboe Y., et al. Relationships between ultrasound enthesitis, disease activity and axial radiographic structural changes in patients with early spondyloarthritis: Data from DESIR cohort*.* *RMD Open* 2017;**3**(2) doi: 10.1136/rmdopen-2017-000482.

16. Polachek A., Li S., Chandran V., and Gladman D.D. Clinical Enthesitis in a Prospective Longitudinal Psoriatic Arthritis Cohort: Incidence, Prevalence, Characteristics, and Outcome*.* *Arthritis Care Res (Hoboken)* 2017;**69**(11):1685-1691 doi: 10.1002/acr.23174.

17. Costantino F., Zeboulon N., Said-Nahal R., and Breban M. Radiographic sacroiliitis develops predictably over time in a cohort of familial spondyloarthritis followed longitudinally*.* *Rheumatology (Oxford)* 2017;**56**(5):811-817 doi: 10.1093/rheumatology/kew496.

18. Aydin S.Z., Can M., Alibaz-Oner F., Keser G., Kurum E., Inal V., et al. A relationship between spinal new bone formation in ankylosing spondylitis and the sonographically determined Achilles tendon enthesophytes*.* *Rheumatol Int* 2016;**36**(3):397-404 doi: 10.1007/s00296-015-3360-8.

19. Poddubnyy D., Haibel H., Listing J., Marker-Hermann E., Zeidler H., Braun J., et al. Baseline radiographic damage, elevated acute-phase reactant levels, and cigarette smoking status predict spinal radiographic progression in early axial spondylarthritis*.* *Arthritis Rheum* 2012;**64**(5):1388-98 doi: 10.1002/art.33465.

20. Klauser A.S., Wipfler E., Dejaco C., Moriggl B., Duftner C., and Schirmer M. Diagnostic values of history and clinical examination to predict ultrasound signs of chronic and acute enthesitis*.* *Clin Exp Rheumatol* 2008;**26**(4):548-53.

21. Fiorenza A., Bonitta G., Gerratana E., Marino F., Sarzi-Puttini P., Salaffi F., et al. Assessment of enthesis in patients with psoriatic arthritis and fibromyalgia using clinical examination and ultrasound*.* *Clin Exp Rheumatol* 2020;**38 Suppl 123**(1):31-39.

22. Macchioni P., Salvarani C., Possemato N., Gutierrez M., Grassi W., Gasparini S., et al. Ultrasonographic and Clinical Assessment of Peripheral Enthesitis in Patients with Psoriatic Arthritis, Psoriasis, and Fibromyalgia Syndrome: The ULISSE Study*.* *J Rheumatol* 2019;**46**(8):904-911 doi: 10.3899/jrheum.171411.

23. Zhang H., Liang J., Qiu J., Wang F., and Sun L. Ultrasonographic evaluation of enthesitis in patients with ankylosing spondylitis*.* *Journal of Biomedical Research* 2017;**31**(2):162-169 doi: 10.7555/JBR.31.20160088.

24. Michelsen B., Diamantopoulos A.P., Soldal D.M., Hammer H.B., Kavanaugh A., and Haugeberg G. Achilles enthesitis defined by ultrasound is not associated with clinical enthesitis in patients with psoriatic arthritis*.* *RMD Open* 2017;**3**(2):e000486 doi: 10.1136/rmdopen-2017-000486.

25. Althoff C.E., Sieper J., Song I.H., Weiß A., Diekhoff T., Haibel H., et al. Comparison of Clinical Examination versus Whole-body Magnetic Resonance Imaging of Enthesitis in Patients with Early Axial Spondyloarthritis during 3 Years of Continuous Etanercept Treatment*.* *J Rheumatol* 2016;**43**(3):618-24 doi: 10.3899/jrheum.150659.

26. Poggenborg R.P., Eshed I., Østergaard M., Sørensen I.J., Møller J.M., Madsen O.R., et al. Enthesitis in patients with psoriatic arthritis, axial spondyloarthritis and healthy subjects assessed by 'head-to-toe' whole-body MRI and clinical examination*.* *Ann Rheum Dis* 2015;**74**(5):823-9 doi: 10.1136/annrheumdis-2013-204239.

27. Bandinelli F., Prignano F., Bonciani D., Bartoli F., Collaku L., Candelieri A., et al. Ultrasound detects occult entheseal involvement in early psoriatic arthritis independently of clinical features and psoriasis severity*.* *Clin Exp Rheumatol* 2013;**31**(2):219-24.

28. Spadaro A., Iagnocco A., Perrotta F.M., Modesti M., Scarno A., and Valesini G. Clinical and ultrasonography assessment of peripheral enthesitis in ankylosing spondylitis*.* *Rheumatology (Oxford)* 2011;**50**(11):2080-6 doi: 10.1093/rheumatology/ker284.

29. Ruta S., Gutierrez M., Pena C., García M., Arturi A., Filippucci E., et al. Prevalence of subclinical enthesopathy in patients with spondyloarthropathy: an ultrasound study*.* *J Clin Rheumatol* 2011;**17**(1):18-22 doi: 10.1097/RHU.0b013e318204a6f8.

30. Genc H., Cakit B.D., Tuncbilek I., and Erdem H.R. Ultrasonographic evaluation of tendons and enthesal sites in rheumatoid arthritis: comparison with ankylosing spondylitis and healthy subjects*.* *Clin Rheumatol* 2005;**24**(3):272-7 doi: 10.1007/s10067-004-0997-1.

31. Balint P.V., Kane D., Wilson H., McInnes I.B., and Sturrock R.D. Ultrasonography of entheseal insertions in the lower limb in spondyloarthropathy*.* *Ann Rheum Dis* 2002;**61**(10):905-10 doi: 10.1136/ard.61.10.905.

32. Wiell C., Szkudlarek M., Hasselquist M., Møller J.M., Nørregaard J., Terslev L., et al. Power Doppler ultrasonography of painful Achilles tendons and entheses in patients with and without spondyloarthropathy: a comparison with clinical examination and contrast-enhanced MRI*.* *Clin Rheumatol* 2013;**32**(3):301-8 doi: 10.1007/s10067-012-2111-4.

33. Heuft-Dorenbosch L., Spoorenberg A., Van Tubergen A., Landewé R., Van Der Tempel H., Mielants H., et al. Assessment of enthesitis in ankylosing spondylitis*.* *Annals of the Rheumatic Diseases* 2003;**62**(2):127-132 doi: 10.1136/ard.62.2.127.

34. Healy P.J. and Helliwell P.S. Measuring clinical enthesitis in psoriatic arthritis: assessment of existing measures and development of an instrument specific to psoriatic arthritis*.* *Arthritis Rheum* 2008;**59**(5):686-91 doi: 10.1002/art.23568.

35. Hamdi W., Chelli-Bouaziz M., Ahmed M.S., Ghannouchi M.M., Kaffel D., Ladeb M.F., et al. Correlations among clinical, radiographic, and sonographic scores for enthesitis in ankylosing spondylitis*.* *Joint Bone Spine* 2011;**78**(3):270-4 doi: 10.1016/j.jbspin.2010.09.010.

36. Seven S., Pedersen S.J., Østergaard M., Felbo S.K., Sørensen I.J., Døhn U.M., et al. Peripheral Enthesitis Detected by Ultrasonography in Patients With Axial Spondyloarthritis—Anatomical Distribution, Morphology, and Response to Tumor Necrosis Factor-Inhibitor Therapy*.* *Frontiers in Medicine* 2020;**7** doi: 10.3389/fmed.2020.00341.

37. Lee S.H., Park W., Won Lee S., Kim H.A., Choe J.Y., Lee S.H., et al. Frequency of peripheral diseases in Korean patients with ankylosing spondylitis and the effectiveness of adalimumab*.* *Int J Rheum Dis* 2020;**23**(9):1175-1183 doi: 10.1111/1756-185x.13917.

38. Gladman D.D., Kavanaugh A., Gómez-Reino J.J., Wollenhaupt J., Cutolo M., Schett G., et al. Therapeutic benefit of apremilast on enthesitis and dactylitis in patients with psoriatic arthritis: a pooled analysis of the PALACE 1-3 studies*.* *RMD Open* 2018;**4**(1):e000669 doi: 10.1136/rmdopen-2018-000669.

39. Hartung W., Nigg A., Strunk J., and Wolff B. Clinical assessment and ultrasonography in the follow-up of enthesitis in patients with spondyloarthritis: a multicenter ultrasound study in daily clinical practice*.* *Open Access Rheumatol* 2018;**10**:161-169 doi: 10.2147/oarrr.S179472.

40. van der Heijde D., Braun J., Deodhar A., Inman R.D., Xu S., Mack M.E., et al. Comparison of three enthesitis indices in a multicentre, randomized, placebo-controlled trial of golimumab in ankylosing spondylitis (GO-RAISE)*.* *Rheumatology (Oxford)* 2013;**52**(2):321-5 doi: 10.1093/rheumatology/kes251.

41. Rudwaleit M., Claudepierre P., Kron M., Kary S., Wong R., and Kupper H. Effectiveness of adalimumab in treating patients with ankylosing spondylitis associated with enthesitis and peripheral arthritis*.* *Arthritis Res Ther* 2010;**12**(2):R43 doi: 10.1186/ar2953.

42. Alcalde M., Acebes J.C., Cruz M., González-Hombrado L., Herrero-Beaumont G., and Sánchez-Pernaute O. A sonographic enthesitic index of lower limbs is a valuable tool in the assessment of ankylosing spondylitis*.* *Ann Rheum Dis* 2007;**66**(8):1015-9 doi: 10.1136/ard.2006.062174.

43. Ozsoy-Unubol T. and Yagci I. Is ultrasonographic enthesitis evaluation helpful for diagnosis of non-radiographic axial spondyloarthritis? *Rheumatol Int* 2018;**38**(11):2053-2061 doi: 10.1007/s00296-018-4164-4.

44. Aydin S.Z., Filippucci E., Atagunduz P., Yavuz S., Grassi W., and Direskeneli H. Sonographic measurement of Achilles tendon thickness in seronegative spondyloarthropathies*.* *Eur J Rheumatol* 2014;**1**(1):7-10 doi: 10.5152/eurjrheum.2014.002.

45. Feydy A., Lavie-Brion M.C., Gossec L., Lavie F., Guerini H., Nguyen C., et al. Comparative study of MRI and power Doppler ultrasonography of the heel in patients with spondyloarthritis with and without heel pain and in controls*.* *Ann Rheum Dis* 2012;**71**(4):498-503 doi: 10.1136/annrheumdis-2011-200336.

46. Hu Y., Zhu J., Xue Q., Wang N., and Hu B. Scanning of the sacroiliac joint and entheses by color Doppler ultrasonography in patients with ankylosing spondylitis*.* *J Rheumatol* 2011;**38**(8):1651-5 doi: 10.3899/jrheum.101366.

47. Ishida S.N., Furtado R.N.V., Rosenfeld A., Proglhof J.E.P., Estrela G.B.Q., and Natour J. Ultrasound of entheses in ankylosing spondylitis patients: The importance of the calcaneal and quadriceps entheses for differentiating patients from healthy individuals*.* *Clinics (Sao Paulo)* 2019;**74**:e727 doi: 10.6061/clinics/2019/e727.

48. Ruta S., Acosta Felquer M.L., Rosa J., Navarta D.A., Garcia Monaco R., and Soriano E.R. Responsiveness to therapy change of a global ultrasound assessment in spondyloarthritis patients*.* *Clin Rheumatol* 2015;**34**(1):125-32 doi: 10.1007/s10067-014-2673-4.

49. Falcao S., Castillo-Gallego C., Peiteado D., Branco J., Martin Mola E., and de Miguel E. Can we use enthesis ultrasound as an outcome measure of disease activity in spondyloarthritis? A study at the Achilles level*.* *Rheumatology (Oxford)* 2015;**54**(9):1557-62 doi: 10.1093/rheumatology/keu399.

50. Aydin S.Z., Karadag O., Filippucci E., Atagunduz P., Akdogan A., Kalyoncu U., et al. Monitoring Achilles enthesitis in ankylosing spondylitis during TNF-alpha antagonist therapy: an ultrasound study*.* *Rheumatology (Oxford)* 2010;**49**(3):578-82 doi: 10.1093/rheumatology/kep410.

51. Naredo E., Batlle-Gualda E., García-Vivar M.L., García-Aparicio A.M., Fernández-Sueiro J.L., Fernández-Prada M., et al. Power Doppler ultrasonography assessment of entheses in spondyloarthropathies: response to therapy of entheseal abnormalities*.* *J Rheumatol* 2010;**37**(10):2110-7 doi: 10.3899/jrheum.100136.

52. Harman H. and Suleyman E. Features of the Achilles tendon, paratenon, and enthesis in inflammatory rheumatic diseases : A clinical and ultrasonographic study*.* *Z Rheumatol* 2018;**77**(6):511-521 doi: 10.1007/s00393-017-0314-4.

53. Baraliakos X., Kiltz U., Appel H., Dybowski F., Igelmann M., Kalthoff L., et al. Chronic but not inflammatory changes at the Achilles' tendon differentiate patients with peripheral spondyloarthritis from other diagnoses - Results from a prospective clinical trial*.* *RMD Open* 2017;**3**(2):e000541 doi: 10.1136/rmdopen-2017-000541.

54. Wink F., Bruyn G.A., Maas F., Griep E.N., van der Veer E., Bootsma H., et al. Ultrasound Evaluation of the Entheses in Daily Clinical Practice during Tumor Necrosis Factor-α Blocking Therapy in Patients with Ankylosing Spondylitis*.* *J Rheumatol* 2017;**44**(5):587-593 doi: 10.3899/jrheum.160584.

55. Sudoł-Szopińska I., Zaniewicz-Kaniewska K., and Kwiatkowska B. Spectrum of ultrasound pathologies of achilles tendon, plantar Aponeurosis and flexor digiti brevis tendon heel entheses in patients with clinically suspected enthesitis*.* *Polish Journal of Radiology* 2014;**79**(1):402-408 doi: 10.12659/PJR.890803.

56. Hamdi W., Bouaziz Chelli M., Ghannouchi M.M., Hawel M., Ladeb M.F., and Kchir M.M. Performance of ultrasounds compared with radiographs to detect chronic enthesitis signs in patients with ankylosing spondylitis*.* *Rheumatol Int* 2013;**33**(2):497-9 doi: 10.1007/s00296-011-2174-6.

57. Kiris A., Kaya A., Ozgocmen S., and Kocakoc E. Assessment of enthesitis in ankylosing spondylitis by power Doppler ultrasonography*.* *Skeletal Radiol* 2006;**35**(7):522-8 doi: 10.1007/s00256-005-0071-3.

58. D'Agostino M.A., Said-Nahal R., Hacquard-Bouder C., Brasseur J.L., Dougados M., and Breban M. Assessment of peripheral enthesitis in the spondylarthropathies by ultrasonography combined with power Doppler: a cross-sectional study*.* *Arthritis Rheum* 2003;**48**(2):523-33 doi: 10.1002/art.10812.

59. Lanfranchi M.A., Leluc O., Tavano A., Wormser C., Morange S., Chagnaud C., et al. Are Ultrasound Findings Similar in Patients with Axial Spondyloarthritis and in Athlete Entheses? *J Rheumatol* 2017;**44**(5):609-612 doi: 10.3899/jrheum.160715.

60. Wang C.H., Feng Y., Ren Z., Yang X., Jia J.F., Rong M.Y., et al. Performance of ultrasound to monitor Achilles enthesitis in patients with ankylosing spondylitis during TNF-a antagonist therapy*.* *Clin Rheumatol* 2015;**34**(6):1073-8 doi: 10.1007/s10067-015-2939-5.

61. Borman P., Koparal S., Babaoğlu S., and Bodur H. Ultrasound detection of entheseal insertions in the foot of patients with spondyloarthropathy*.* *Clin Rheumatol* 2006;**25**(3):373-7 doi: 10.1007/s10067-005-0036-x.

62. Kleinrensink N.J., Foppen W., Ten Katen I., van der Veen P.H., de Klerk B., Diepstraten S.C.E., et al. Comparison of the Heel Enthesitis MRI Scoring System (HEMRIS) with clinical enthesitis and local metabolic activity on PET-CT*.* *RMD Open* 2020;**6**(3) doi: 10.1136/rmdopen-2020-001424.

63. Renson T., Carron P., De Craemer A.S., Deroo L., de Hooge M., Krabbe S., et al. The Value of Magnetic Resonance Imaging for Assessing Disease Extent and Prediction of Relapse in Early Peripheral Spondyloarthritis*.* *Arthritis Rheumatol* 2021;**73**(11):2044-2051 doi: 10.1002/art.41783.

64. Aguila Maldonado R., Ruta S., Valuntas M.L., and García M. Ultrasonography assessment of heel entheses in patients with spondyloarthritis: a comparative study with magnetic resonance imaging and conventional radiography*.* *Clin Rheumatol* 2017;**36**(8):1811-1817 doi: 10.1007/s10067-017-3723-5.

65. Lorenzin M., Ortolan A., Frallonardo P., Vio S., Lacognata C., Oliviero F., et al. Spine and sacroiliac joints on magnetic resonance imaging in patients with early axial spondyloarthritis: prevalence of lesions and association with clinical and disease activity indices from the Italian group of the SPACE study*.* *Reumatismo* 2016;**68**(2):72-82 doi: 10.4081/reumatismo.2016.885.

66. Herregods N., Dehoorne J., Pattyn E., Jaremko J.L., Baraliakos X., Elewaut D., et al. Diagnositic value of pelvic enthesitis on MRI of the sacroiliac joints in enthesitis related arthritis*.* *Pediatr Rheumatol Online J* 2015;**13**(1):46 doi: 10.1186/s12969-015-0045-5.

67. Klang E., Aharoni D., Hermann K.G., Herman A., Rimon U., Shazar N., et al. Magnetic resonance imaging of pelvic entheses--a systematic comparison between short tau inversion recovery (STIR) and T1-weighted, contrast-enhanced, fat-saturated sequences*.* *Skeletal Radiol* 2014;**43**(4):499-505 doi: 10.1007/s00256-013-1814-1.

68. Aydin S.Z., Tan A.L., Hodsgon R., Grainger A., Emery P., Wakefield R.J., et al. Comparison of ultrasonography and magnetic resonance imaging for the assessment of clinically defined knee enthesitis in spondyloarthritis*.* *Clin Exp Rheumatol* 2013;**31**(6):933-6.

69. Jans L., van Langenhove C., Van Praet L., Carron P., Elewaut D., Van Den Bosch F., et al. Diagnostic value of pelvic enthesitis on MRI of the sacroiliac joints in spondyloarthritis*.* *Eur Radiol* 2014;**24**(4):866-71 doi: 10.1007/s00330-013-3074-9.

70. Paramarta J.E., van der Leij C., Gofita I., Yeremenko N., van de Sande M.G., de Hair M.J., et al. Peripheral joint inflammation in early onset spondyloarthritis is not specifically related to enthesitis*.* *Ann Rheum Dis* 2014;**73**(4):735-40 doi: 10.1136/annrheumdis-2012-203155.

71. Emad Y., Ragab Y., Shaarawy A., Abou-Zeid A., Saad A., Fawzy M., et al. Can magnetic resonance imaging differentiate undifferentiated arthritis based on knee imaging? *J Rheumatol* 2009;**36**(9):1963-70 doi: 10.3899/jrheum.081320.

72. Kamel M., Eid H., and Mansour R. Ultrasound detection of knee patellar enthesitis: A comparison with magnetic resonance imaging*.* *Annals of the Rheumatic Diseases* 2004;**63**(2):213-214 doi: 10.1136/ard.2003.010314.

73. Krabbe S., Eshed I., Sorensen I.J., Moller J., Jensen B., Madsen O.R., et al. Novel whole-body magnetic resonance imaging response and remission criteria document diminished inflammation during golimumab treatment in axial spondyloarthritis*.* *Rheumatology (Oxford)* 2020;**59**(11):3358-3368 doi: 10.1093/rheumatology/keaa153.

74. Poulsen A.E.F., Axelsen M.B., Poggenborg R.P., Eshed I., Krabbe S., Glinatsi D., et al. Whole-body Magnetic Resonance Imaging in Psoriatic Arthritis, Rheumatoid Arthritis, and Healthy Controls: Interscan, Intrareader, and Interreader Agreement and Distribution of Lesions*.* *J Rheumatol* 2021;**48**(2):198-206 doi: 10.3899/jrheum.200084.

75. Krabbe S., Eshed I., Sorensen I.J., Jensen B., Moller J.M., Balding L., et al. Whole-body Magnetic Resonance Imaging Inflammation in Peripheral Joints and Entheses in Axial Spondyloarthritis: Distribution and Changes during Adalimumab Treatment*.* *J Rheumatol* 2020;**47**(1):50-58 doi: 10.3899/jrheum.181159.

76. Krabbe S., Eshed I., Gandjbakhch F., Pedersen S.J., Bird P., Mathew A.J., et al. Development and Validation of an OMERACT MRI Whole-Body Score for Inflammation in Peripheral Joints and Entheses in Inflammatory Arthritis (MRI-WIPE)*.* *J Rheumatol* 2019;**46**(9):1215-1221 doi: 10.3899/jrheum.181084.

77. Krabbe S., Østergaard M., Eshed I., Sørensen I.J., Jensen B., Møller J.M., et al. Whole-body Magnetic Resonance Imaging in Axial Spondyloarthritis: Reduction of Sacroiliac, Spinal, and Entheseal Inflammation in a Placebo-controlled Trial of Adalimumab*.* *J Rheumatol* 2018;**45**(5):621-629 doi: 10.3899/jrheum.170408.

78. Poggenborg R.P., Pedersen S.J., Eshed I., Sorensen I.J., Moller J.M., Madsen O.R., et al. Head-to-toe whole-body MRI in psoriatic arthritis, axial spondyloarthritis and healthy subjects: first steps towards global inflammation and damage scores of peripheral and axial joints*.* *Rheumatology (Oxford)* 2015;**54**(6):1039-49 doi: 10.1093/rheumatology/keu439.

79. Karpitschka M., Godau-Kellner P., Kellner H., Horng A., Theisen D., Glaser C., et al. Assessment of therapeutic response in ankylosing spondylitis patients undergoing anti-tumour necrosis factor therapy by whole-body magnetic resonance imaging*.* *Eur Radiol* 2013;**23**(7):1773-84 doi: 10.1007/s00330-013-2794-1.

80. Althoff C.E., Sieper J., Song I.H., Haibel H., Weiss A., Diekhoff T., et al. Active inflammation and structural change in early active axial spondyloarthritis as detected by whole-body MRI*.* *Ann Rheum Dis* 2013;**72**(6):967-73 doi: 10.1136/annrheumdis-2012-201545.

81. Weber U., Lambert R.G., Rufibach K., Maksymowych W.P., Hodler J., Zejden A., et al. Anterior chest wall inflammation by whole-body magnetic resonance imaging in patients with spondyloarthritis: lack of association between clinical and imaging findings in a cross-sectional study*.* *Arthritis Res Ther* 2012;**14**(1):R3 doi: 10.1186/ar3551.

82. Song I.H., Hermann K., Haibel H., Althoff C.E., Listing J., Burmester G., et al. Effects of etanercept versus sulfasalazine in early axial spondyloarthritis on active inflammatory lesions as detected by whole-body MRI (ESTHER): a 48-week randomised controlled trial*.* *Ann Rheum Dis* 2011;**70**(4):590-6 doi: 10.1136/ard.2010.139667.

83. Weckbach S., Schewe S., Michaely H.J., Steffinger D., Reiser M.F., and Glaser C. Whole-body MR imaging in psoriatic arthritis: additional value for therapeutic decision making*.* *Eur J Radiol* 2011;**77**(1):149-55 doi: 10.1016/j.ejrad.2009.06.020.

84. Dallaudière B., Trotier A., Ribot E., Verdier D., Lepreux S., Miraux S., et al. Three-dimensional ultrashort echo time (3D UTE) MRI of Achilles tendon at 4.7T MRI with comparison to conventional sequences in an experimental murine model of spondyloarthropathy*.* *J Magn Reson Imaging* 2019;**50**(1):127-135 doi: 10.1002/jmri.26569.

85. Chen B., Cheng X., Dorthe E.W., Zhao Y., D'Lima D., Bydder G.M., et al. Evaluation of normal cadaveric Achilles tendon and enthesis with ultrashort echo time (UTE) magnetic resonance imaging and indentation testing*.* *NMR Biomed* 2019;**32**(1):e4034 doi: 10.1002/nbm.4034.

86. Chen B., Zhao Y., Cheng X., Ma Y., Chang E.Y., Kavanaugh A., et al. Three-dimensional ultrashort echo time cones (3D UTE-Cones) magnetic resonance imaging of entheses and tendons*.* *Magn Reson Imaging* 2018;**49**:4-9 doi: 10.1016/j.mri.2017.12.034.

87. Du J., Chiang A.J., Chung C.B., Statum S., Znamirowski R., Takahashi A., et al. Orientational analysis of the Achilles tendon and enthesis using an ultrashort echo time spectroscopic imaging sequence*.* *Magn Reson Imaging* 2010;**28**(2):178-84 doi: 10.1016/j.mri.2009.06.002.

88. Sung S., Kim H.S., and Kwon J.W. MRI assessment of sacroiliitis for the diagnosis of axial spondyloarthropathy: comparison of fat-saturated T2, STIR and contrast-enhanced sequences*.* *Br J Radiol* 2017;**90**(1078):20170090 doi: 10.1259/bjr.20170090.

89. de Hooge M., van den Berg R., Navarro-Compán V., van Gaalen F., van der Heijde D., Huizinga T., et al. Magnetic resonance imaging of the sacroiliac joints in the early detection of spondyloarthritis: no added value of gadolinium compared with short tau inversion recovery sequence*.* *Rheumatology (Oxford)* 2013;**52**(7):1220-4 doi: 10.1093/rheumatology/ket012.

90. Maksymowicz H., Kowalewski K., Lubkowska K., Zołud W., and Sąsiadek M. Diagnostic value of gadolinium-enhanced MR imaging of active sacroiliitis in seronegative spondyloarthropathy*.* *Pol J Radiol* 2010;**75**(2):58-65.

91. Mathew A.J., Krabbe S., Eshed I., Lambert R.G., Laredo J.D., Maksymowych W.P., et al. Atlas of the OMERACT Heel Enthesitis MRI Scoring System (HEMRIS)*.* *RMD Open* 2020;**6**(1) doi: 10.1136/rmdopen-2019-001150.

92. Mathew A.J., Krabbe S., Eshed I., Gandjbakhch F., Bird P., Pedersen S.J., et al. The OMERACT MRI in Enthesitis Initiative: Definitions of Key Pathologies, Suggested MRI Sequences, and a Novel Heel Enthesitis Scoring System*.* *J Rheumatol* 2019;**46**(9):1232-1238 doi: 10.3899/jrheum.181093.

93. Kim T.H., Lee J.K., Sung H.K., Kim B.H., Song Y.S., and Sung I.H. Radiologic features in symptomatic/asymptomatic heels of patients with ankylosing spondylitis*.* *Int J Rheum Dis* 2019;**22**(2):222-227 doi: 10.1111/1756-185X.13379.

94. Helliwell P.S. and Porter G. Sensitivity and specificity of plain radiographic features of peripheral enthesopathy at major sites in psoriatic arthritis*.* *Skeletal Radiol* 2007;**36**(11):1061-6 doi: 10.1007/s00256-007-0376-5.

95. Taylor W.J., Porter G.G., and Helliwell P.S. Operational definitions and observer reliability of the plain radiographic features of psoriatic arthritis*.* *J Rheumatol* 2003;**30**(12):2645-58.

96. Secundini R., Scheines E.J., Gusis S.E., Riopedre A.M., Citera G., and Maldonado Cocco J.A. Clinico-radiological correlation of enthesitis in seronegative spondyloarthropathies (SNSA)*.* *Clin Rheumatol* 1997;**16**(2):129-32 doi: 10.1007/bf02247840.

97. Resnick D., Feingold M.L., Curd J., Niwayama G., and Goergen T.G. Calcaneal abnormalities in articular disorders. Rheumatoid arthritis, ankylosing spondylitis, psoriatic arthritis, and Reiter syndrome*.* *Radiology* 1977;**125**(2):355-66 doi: 10.1148/125.2.355.

98. Bruijnen S.T., van der Weijden M.A., Klein J.P., Hoekstra O.S., Boellaard R., van Denderen J.C., et al. Bone formation rather than inflammation reflects ankylosing spondylitis activity on PET-CT: a pilot study*.* *Arthritis Res Ther* 2012;**14**(2):R71 doi: 10.1186/ar3792.

99. Vijayant V., Sarma M., Aurangabadkar H., Bichile L., and Basu S. Potential of (18)F-FDG-PET as a valuable adjunct to clinical and response assessment in rheumatoid arthritis and seronegative spondyloarthropathies*.* *World J Radiol* 2012;**4**(12):462-8 doi: 10.4329/wjr.v4.i12.462.
